# A robust machine learning model based on ribosomal‐subunit‐derived piRNAs for diagnostic potential of nonsmall cell lung cancer across multicentre, large‐scale of sequencing data

**DOI:** 10.1002/ctm2.70418

**Published:** 2025-07-25

**Authors:** Zitong Gao, Masaki Nasu, Gehan Devendra, Ayman A. Abdul‐Ghani, Anthony J. Herrera, Jeffrey A. Borgia, Christopher W. Seder, Donna Lee Kuehu, Zhuokun Feng, Yu Chen, Ting Gong, Zao Zhang, Owen Chan, Hua Yang, Jianhua Yu, Yuanyuan Fu, Lang Wu, Youping Deng

**Affiliations:** ^1^ Department of Quantitative Health Sciences John A. Burns School of Medicine University of Hawaii at Manoa Honolulu Hawaiʻi USA; ^2^ Molecular Biosciences and Bioengineering Program College of Tropical Agriculture and Human Resources University of Hawaii at Manoa Honolulu Hawaiʻi USA; ^3^ Genomics and Bioinformatics Shared Resource University of Hawaii Cancer Center Honolulu Hawaiʻi USA; ^4^ Department of Medicine John A. Burns School of Medicine University of Hawaii at Manoa Honolulu Hawaiʻi USA; ^5^ Critical Care Medicine, Pulmonary The Queen's Medical Center Honolulu Hawaiʻi USA; ^6^ Cardiothoracic Surgery The Queen's Medical Center, Honolulu Honolulu Hawaiʻi USA; ^7^ Interventional Radiology The Queen's Medical Center Honolulu Hawaiʻi USA; ^8^ Departments of Anatomy & Cell Biology and Pathology RUSH University Cancer Center Chicago Illinois USA; ^9^ Cardiothoracic Residency Program RUSH University Chicago Illinois USA; ^10^ Hospitalist Medicine The Queen's Medical Center Honolulu Hawaiʻi USA; ^11^ Pathology Core Shared Resource University of Hawaii Cancer Center Honolulu Hawaiʻi USA; ^12^ Institute for Precision Cancer Therapeutics and Immuno‐Oncology Chao Family Comprehensive Cancer Center University of California Irvine California USA; ^13^ Pacific Center for Genome Research University of Hawaii Cancer Center Honolulu Hawaiʻi USA

**Keywords:** machine learning, noninvasive diagnosis, nonsmall cell lung cancer, PIWI‐interacting RNA, small noncoding RNA

## Abstract

Nonsmall cell lung cancer (NSCLC) is a lethal cancer and lacks robust biomarkers for noninvasive clinical diagnosis. Detecting NSCLC at the early stage can decrease the mortality rate and minimise harm caused by various treatments. We curated 2050 samples from public tissue and plasma datasets including both invasive and noninvasive types, then supplemented with in‐house pooled plasma and exosome samples. Eleven independent transcriptome datasets were utilised to develop a new machine learning model by integrating PIWI‐interacting RNA (piRNA) to predict NSCLC. Five piRNA signatures derived from ribosomal subunits identified to be tumour‐specific exhibited robust diagnostic ability and were combined into a piRNA‐Based Tumour Probability Index (pi‐TPI) risk evaluation model. pi‐TPI effectively distinguished NSCLC patients from healthy individuals and showed efficacy in identifying early‐stage cancers with Area under the ROC Curve (AUC) values over .80. Plasma cohorts exhibited the diagnosis efficacy of pi‐TPI with an AUC value of .85. Experimental exosomal data enhances the accuracy of diagnosing noncancerous, benign, and cancer cases. The pi‐TPI marker in the noncancer/cancer subgroup exhibited superior predictive performance with an AUC value of .96. These findings underscore the significant clinical potential of the five piRNA signatures as a powerful diagnostic tool for NSCLC, particularly of noninvasive cancer diagnostics.

## Graphical abstract

NSCLC lacks robust noninvasive biomarkers. We curated 2050 invasive and noninvasive samples, integrating public and in‐house plasma/exosome data. A machine learning model using five tumour‐specific piRNA signatures formed the piRNA‐Based Tumour Probability Index (pi‐TPI), achieving AUC values up to .96, demonstrating strong potential for NSCLC diagnosis.

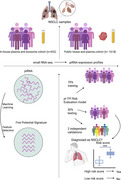

## 1 INTRODUCTION

Lung cancer, comprising mainly nonsmall cell lung cancer (NSCLC) and small cell lung cancer (SCLC) types, is a critical health concern. NSCLC is the more common form, ranking as the second leading cancer in both genders in the United States. In 2024, the American Cancer Society estimates approximately 234 580 new lung cancer cases (116 310 in men and 118 270 in women) in the United States.^1^ The death rates related to smoking cessation and treatment improvement in lung cancer have declined substantially over the last 20 years, nevertheless, the proportion of lung cancer cases occurring among never smokers is increasing.^2^ The diagnosis of lung cancer usually follows imaging tools including Computed Tomography (CT) scans, Magnetic Resonance Imaging (MRI) scans, Positron Emission Tomography‐computed Tomography (PET‐CT) scans,^3^ and invasive biopsies to determine if the cancer is present.^4^ However, lung cancer symptoms vary and can include coughing, breathlessness, and chest pain, often appearing in advanced stages. Detecting lung cancer at an early stage and initiating treatment can decrease the mortality rate and minimise treatment‐related harm.

In recent years, liquid biopsy, a noninvasive approach for sample procurement compared to tumour biopsies, represents a novel implementation in the diagnostic processes for lung cancer,^4^, ^5^, ^6^ which can greatly reduce the patient burden. It facilitates the identification, investigation, and monitoring by analysing body fluids from cancer samples including blood, serum, plasma, and urine. Several biological constituents such as platelets, circulating cells, cell‐free DNA (cf‐DNA), circulating tumour DNA (ctDNA), microRNA (miRNA), transfer RNA (tRNA), extracellular vesicles, long noncoding RNA and proteins are gaining interest for their potential diagnostic ability.^7^, ^8^, ^9^, ^10^, ^11^, ^12^, ^13^

Small noncoding RNA (sncRNA) have been extensively investigated as potential source for the development of noninvasive cancer biomarkers.^14^ Given that sncRNA are frequently upregulated or downregulated in various cancers, they can be promising candidates for tracking cancer progression and recurrence. For example, two exosomal miRNA miR‐125b‐5p and miR‐5684 were identified as downregulated in 330 NSCLC patients compared to 312 healthy donors.^15^ Another study revealed that exosomal miR‐620 in lung cancer patients presented lower expression level contrast to healthy donors.^16^

PIWI‐interacting RNAs (piRNAs), typically 24–35 nucleotides in length, were first discovered in 2001 as a class of small RNAs expressed in the testes of *Drosophila melanogaster*, where they play a critical role in spermiogenesis.^17^ These small RNAs specifically associate with PIWI proteins, a subfamily of Argonaute (AGO) RNA‐binding proteins, to form the piRNA‐induced silencing complex (piRISC). The PIWI‐piRNA pathway is regarded as the immune system of the germline, particularly through the silencing of transposable elements (TEs), which pose a major threat to genome stability during germ cell development. In many mammalian gene sequences, transposons constitute over 50% of the sequence content.^18^ Once theses TEs are freed from restrictions, the mobile elements can transpose via copy‐and‐paste or cut‐and‐paste mechanisms, inserting themselves into new genomic loci and potentially disrupting gene function or inducing mutations.^19^, ^20^ Deep sequencing and computational analyses have revealed that piRNAs are predominantly derived either from RNA transcript of active TE copies or specific genomic loci, which are termed as ‘piRNA clusters’.^21^, ^22^ These cluster‐derived piRNAs are mainly antisense to TE mRNA sequences and serve as guides for PIWI proteins to recognise TE transcripts through complementary base pairing.^23^, ^24^

Currently, numerous dysregulated piRNAs in tumour tissues have been identified as a tumour‐promoter or tumour‐suppressor, demonstrating piRNAs strongly correlated with tumour cell malignant phenotype and clinical stage.^25^ Currently, more than ten piRNAs have been reported to be biologically associated with lung cancer.^26^ One investigation found that piR‐L‐163 is notably reduced in NSCLC cells, mediate cell migration, and invasion by sustaining the activity of phosphorylated ezrin‐radixin‐moesin (p‐ERM) complexes.^27^ Another piRNA, piR‐55490, was recognised for its role in inhibiting lung cancer cell growth by attaching to the 3′ UTR region of mammalian target of rapamycin (mTOR) mRNA.^28^ piR‐651 was observed to stimulate cell proliferation and apoptosis.^29^ piR‐57125 showed a remarkable downregulated expression in lung metastasis model.^30^ Other piRNAs, specifically piR‐34871 and piR‐52200 were found upregulated by tumour promoter The Ras‐association domain family 1 (RASSF1C), whereas piR‐35127 and piR‐46545 were downregulated via the RASSF1C‐PIWILI‐piRNA axis, promoting the lung cancer stem cell proliferation, colony formation and epithelial–mesenchymal transition (EMT).^31^ These findings indicate that abnormal piRNA expressions may play a crucial role in the development, spread, and metastasis of lung cancer, offering promising new avenues for novel biomarkers in precision medicine.

Until now, there is no systematic and comprehensive study on the piRNA diagnostic biomarker from tissue to liquid biopsy has been carried out for NSCLC. Traditional methodologies in cancer biomarker diagnosis often limit to homogeneous sample types, typically either tissue or plasma samples exclusively. This research introduces a comprehensive piRNA diagnostic signature for NSCLC, utilising tissue, plasma, and exosome samples from our own experiments. This approach surpasses the limitations of traditional homogeneous sample analyses. Conformation of the diagnostic accuracy of this signature across diverse cohorts, including using exosomal samples to enhance liquid biopsy diagnosis. To improve diagnostic accuracy for liquid biopsy, we developed a piRNA Base Tumour Probability Index (pi‐TPI) to specifically evaluate malignancy risk in NSCLC. Notably, it is the first time that included noncancer, benign, and cancer samples from exosomes to develop piRNA signatures for exosomal liquid biopsy diagnosis. We established the risk assessment regarding the five piRNA signatures and it exhibited superior diagnosis ability in identifying patients with NSCLC.

## 2 RESULTS

### 2.1 Cohort assignment and study design

**FIGURE 1:**
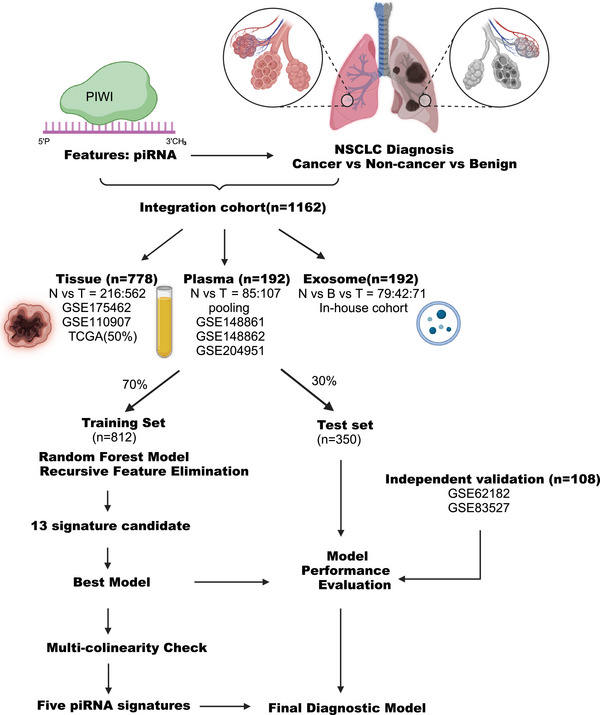
Overview of development of the model for multisource data diagnosis of NSCLC.

We collected a total of 1810 tumour, normal, and benign sequencing data from multicentres including 1426 tissue samples, 192 plasma samples (24 in‐house samples were included), and 192 in‐house exosome samples for discovery and validation studies for piRNA biomarkers in NSCLC diagnosis (Figure 1). As for the disproportionate tumour‐to‐normal ratio from TCGA‐LUAD and TCGA‐LUSC cohorts, we selectively included 182 matched pairs of tumour and normal samples, and we incorporated 40% of the remaining tumour samples. This subset was subsequently merged with further datasets (tissue: GSE175462, GSE110907; plasma: GSE148861, GSE148862, GSE204951, RUSH_pooling from Rush University, original exosome source: Cooperative Human Tissue Network (CHTN)) to form the final cohort, ensuring a balanced distribution of tumour and normal samples. RNA extraction, exosome purification and small RNA‐sequencing were done in our lab. The final cohort comprises 1162 samples, with a tumour‐to‐normal ratio of 1.75:1.

**FIGURE 2:**
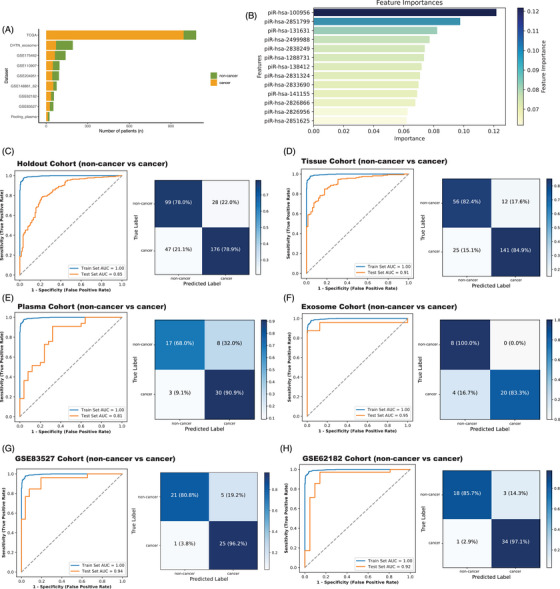
13 piRNA‐based RF model performance across tissue, plasma and exosome in test set. (A) Bar chart showing the number of patients in each cohort of cancer and noncancer category. (B) Ranking of feature importance of 13 piRNA from RF training model (*n* = 812). (C–H) ROC curves with the corresponding AUC values of RF model in the training set and holdout/tissue/plasma/exosome/independent validation, and confusion matrices showing diagnosis results generated by RF model in holdout/tissue/plasma/exosome/independent cohort.

Our dataset was randomised into two distinct groups: 70% (812 samples) were designated for the training subsample to construct the prediction algorithm, while the remaining 30% (350 samples) constituted the holdout validation subsample, utilised to evaluate the trained classifier. Other two GEO datasets (GSE62182 and GSE83527) containing tissue samples were used as independent validations for model evaluation. The proportion of sample types in cancer and noncancer can be found in Figure 2A.

### 2.2 Signature discovery in multicentre and multisource cohorts

To identify NSCLC‐associated piRNAs, we analysed over 1 million piRNA expression profiles from cancer and noncancer samples including tissue, plasma, exosome, from paired TCGA‐LUAD, TCGA‐LUSC, GEO cohorts, and exosome CHTN cohorts. The genome‐wide distribution density of all annotated piRNA is presented in Figure S1. We filtered out piRNAs with low expression, specifically those unexpressed in more than 50% of the samples, and identified 2993 piRNAs that were consistently present across all included cohorts. Among all the overlapped piRNAs, 333 piRNAs were from piRNAclusterDB, and 2769 of piRNA were from piRbase gold standard set, 19 piRNAs were identified both from piRNAclusterDB and piRbase gold standard set. Both databases are publicly accessible. To refine the number variable pool and identify the signatures that exhibit the consistent expression trend across all cohorts, we calculated differentially expressed piRNA (DEpiRNAs), and only those with consistent fold change direction were kept. We employed the Recursive Feature Elimination‐Random Forest (RFE‐RF) algorithm in combination with fivefold cross‐validation as a preliminary feature selection strategy on the training dataset. Based on the overall cross‐validation performance, we achieved an average accuracy of .7942 ± .0488 and identified 13 candidate signature piRNAs: piR‐hsa‐100956, piR‐hsa‐1288731, piR‐hsa‐131631, piR‐hsa‐138412, piR‐hsa‐141155, piR‐hsa‐2499988, piR‐hsa‐2826866, piR‐hsa‐2826956, piR‐hsa‐2831324, piR‐hsa‐2833690, piR‐hsa‐2838249, piR‐hsa‐2851625, and piR‐hsa‐2851799.

### 2.3 Model performance evaluation of NSCLC‐associated 13 piRNA signatures

Development of a RF model integrated with fivefold cross‐validation on the training data based on 13 piRNA as predictive features. Each feature contribution represented as feature importance ranked from high to low can be found in Figure 2B. The accuracy of each cohort at the default cutoff of .5 was shown in Figure S4A. The average accuracy value of the whole test set of noncancer and cancer comparison is .83.

Calculation of the area under the receiver operating characteristic (ROC) curves and precision–recall curves by using the tumour probabilities computed by the 13 piRNAs featured RF model, which exhibited superior performance where the AUC of training cohort is 1.00 and the holdout validation is .85 (Figure 2C). We also dichotomised the probabilities into predicted cancer and noncancer groups by optimising the sensitivity and specificity in each test group. The optimal threshold yielded a specificity of 78% and a sensitivity of 78.9% (Figure 2C). Further analysis involved subdividing the holdout validation cohort by sample type – tissue, plasma, and exosome.

Test tissue samples (*n* = 234) had an AUC of .91 with high sensitivity and specificity exceeding 80% (Figure 2D). The test plasma samples (*n* = 58) displayed an AUC of .81, with sensitivity above 90% but a lower specificity of 68% (Figure 2E). Test exosome samples (*n* = 58), categorised into noncancer, benign, and cancer groups, showcased exceptional AUC values of up to .95, coupled with 100% specificity and 83.3% sensitivity (Figure 2F). For normal mixed benign versus cancerous classification, the optimal threshold revealed an AUC of .69, with specificity and sensitivity at 61.8% and 79.2%, respectively (Figure S3A). In two separate validation cohorts, the model displayed robust diagnostic accuracy with high area under the curve (AUC) scores of .94 for GSE83527 and .92 for GSE62182. Specificity rates exceeded 80% in both, while sensitivity was recorded at 96.2% for GSE83527 and 97.1% for GSE62182 (Figure 2G and H).

The optimal threshold for model evaluation was determined based on Youden's index in conjunction with the ROC curve. We compared all the optimal thresholds across each subgroup, including training, hold out validation, tissue cohort, plasma cohort, exosome cohorts and independent cohorts (Figure S4B). The optimal threshold from training cohort is .56, and the lowest optimal threshold from independent validation is .51 and highest optimal threshold .68 is from plasma cohort. Therefore, an average threshold of .6 was applied across each cohort to test the discriminatory power, resulting in confusion matrices that reflected a consistent threshold (Figure S4C). Utilising a single cut point to distinguish between noncancerous and cancerous cases could lead to a lower negative‐positive rate and a higher true‐positive rate, especially in certain subgroups like plasma and exosome (comparing noncancerous/benign to cancerous, and benign to cancerous, respectively). However, tissue cohorts, the exosome subgroup comparing noncancerous to cancerous, and independent tissue cohorts maintained high sensitivity and specificity rates.

Regarding the 13 piRNA RF model, it also demonstrated high area under the precision–recall curve (AUPRC) in both training and the test set (Figure S5A, B, E, and F), with AUPRC value exceeding .90 in training, hold out validation, tissue as well as two independent validations. In the noninvasive groups, plasma cohort showed slightly lower AUPRC value at .84 (Figure S5C). Notably, the AUPRC exosome subgroup in noncancer versus cancer (CH) comparison was significantly high at .99 (Figure S5D), surpassing the other groups including benign samples. *F*1 optimal threshold of each cohort is provided in Figure S5I. Under the optimal *F*1 threshold, the model consistently achieved high positive predictive value (PPV) and negative predictive value (NPV) across holdout validation, tissue validation, plasma two independent validation (Figure S5J). Within exosomal subgroup, CH group maintained notably high performance with a PPV at .958 and NPV at .875. In contrast, the benign group with noncancer samples showed an NPV at .84 and purely benign group had an NPV at .76.

Taken together, these 13 piRNA random forest models demonstrate high predictive accuracy for NSCLC, as shown by performance metrics consistently observed across various sample types.

### 2.4 Establishment of pi‐TPI contributes to identifying NSCLC with five piRNA signatures

**FIGURE 3:**
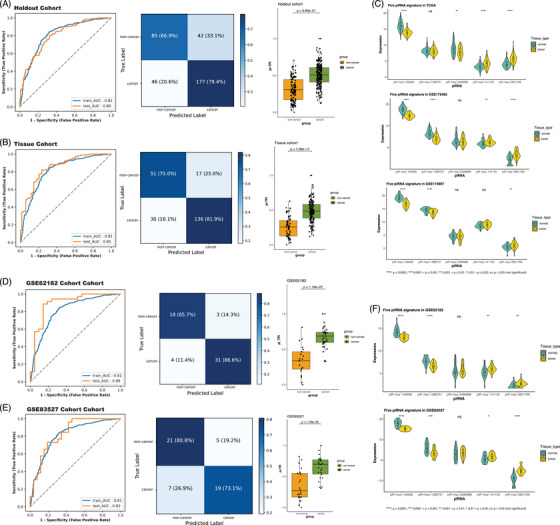
Five piRNA‐based pi‐TPI model performance in holdout, tissue and independent cohorts. (A, B) ROC curves with the corresponding AUC values of pi‐TPI model in the training set and holdout and tissue validation, and confusion matrices showing diagnosis results generated by pi‐TPI model in holdout and tissue validation. Boxplots showing the transformed risk score from pi‐TPI model in cancer and noncancer group in holdout and tissue cohort. Two‐sided *p* values were calculated using Mann–Whitney *U* test. (C) Normalised five piRNA expression level in TCGA, GSE175462, GSE110907 (tissue dataset used in training set) cohort between cancer and noncancer. (D, E) ROC curves with the corresponding AUC values of pi‐TPI model in the training set and two independent validations, GSE62182 and GSE83527, and confusion matrices showing diagnosis results generated by pi‐TPI model in two independent validations. Boxplots showing the transformed risk score from pi‐TPI model in cancer and noncancer group in each cohort. Two‐sided *p* values were calculated using Mann–Whitney *U* test. (F) Normalised five piRNA expression level in GSE62182 and GSE83527 dataset between cancer and noncancer.

To build a clinically generalisable piRNA‐based model for predicting malignancy risk probabilities in NSCLC, we developed a piRNA Base Tumour Probability Index (pi‐TPI). Five piRNA signatures (piR‐hsa‐100956, piR‐hsa‐1288731, piR‐hsa‐141155, piR‐hsa‐2499988, piR‐hsa‐2851799) were selected based on low collinearity and incorporated into a logistic regression model in the training set with correlation coefficients which were less than .8. Among the five piRNAs, piR‐hsa‐100956, piR‐hsa‐1288731, and piR‐hsa‐2499988 were downregulated while piR‐hsa‐141155 and piR‐hsa‐2851799 were upregulated (Figure 3C and F). The model was then validated across additional test sets, with each probability outcome being converted into a risk score, referred to as pi‐TPI.

**FIGURE 4:**
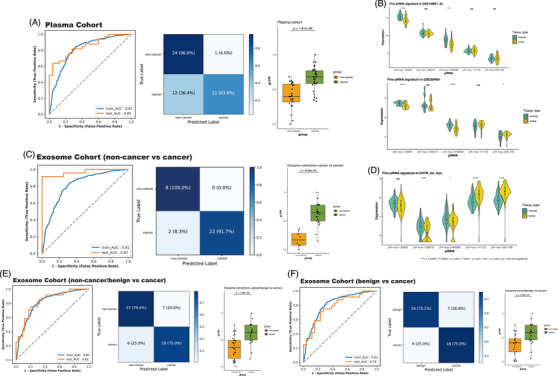
Five piRNA‐based pi‐TPI model performance in plasma and exosome cohorts. (A) ROC curves with the corresponding AUC values of pi‐TPI model in the training set and plasma validation, and confusion matrices showing diagnosis results generated by pi‐TPI model in plasma validation. Boxplots showing the transformed risk score from pi‐TPI model in cancer and noncancer group in plasma cohort. Two‐sided *p* values were calculated using Mann–Whitney *U* test. (B) Normalised five piRNA expression level in two plasma datasets, GSE204951, GSE148861/GSE148862(merged) cohort between cancer and noncancer. (C) ROC curves with the corresponding AUC values of pi‐TPI model in the training set and exosome with its subgroups, noncancer vs. cancer. Boxplots showing the transformed risk score from pi‐TPI model in cancer and noncancer group in each subgroup. Two‐sided *p* values were calculated using Mann–Whitney *U* test. (D) Normalised five piRNA expression level in exosome between cancer and noncancer. (E, F) ROC curves with the corresponding AUC values of pi‐TPI model in the training set and exosome subgroups noncancer/benign vs. cancer and benign vs. cancer, and confusion matrices showing diagnosis results generated by pi‐TPI model in each subgroup. Boxplots showing the transformed risk score from pi‐TPI model in cancer and noncancer group in each subgroup. Two‐sided *p* values were calculated using Mann–Whitney *U* test.

The performance of the logistic regression model was assessed using the ROC curve, precision–recall curve, and confusion matrices under its optimal threshold. The holdout cohort, tissue cohort, and two independent validation cohorts all exhibited AUC values exceeding .80 (Figure 3A and B). When compared to the 13 piRNAs RF model, the logistic regression model displayed marginally lower AUC values across both the overall source samples and tissue‐specific samples. Nonetheless, the AUC for the plasma cohort increased to .85 (Figure 4A). The sensitivity and specificity observed in each cohort were marginally lower for the pi‐TPI compared to the RF model but remained substantial, particularly regarding the high true positive rate. For instance, the GSE62182 cohort demonstrated a notable sensitivity rate of 88.6% (Figure 3D) and specificity rate of 85.7%. However, the plasma cohort presented a lower positive case identification rate, but higher specificity case compared to RF model. In the exosomal cohorts, the subgroup comparing noncancer to cancer cases retained an impressive AUC of .97 (Figure 4C). The subgroup that included both noncancer and benign cases achieved an AUC of up to .82, while the purely benign group reached an AUC of .78, representing a marked improvement over the original 13 RF model (Figure 4E and F). Notably, in the exosome subgroups containing benign samples, specificity rate increased from .618 to .79 and NPV improved from .84 to .88. These findings suggest enhanced model robustness in distinguishing benign conditions from malignancies.

In addition, pi‐TPI used as a risk score that was applied on each sample based on logistic regression was transformed into the format of a boxplot in each cohort. Significant differences were presented between noncancer and cancer in each subgroup (Figures 3A, B, D, and E and 4A, C, and D). Additional performance evaluations, including AUPRC, NPV, PPV, *F*1 score, and calibration curves, are presented in Figures S6 and S7. Notably, the calibration curves provide key insights into the model's performance. In both the holdout and tissue cohorts, the predicted probabilities closely aligned with the actual event rates, with most points falling near the diagonal, indicating reliable probability estimates. In the plasma cohort, calibration was particularly strong in the low‐to‐mid probability range (0–.4), while in the exosome CH cohort, predictions in the mid‐to‐high range (.6–1.0) were well‐calibrated and closely followed the ideal line. The calibration curves from plasma and exosome BC may deviate from the ideal line, likely due to the limitation of sample size. Nevertheless, most cohorts can demonstrate a reliable probability estimation, particularly in mid‐to‐high range.

We further developed sample type‐specific models by stratifying tissue, plasma, and exosome samples from the training cohort, retraining models within each subgroup and applying the pi‐TPI framework to predict malignancy risk probabilities. Each cohort was split into training and test sets using 7:3 ratio. Interestingly, the AUC or the AUPRC values didn't significantly surpass the performance it achieved in the 13 piRNA RF model. Their performance remained within a similar range compared to the overall training model and exhibited more stable behaviour across validation. This consistency highlights the robustness and reliability of pi‐TPI risk score for NSCLC prediction across different sample types, from tissue to exosome. Figures S8–S10 included AUC, AUPRC, confusion matrices, *F*1‐score, NPV, PPV and calibration curves from this specific model evaluation. For instance, in the tissue specific model, the training AUC was .88 (holdout: .81) and test AUC was .85 (test tissue: .85), while the training AUPRC and test AUPRC is .95 (holdout: .86) and .93 (test tissue: .93), respectively, suggesting no evidence of underfitting or overfitting. Similarly, two independent validations also demonstrated stable performance without overfitting and underfitting. The AUC in the plasma cohort did not change significantly and continued to demonstrate a high specificity of 96%. Within the exosome subgroup, the overall training performance improved, with the AUC increasing from .81 to .87. CH group still maintain excellent prediction ability (.96 vs. .97), whereas the test results in noncancer/benign versus cancer group (.82 vs. .79) and benign versus cancer (BC) group (.78 vs. .73) showed a slight decrease in exosome specific models, particularly with the benign group showing lower true negative prediction rate. Based on the performance of tissue and plasma type‐specific models, the AUC values did not change significantly. Moreover, a better balance between bias and variance was achieved, resulting in improved generalisation and stability of the model. However, the observed reduction in specificity within the exosome benign subgroups suggests that a more refined and targeted model is needed to enhance classification precision in this context.

pi‐TPI was also further utilised to predict NSCLC early‐stage (stage IA, IB, IIA and IIB) samples, which are the dominating stages across all stages. Owing to the data information constraints, only tissue samples with detailed stage information were analysed. As illustrated in Figure S11A, pi‐TPI distinguished itself in diagnostic capability across early‐stage samples (IA to IIB). The highest performance was observed in stage IIB with an AUC of .90, sensitivity of 92.0% and a specificity of 75.0% (Figure S11D), followed by stage IB with AUC at .88. For stage IA and IIA, the pi‐TPI achieved AUC of .82 and .83, respectively. When comparing cancer samples from stage I to III with noncancer controls, the PI‐TPI risk score showed statistically significant differences in all pairwise comparisons (Figure S11B and C). Samples with available stages information from independent cohorts also exhibited high early diagnosis value with AUC value at .86 in GSE83527 and .88 in GSE62182 (Figure S11E–H).

The rest of the TCGA tumour samples (*n* = 540) that were not included in training and validation were kept. We randomised this cohort into 12 subsets and paired with 32 normal samples from the TCGA validation cohort to maintain a binary category balance. The 12 validation subsets (T: N = 45:32) were verified by pi‐TPI and the ROC curve illustrated the diagnostic performance by showing AUC values over .9 in each set. The average AUC of 12 total cohorts is .93 (Figure S12).

Other subgroups including sex, smoking history, and subtype were also considered in the pi‐TPI diagnostic capacity verification (Figure S13). Across four distinct cohorts (TCGA, GSE62182, GSE83527, exosome cohort), no significant differences were observed between male and female participants within the normal/tumour groups of each cohort. Similarly, when examining the smoking history subgroups, which included current smokers, former smokers, and never smokers in the GSE62182 and TCGA cohorts, there were no significant differences within each smoking status group. Furthermore, statistical differences in diagnostic performance were detected between LUAD and LUSC with noncancer within the TCGA cohort. Similar results were consistent within exosomal cohorts between noncancer and each NSCLC subtypes. For instance, there was no significant difference between white and black people.

### 2.5 Pearson's correlation analysis of five piRNA signatures as function prediction

**FIGURE 5:**
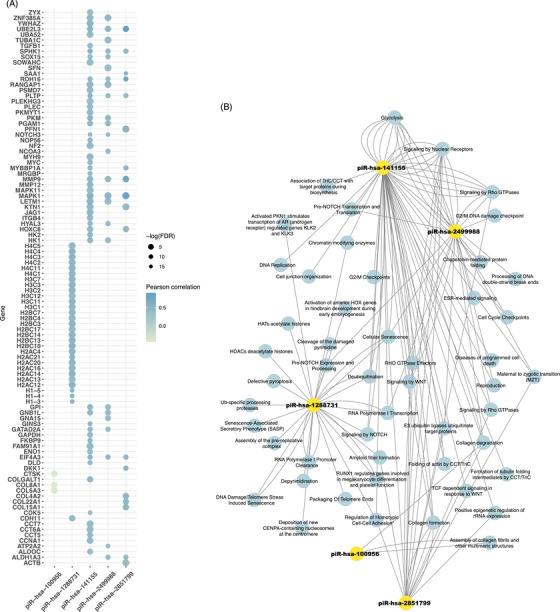
Function prediction of five piRNA based on Pearson's correlation and Reactome. (A) Genes correlated with five piRNA after Pearson's correlation analysis. (B) Network showing the five piRNA with correlated genes that enriched in different signalling pathways.

Pearson's correlation analysis was performed to explore the relationship between piRNA and mRNA expression profiles within the TCGA cohort, utilising the concurrent RNA seq and miRNA seq data available for the same samples. By establishing an absolute correlation coefficient threshold of .3 and a False Discovery Rate (FDR) of less than .05, we identified 457 genes that exhibited correlation with the five piRNA signatures (Table S6). Notably, UBE2L3 demonstrated the most pronounced correlation, with a coefficient of .9 in association with piR‐hsa‐2851799 (Figure 5A). We subjected all correlated genes to Reactome pathway analysis, resulting in 108 pathways being identified as significant, incorporating genes associated with piRNA signatures. Correlated genes were accumulated in cellular senescence (piR‐hsa‐141155), DNA replication (piR‐hsa‐141155), G2/M checkpoints (piR‐hsa‐2499988/piR‐hsa‐1288731), cell junction organisation (piR‐has‐2851799), collagen degradation (piR‐hsa‐100956), DNA damage/Telomere stress‐induced senescence (piR‐hsa‐1288731), reproduction (piR‐hsa‐141155/ piR‐hsa‐2499988), chromatin modifying enzymes (piR‐hsa‐141155/ piR‐hsa‐2499988), deubiquitination (piR‐hsa‐141155), etc. Histone genes such as H4C5, H3C7, and H3C1 constituted the primary set of genes correlated with piR‐hsa‐1288731 and were predominantly enriched in rRNA‐related pathways, including RNA Polymerase I Promoter Opening and SIRT1‐mediated negative regulation of rRNA expression. 51 pathways randomly selected from 108 annotated pathways were shown in Figure 5B and Tables S6 and S7.

## 3 DISCUSSION

The generation of mature piRNAs begin with the long piRNA precursors, which consist of transcripts from piRNA clusters, transposons and mRNAs of gene. These precursors are exported across the nuclear envelop, further processing in the cytoplasm to produce mature piRNAs, marks the importance to track back the origin of piRNA.^23^ In this study, we integrated three different databases for piRNA annotation: piRbase, piRNA cluster database, and Dfam,^32^ an openly accessible repository of Transposable Element DNA sequence alignments (https://www.dfam.org/home). When we reviewed the five piRNA signatures based on their chromosome location in the Dfam, we found that all were identified as originating from the subunit of a ribosome due to its repetitive features arranged in tandem arrays, as annotated in Dfam. piR‐hsa‐100956, piR‐hsa‐1288731, and piR‐hsa‐12851799 are traced to the 28S ribosomal ribonucleic acid (rRNA) from large subunit ribosomal ribonucleic acid (LSU‐rRNA) with the accession number DF000000772 after the BLAST verification. piR‐hsa‐2499988 correlates with the 5S rRNA gene/pseudogene (accession number: DF000000012) and piR‐hsa‐141155 is associated with the small ribosomal ribonucleic acid (SSU‐rRNA) gene/pseudogene (accession number: DF000001066). All the blast result regarding each piRNA with rRNA can be found in Figures S14–S16.

Ribosome is a complex ensemble of rRNA and ribosomal proteins and functions as mRNA translation machine. Ribosomal RNA genes (rDNAs), encoding rRNA, are crucial plays in maintaining genome stability and any disturbance at rDNA loci may have a great impact on cellular processes, such as the response to DNA damage or overall cell longevity.^33^, ^34^ Although we incorporated recorded piRNA cluster information for mapping and annotation, all the five piRNA signatures were identified as not belonging to any piRNA clusters listed from piRNAclusterDB. Instead, their sequences were all recognised as originating from rDNA of LSU (60S) and SSU (40S). LSU (60S) and SSU (40S) are the two subunits of the ribosome, each composed of different rRNAs and proteins and responsible for different aspects of protein synthesis. The 5S rRNA is a component of the LSU and plays a role in the structure and function of the ribosome.^35^ Due to rRNA complex composition, heterogeneity from variabilities including rRNA modifications, rRNA variants, posttranslational modifications, and ribosome‐associated proteins contribute to the ‘specialised ribosome’ or the term ‘onco‐ribosomes’. These onco‐ribosomes in cancer cells play a role in the preferential translation of oncogenic genes and pro‐survival genes that facilitate cancer progression.^36^ For example, recent studies have discovered the linked mutation in ribosomal proteins and aberrant ribosomes with poor prognosis.^36^, ^37^, ^38^, ^39^, ^40^ Additionally, most malignancies have paired 5S rDNA amplification, and 45 rDNA deletions are linked with higher proliferation rates and unregulated expression of nucleolar genes.^36^ From our five signatures, piR‐hsa‐2499988 related to 5S rRNA is identified as downregulated in tumour samples, indicating the association with potential inhibition mechanism of cancer cell proliferation in tissues.

Based on Pearson's correlation analysis and Reactome pathway enrichment results, piR‐hsa‐2499988 was found to be positively correlated with the gene GATA zinc finger domain containing 2A (GATAD2A), a component of the Nucleosome Remodelling and Deacetylase (NuRD) complex involved in chromatin remodelling and histone deacetylation,^41^ and GATAD2A is associated with RNA polymerase I promoter clearance and RNA polymerase I transcription. Interestingly, although piR‐hsa‐2499988 originates from the 5S rRNA region, which is typically transcribed by RNA polymerase III, it was found to be correlated with genes enriched in RNA polymerase I activity. In addition, histone genes such as H2AC16 and H3C11, which were correlated with piR‐hsa‐1288731, another downregulated piRNA signature originating from the 28S rRNA region were also identified. These genes were further enriched in pathways including Histone deacetylases (HDACs) deacetylate histones, RNA Polymerase I Promoter Opening, and B‐WICH complex positively regulates rRNA expression. The correlations seem that these piRNA signatures are linked to regulation of rDNA transcription, mainly through modulating chromatin structure and RNA polymerase I activity at rDNA loci. In addition to rDNA transcription‐related pathways, pathways involved in cell cycle control, such as DNA replication, as well as responses to cellular stress, including DNA damage and cell senescence, were also enriched among the pathways correlated with the piRNA signatures, and these processes are closely linked to carcinogenesis.^42^, ^43^, ^44^ However, the mechanistic basis associating with piRNAs and rRNA regulation, particularly in the context of cancer, remains unclear and requires further investigation. As previously noted, transcripts from piRNA clusters are the primary source of long piRNA precursors. Given the findings from our study, rRNA transcripts may represent a potential source of piRNA precursors and relate carcinogenesis pathways. Nonetheless, the mechanisms underlying this form of piRNA biogenesis remain largely elusive.

Traditional cancer diagnosis involves invasive methods like surgery and biopsy. An ideal clinical diagnosis should be no complicated procedures involved, less pain, low cost and accuracy. Several emerging serologic biomarkers have been applied in clinical use for early lung cancer screening and diagnosis such as carcinoembryonic antigen (CEA) and serum cytokeratin 19 fragments (CYFRA 21‐1), which can be analysed conveniently and economically.^45^ However, the clinical diagnostic potential verification of these biomarkers is not always consistent. For example, some providers don't use CEA for cancer screening but use it for treatment response monitoring. From the review articles of Grunnet et al., CEA levels could not support the diagnostic potential in six studies but carry prognostic information in NSCLC studies.^46^ Another study from Okamura et al. stated that both CEA and CYFRA 21‐1 are reliable serum tumour markers for the diagnosis of lung cancer.^47^ Noninvasive biomarkers such as cfDNA and ctDNA from blood are considered fast and safe for cancer diagnostics. For example, FDA‐approved gene biomarker panels for noninvasive tests exist as companion diagnostics in oncology.^48^ However, these biomarkers are not developed for truly early cancer diagnosis.

Compared to miRNA, piRNA is recognised as more stable and resistant to degradation and oxidation. 3′ terminal 2′‐O‐methylation and 3′‐to‐5 trimming of piRNA is characteristic of piRNA that protects it from being degraded.^49^, ^50^, ^51^ In addition, piRNA has been detected in various body fluids including serum, plasma, and saliva with its concentration significantly changed depending on the disease type and its progression.^52^, ^53^, ^54^, ^55^, ^56^, ^57^, ^58^, ^59^ Our study included samples from tissue, plasma, and plasma extracellular vesicles (30–150 nm), commonly referred to as exosomes,^60^ which was reported to be associated with cancer progression and metastasis.^61^ Compared to cell free RNA from plasma samples, the biological material in the exosome is much more stable due to the stability and safety characteristics from its protective lipid layer structure,^62^, ^63^ which helped preserve the integrity of their RNA contents, including piRNAs. Regarding sample handling, the plasma samples in our study were stored at –80°C for 1–2 years prior to analysis, demonstrating their long‐term stability under standard biobanking conditions. After thawing, exosome purification was typically completed within 3–4 h, followed by total RNA isolation within 1–2 h. The RNA was then stored with RNase inhibitors at –80°C and used or small RNA‐seq library preparation within 1–2 weeks. These procedures reflect practical timelines and preservation strategies used in real‐world clinical and research settings and help ensure minimal RNA degradation during sample processing.

Our research recruited eleven cohorts from North America to Asia and analysed multisource data, from tissues and blood to plasma‐derived exosome. These disparities in both demographics and sample types highlight the necessity of integrating real‐world biological, clinical, and technical heterogeneity in developing diagnostic that are broadly applicable across the multiregion population. Adjusting data distributions within each dataset can help reduce technical heterogeneity across diverse clinical populations. However, despite these efforts, potential inherent variances cannot be eliminated, and the inherent heterogeneity among separate cohorts prevents achieving the level of consistency observed within a single homogeneous cohort or cohorts composed of the same sample type.

According to the test results of each subgroup, every test cohort was associated with its own optimal classification threshold. The variability in threshold values for pi‐TPI across different sample sets reflects potential inconsistencies, likely attributable to technical heterogeneity arising from differences in population characteristics such as region, race, and experimental protocols. However, our studies are benefited by leveraging the large amount of heterogeneity across multiple datasets cohort that each validation results are in concordance with their robust performance, making it ideal candidates for real‐world application. Furthermore, to verify model effectiveness and mitigate sample‐type heterogeneity, we developed customised models for each sample type, resulting in tissue‐specific, plasma‐specific, and exosome‐specific classifiers. Validation within these specific sample sets revealed a better stability. Compared to mixed sample type trained pi‐TPI, sample‐specific models showed a more fitted calibration curve (Figure S10). Thus, the specific model confirmed that the sample‐type heterogeneity could be the culprit of fluctuated threshold and exerts minimal differences on actual diagnosis capacity for pi‐TPI risk assessment compared to the original diverse sample model. The plasma and exosome subgroups that included benign samples exhibited lower diagnostic performance compared to tissue samples and exosome‐based cancer versus noncancer comparisons. This may be due to sample limitations as well as unresolved multicohort data heterogeneity. Nevertheless, this represents a meaningful step in exploring the potential of piRNAs as diagnostic biomarkers for noninvasive detection of nonsmall cell lung cancer. Compared to tissue biopsy, which cannot be performed repeatedly and may impose physical and psychological burdens on patients, noninvasive diagnostics such as blood‐based testing offer greater ease, convenience, and reduced risk.

Our study is subject to several limitations. (1) The lack of longitudinal data may limit the ability to track tumour progression; the role of piRNA signatures in cancer progression remains unclear. Therefore, our current understanding does not extend to whether these signatures could be used to track tumour progression or predict treatment outcomes in NSCLC patients. (2) As this study focuses on NSCLC prediction, follow‐up data may not be available. Therefore, follow‐up data linked to disease progression will be an important focus in future studies. Currently, there is no piRNAs panel‐based prediction model for cancer diagnosis available, thus, it is challenging to realise this application in clinical practices. (3) While it is essential to evaluate conventional clinical serological biomarkers such as CEA and CYFRA21‐1 or radiomics data such as CT scan alongside piRNA signatures for comprehensive analysis, however, due to the limitations in data accessibility, we were not able to procure the expression data of these conventional biomarkers or the information of nodule size as another set of features, as are documented in the these studies.^64^, ^65^ (4) In addition, distinguishing benign from malignant ground glass nodules (GGNs) are frequently encountered in LDCT, although our model demonstrates robust performance in early‐stage NSCLC, a key limitation is the lack of validation using datasets specifically annotated for GGNs cases. Several studies have utilised multiomics, whole‐slide images (WSI), radiomics information and CT to develop prediction model for lung cancer.^66^, ^67^, ^68^ We have collected and reviewed nearly 30 published models, encompassing different types of data from sequencing to image data, as well as serological markers. We evaluated each of the performance and study design (Table S4). Compared to these previous works, our study represents the first NSCLC diagnosis study that integrates multicohorts, independent validation sets, in‐house data as well as benign samples, achieving high diagnosis accuracy.

Low dose computed tomography (LDCT) has been widely utilised for lung cancer screening due to its sensitivity of small lung nodule detection.^69^ Several deep learning‐based models developed for lung cancer screening have reported diagnostic accuracies that exceed those of experienced radiologists in specific settings.^70^, ^71^, ^72^, ^73^ Although the current accuracy of pi‐TPI may not yet match that of CT imaging, blood draws pose no radiation exposure and avoid the long‐term cancer risks associated with repeated CT scans.^74^ In addition, it is important to emphasise that no diagnostic approach, including AI‐based models, can guarantee 100% accuracy. Even interpretations by experienced radiologists are subject to false positives and false negatives, which remain a significant clinical challenge. Our model, based on a panel of piRNA signatures derived from tissue or liquid biopsy samples, is expected to augment diagnostic confidence, particularly in scenarios where imaging results are inconclusive or when invasive procedures are not feasible. By offering an additional diagnostic layer through noninvasive liquid biopsy, the pi‐TPI model has the potential to reduce clinical and financial burdens associated with missed diagnoses, delayed treatment, and unnecessary biopsies. Thus, a multimodal approach integrating longitudinal data, clinical information, histological images, CT scans, and blood tests combining piRNA signature with other small noncoding RNAs such as miRNA or snoRNA may enhance the precision of diagnosis, and we will assess the effectiveness in the future studies. We believe these could pave the way for examining diagnostic factors.

## 4 CONCLUSIONS

Our research has extensively explored the identification of NSCLC using consistent five piRNAs across both tissue samples and noninvasive methods. We applied machine learning techniques to construct the pi‐TPI by leveraging heterogeneity across multicentre and multisource data. This tool has demonstrated strong capabilities in detecting NSCLC across various sample types, as well as distinguishing between noncancerous, benign, and malignant samples. Furthermore, the diagnostic signature's effectiveness was corroborated across different disease stages.

## 5 MATERIALS AND METHODS

### 5.1 Public multicentre patient cohorts

Tissue and plasma data including malignant and nonmalignant samples were collected from several different independent cohorts from diverse regions with noncoding small RNA‐seq, and duplicated samples or samples with low sequencing qualities were removed. We have 1426 tissue samples from TCGA‐LUAD (559/USA), TCGA‐LUSC (523/USA), GSE83527(52/Canada), GSE62182 (56/Canada), GSE175462 (140/Canada), GSE110907 (96/Korea), 192 plasma samples from GSE148861 (49/China), GSE148862 (27/China), GSE204951 (92/Spain), 24 plasma pooling samples and 192 plasma exosome samples from our institute (USA). Since each pooling sample was assembled from ten donors, thus, 240 patients in total were involved in pooling samples. Detailed patient information can be found in the Supplementary File (Table S1). Due to the lack of explicit documentation of race or ethnicity in most cohorts, demographic information related to race was not incorporated into the analyses of every cohort in this study.

### 5.2 Cohort assignment

We collected a total of 1810 tumour, normal, and benign sequencing data from multicentres, including 1426 tissue samples, 192 plasma samples (with 24 pooling in‐house samples), and 192 in‐house exosome samples, culminating in a total of 2050 samples. As for the disproportionate tumour‐to‐normal ratio from TCGA‐LUAD and TCGA‐LUSC cohorts, we selectively included 182 matched pairs of tumour and normal samples, and we incorporated 40% of the remaining tumour samples. This subset was subsequently merged with further datasets (tissue: GSE175462, GSE110907; plasma: GSE148861, GSE148862, GSE204951, RUSH_pooling, exosome: CHTN) to form the final cohort, ensuring a balanced distribution of tumour and normal samples. The final cohort comprises 1162 samples, with a tumour‐to‐normal ratio of 1.75:1. Our dataset was randomised into two distinct groups: 70% (812 samples) were designated for the training subsample to construct the prediction algorithm, while the remaining 30% (350 samples) constituted the holdout validation subsample, utilised to evaluate the trained classifier. Other two GEO datasets (GSE62182 and GSE83527) containing tissue samples were used as independent validations for model evaluation, which will not be involved into the training step.

### 5.3 Signature discovery in multicentre and multisource cohorts

To identify NSCLC‐associated piRNAs, we analysed over 1 million piRNA expression profiles from cancer and noncancer samples including tissue, plasma, exosome, from paired TCGA‐LUAD, TCGA‐LUSC, GEO cohorts, and exosome CHTN cohorts. We filtered out piRNAs with low counts, for which unexpressed in over 50% of the samples, and identified 2993 piRNAs present across all included cohorts. To refine the pool of candidate variables and identify piRNA signatures with consistent expression trends across all cohorts, we calculated differentially expressed piRNAs (DEpiRNAs) by DESeq2 and retained only those exhibiting a consistent direction of fold change.

### 5.4 Random forest model training procedures

Random forest classifier was implemented by using the scikit‐learn package in Python (3.83) programming language. The initial feature selection from training set was based on random forest recursive feature elimination algorithm with fivefold cross validation using Recursive Feature Elimination‐Random Forest (RF‐RFE) to shrink the number of variables. The score was calculated by evaluate_model function, and the mean score from each resampling is .7942 ± .0488 (cut off: .5). To develop an optimal random forest classifier, the hyperparameter optimisation technique GridSearchCV function with fivefold cross‐validation were applied to the training set. Model features were used as the 13 piRNAs screened out from initial selection. A total of 9000 random forest models were evaluated with different combination of hyperparameters: n_estimators = [50, 100, 200, 300], boostrap = [True,False], max_depth = [2, 3, 4, 5, None], max_features = [‘sqirt’, 2, 3, 4, 5], min_samples_leaf = [1, 2, 3], min_samples_split = [2, 3, 4]. The best model was selected by n_estimators = 50, bootstrap = True, max_depth = None, max_features = sqrt, min_samples_leaf = 3, min_samples_split = 2, which achieved the average accuracy is .79 as the highest. Independent validation was conducted using data from GSE83527 and GSE62182 tissue cohorts. The default cutoff is .5. The prediction of outcome would be sample identified as ‘cancer’ or ‘noncancer’. In addition to RFE‐Random Forest, other feature selection methods, including Elastic Net, LASSO‐CV, and SVM‐RFE, were also applied to identify the optimal signature panels. The diagnostic performance of each method's final model is summarised in Table S3.

### 5.5 Logistic regression analysis for piRNA‐based diagnostic signature model and its risk score function

To construct a piRNA‐based model capacity of estimating the probabilities of NSCLC onset with clinical applicability, we initially applied Pearson's correlation to reduce collinearity among the 13 piRNAs. Subsequently, five piRNA signatures was developed and utilised to evaluate the risk probability of NSCLC using a prediction function from logistic regression model on the training dataset. Then both the holdout validation set and independent validation sets were tested. The test results interpretation from the model were generated by normalised linear score between 0 and 1, where the minimum and maximum of the linear scores are used to scale the raw logistic regression output to this range:

$$\begin{array}{c} \mathrm{Each}\mathrm{risk}\;\mathrm{score}=\\ \;a_{0}+\sum_{i=1}^{m}(\mathrm{piRNAi}\;\mathrm{coefficient}\;\mathrm{of}\;\mathrm{training}\;\mathrm{logistic}\\ \;\mathrm{regression}\;\mathrm{model}\times \mathrm{piRNAi}\;\mathrm{expression}\;\mathrm{of}\;\mathrm{test}\;\mathrm{cohort}). \end{array}$$

$$\begin{array}{c} \mathrm{piRNA}\mathrm{Base}\mathrm{Tumor}\mathrm{Probability}\mathrm{Index}\;(\mathrm{pi}-\mathrm{TPI})=\\ \;(\mathrm{Each}\;\mathrm{risk}\;\mathrm{score}-\min(\mathrm{risk}\;\mathrm{score}))/\\ \;\left(\max\left(\mathrm{risk}\;\mathrm{score}\right)-\min\left(\mathrm{risk}\;\mathrm{score}\right)\right). \end{array}$$

In this model, *a*_0_ represents the intercept from the logistic regression model, and *m* is the total number of piRNAs included in the model. The prediction of outcome would be sample identified as ‘cancer’ or ‘noncancer’. pi‐TPI was also used to evaluate other clinical and demographic variable such as sex, race, smoking status, and NSCLC stages and subtypes.

### 5.6 High‐throughput data processing and customised annotation file generating

All noncoding small RNA‐seq raw data were processed by Fastp for adapter trimming. miRNA‐seq reads were aligned to human genome hg38 with STAR‐2.7.8. Reads of piRNA features were counted by featureCounts. Customised GTF files for piRNA annotation were generated by the combination of bed file and Fasta file from two piRNA databases: piRbase 3.0 (https://bigdata.ibp.ac.cn/piRBase/) and piRNA cluster database (https://www.smallrnagroup.uni‐mainz.de/piRNAclusterDB/).

piRbase is currently the biggest and most comprehensive piRNA database that collected piRNAs from published literature, Supplementary Files and related GEOs.^75^ It has covered more than 44 species from over 440 datasets, and over eight million of unique piRNAs from human have been included. It also contains most active piRNA in their interaction with PIWI protein referred as gold standard set of piRNAs based on the support from different kinds of piRNA enrichment datasets. These datasets consist of matched PIWI protein immunoprecipitation (IP) or oxidation treatment versus small RNA‐seq. In order to specifically identify the piRNAs derived from the piRNA clusters, all possible piRNAs from piRbase that fall in the genomic coordinates of piRNA cluster were retrieved. And rest of piRNAs belonging to the gold standard set that don't fall in the piRNA cluster range were also included. Finally, the GTF annotation file consists of 1835457 piRNAs.

### 5.7 piRNA expression mining and ML model development

The two normalisation techniques, transcripts per million (TPM) and the DESeq2 method coupled with variance stabilising transformation, have been utilised to transform raw count data into a normalised matrix suitable for subsequent analysis. ‘limma’ package and removeBatchEffect() function was used on TPM normalised data to adjust distribution and correct the batch effect in each cohort caused by nonbiotechnological bias, and the prcomp function was used to check the clusters of each independent dataset. All differentially expressed piRNAs between noncancerous and cancer groups were calculated by ‘DESeq2’ package. The training set, consisting of 70% of the data, and the validation set, accounting for the remaining 30%, were composed by 182 paired samples from the TCGA‐LUAD and TCGA‐LUSC cohorts, along with 40% of rest cancer samples from TCGA, and samples from GSE175462 and GSE110907 within the GEO tissue cohort. Data from plasma cohorts GSE148861, GSE148862, GSE204951, pooling plasma from RUSH and exosome cohort data from CHTN, were also included, culminating in 2050 samples.

### 5.8 ROC and precision–recall curve analyses

The AUC and AUPRC values from random forest and logistic regression models were generated by roc_curve function. The optimal thresholds of the probabilities of the diagnosis computed by the Random Forest and Logistic Regression models, which can discriminate cancer and noncancer. The optimal thresholds for ROC curve were determined by the Youden's index and the highest F‐score from each validation set.

### 5.9 Model performance evaluation metrics

Confusion matrices was used to evaluate the performance of the diagnostic model. The basic components of the table including true positives (TP): model correctly predicts the positive class; true negatives (TN): model correctly predicts the negative class; false positives (FP): model incorrectly predicts the positive class; false negatives (FN): model incorrectly predicts the negative class. Sensitivity/recall is calculated by TP/(TP+FN), which can assess the true positive rate and specificity is calculated by TN/(TN+FP), which can assess the true negative rate. Precision is calculated by TP/(TP+FP), which can demonstrate how accurate the positive predictions are. *F*1 score is calculated by 2TP/(2TP+FP+FN) which is a weighted average of precision and recall.

### 5.10 Correlations and functional enrichment

We computed positive and negative piRNA‐mRNA Pearson's correlation coefficient and Pearson's correlation coefficient using only tumour datasets in TCGA for TCGA‐LUAD and TCGA‐LUSC cohorts. Pearson's correlation > |.3| and FDR < .05 were the selection standards. Correlated genes log2 fold changes were calculated. ‘org.hs.eg.db’ was used as an annotation to carry out enrichment analysis of the Reactome Pathway database in the gene set.

### 5.11 Visualisation

The figures were created utilising ‘Matplotlib’ in Python, ‘ggplot2’, and ‘circlize’^76^ in R, version 4.1.3, supplemented by Bioconductor.

### 5.12 Plasma sample collection

Plasma was obtained from the Rush University and from the CHTN. Adenocarcinoma subjects were grouped into five pools to make all pools statistically identical. Each pool involved 10 white subjects with early‐stage cancers ranging from 1a to 2a, including two males and eight females. The average age of subjects in each group was 70.2 years old, and the average tumour size was 19.4 mm. Kruskal–Wallis test did not show any statistical differences between pools. We prepared 6 pooled plasma samples for LUAD, 6 pooled plasma samples for LUSC, 6 pooled plasma samples for healthy controls, and 6 pooled plasma samples for benign samples.

### 5.13 RNA extraction from plasma

We used miRNeasy Serum/Plasma Kit (QIAGEN) for RNA extraction from plasma following the manufacturer's protocol. Plasma was mixed with Acid‐Phenol/Guanidine‐based lysis buffer to denature protein complexes. After adding chloroform, total RNAs were purified by centrifuging. The aqueous phase contained total RNAs were applied to the RNeasy MinElute spin column to wash away phenol and other contaminants. High‐quality RNAs were then eluted by RNase‐free water. We took 50 µL plasma from each subject, resulting in 500 µL from 10 subjects per pool. Due to the capacity of the kit, we treated 250 µL plasma from one pool at a time and combined two products at the step of MinElute spin column. After eluting with 14 µL RNase‐free water, we added 1 µL of RNase inhibitor.

### 5.14 Exosome purification from plasma

Exosomes were purified from 500 µL of plasma by using Capturem™ Extracellular Vesicle Isolation Kit (Cat. No. 635741, Takara Bio USA, Inc.). Exosomes were eluted with 200 µL elution buffer. Total RNA including small RNA was extracted from these exosome samples using the miRNeasy Serum/Plasma Kit (Cat. No.217184, Qiagen, Germany) following the manufacture protocol.

### 5.15 Small RNA seq

Library prep and exosomal small RNA seq were performed by the Genomics and Bioinformatics Shared Resources (GBSR) at the University of Hawaii Cancer Center. QIAseq miRNA Library Kit and QIAseq miRNA NGS 12 Index IL from QIAGEN were used for making the library, and NextSeq 500 from Illumina was used for sequencing to obtain 10 M reads/sample.

## AUTHOR CONTRIBUTIONS

Z.G. and Y.D. designed the manuscript. Z.G. did the data analysis, visualisation and drafted the manuscript. G. D., AA. A., AJ. H., JA.B., and CW.S. conceived the in‐house plasma data. M.N. conducted experiments. D.K., Z.F., Y.C. T.G., H.Y., and F.Y. revised the manuscript. L.W. and Y.D. supervised the manuscript.

## CONFLICT OF INTEREST STATEMENT

Jeffrey A Borgia is on the scientific advisory board for the Luminex Corporation and Rational Vaccines, Inc. and gets paid as a consultant with both.

## ETHICS STATEMENT

The use of patient data and human blood samples was approved by the University of Hawaii Human Studies Program. The protocol number is 2018‐00636. Protocol title is ‘Profiling genome‐wide circulating ncRNAs for the early detection of lung cancer’. The University of Hawaii (UH) Human Studies Program approved this study as exempt from federal regulations pertaining to the protection of human research participants. The authority for the exemption application to this study is documented in the Code of Federal Regulations at 45 CFR 46.101(b) 4. Since the sample collection was done by CHTN, the individual informed consent was obtained directly by the CHTN in accordance with their institutional protocols.

## Supporting information



Supporting Information

Supporting Information

## Data Availability

The pi‐TPI data and code generated in this study are available on GitHub (https://github.com/rarukua/pi‐TPI).
