# Supplementary material for: A robust machine learning model based on ribosomal‐subunit‐derived piRNAs for diagnostic potential of nonsmall cell lung cancer across multicentre, large‐scale of sequencing data
Source: Clin Transl Med. 2025 Jul 25;15(8):e70418. doi: 10.1002/ctm2.70418 (PMC12410371; doi:10.1002/ctm2.70418)
Supplement: Supplementary file 1 — Supporting Information [file CTM2-15-e70418-s001.pdf]

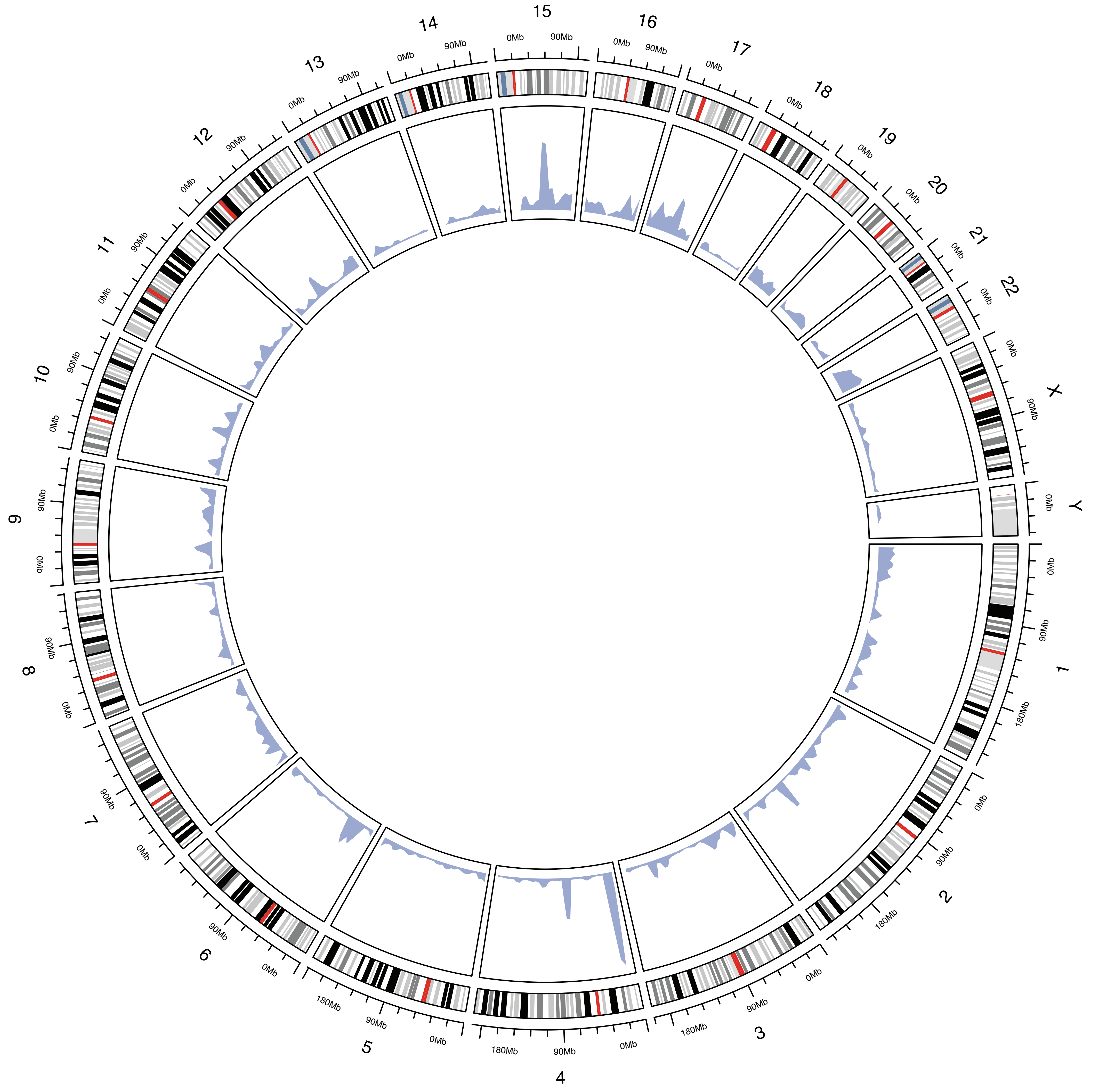

Supplementary Fig 1. All annotated piRNA distribtion and density across all chromosomes including Y chromosome.

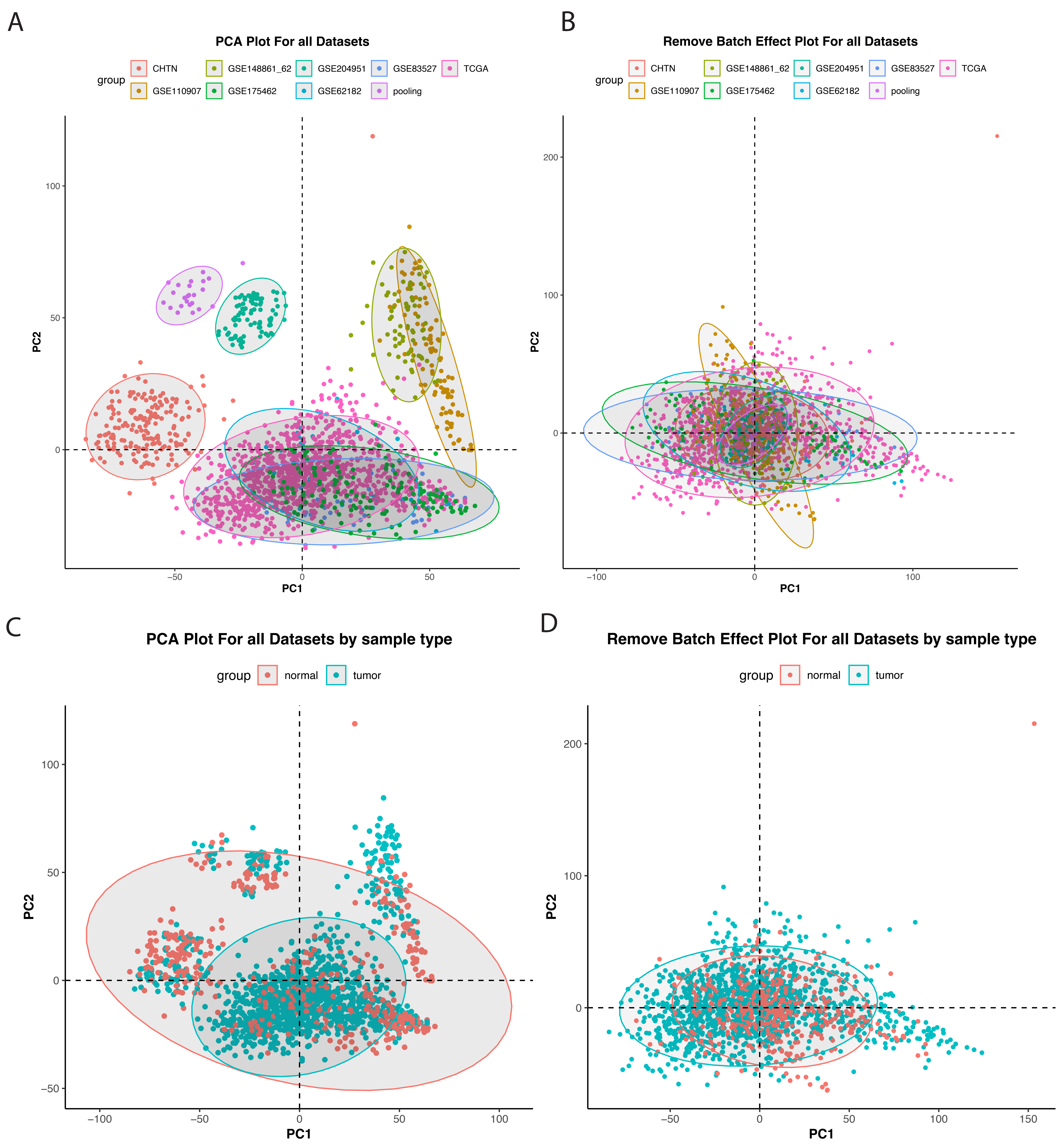

Supplementary Fig 2. Visualized result from principle component analysis (PCA) of 11 cohorts. (GSE148861 and GSE148862, TCGA-LUAD and TCGA-LUSC were labeled as GSE148861_62, and TCGA, respectively). (A): before removing the bacth effect; (B): after removing the batch effect; (C): PCA plot categoried by sample type before removing the batch effect; (D): PCA plot categoried by sample type after removing batch effect.

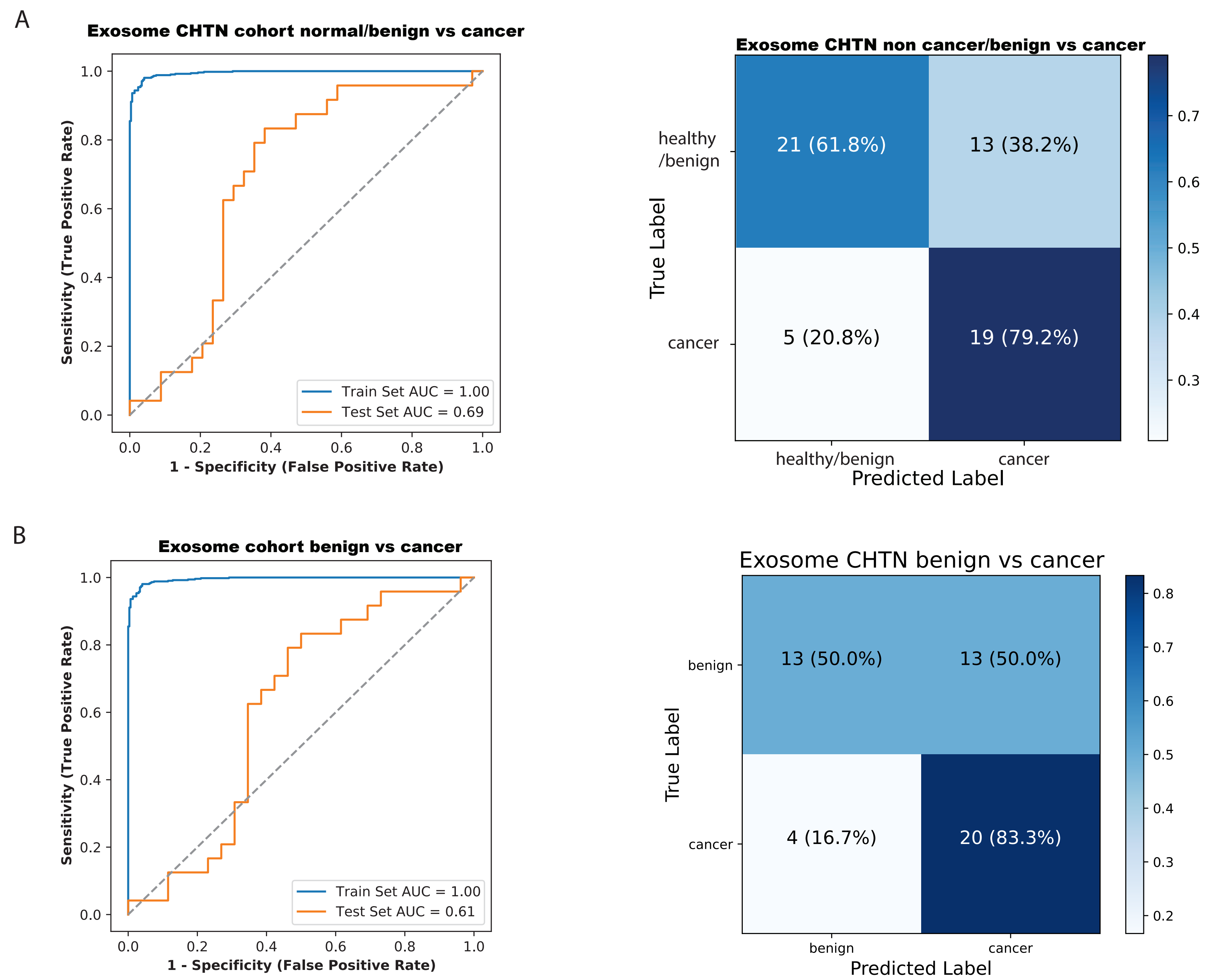

Supplementary Fig 3. ROC curve and confusion matrices in two subgroups from exosome cohort.

(A): ROC curve showing the prediction performance of 13 piRNA RF model in exosome subgroup including healthy/benign and tumor samples as comparsion(left); confunsion matrices showing the tumor/non-tumor outcomes predicted by 13 piRNA RF model under optimal threshold(right).

(B): ROC curve showing the prediction performance of 13 piRNA RF model in exosome subgroup including benign and tumor samples as comparsion(left); confunsion matrices showing the tumor/non-tumor outcomes predicted by 13 piRNA RF model under optimal threshold(right).

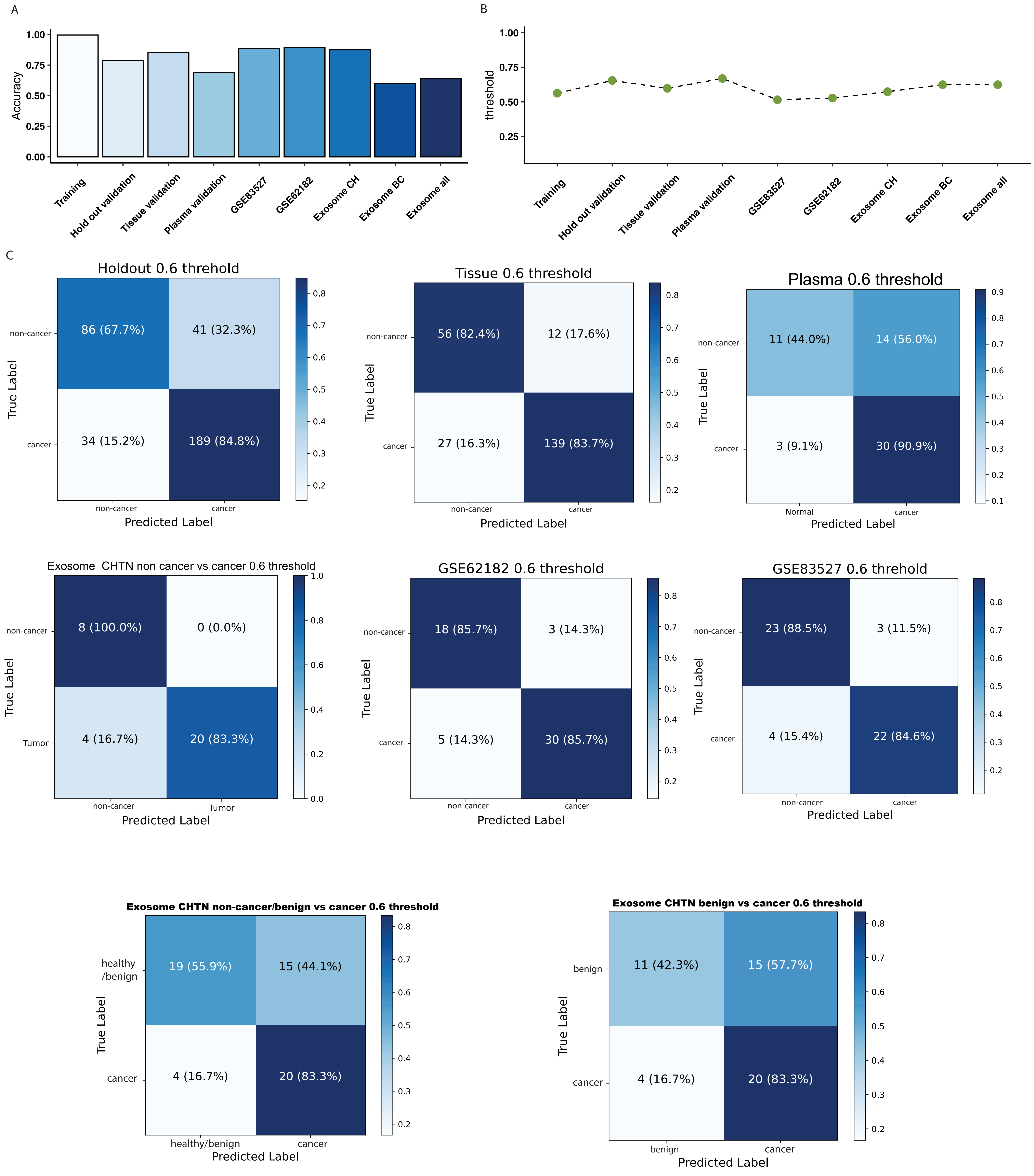

Supplementary Fig 4. (A): Accuracy using defulat cutoff 0.5 from 13 piRNAs RF model across each cohort [Training, Holdout, Tissue, Plasma, GS83527, GSE62182, Exosome];
(B): Optimal threshold of each cohort from 13 piRNAs RF model based on the ROC curve;
(C): Confusion matrices showing tumor predictions using 13 piRNAs RF model under the cutoff 0.6 based on the average optimal threshold from each cohort.

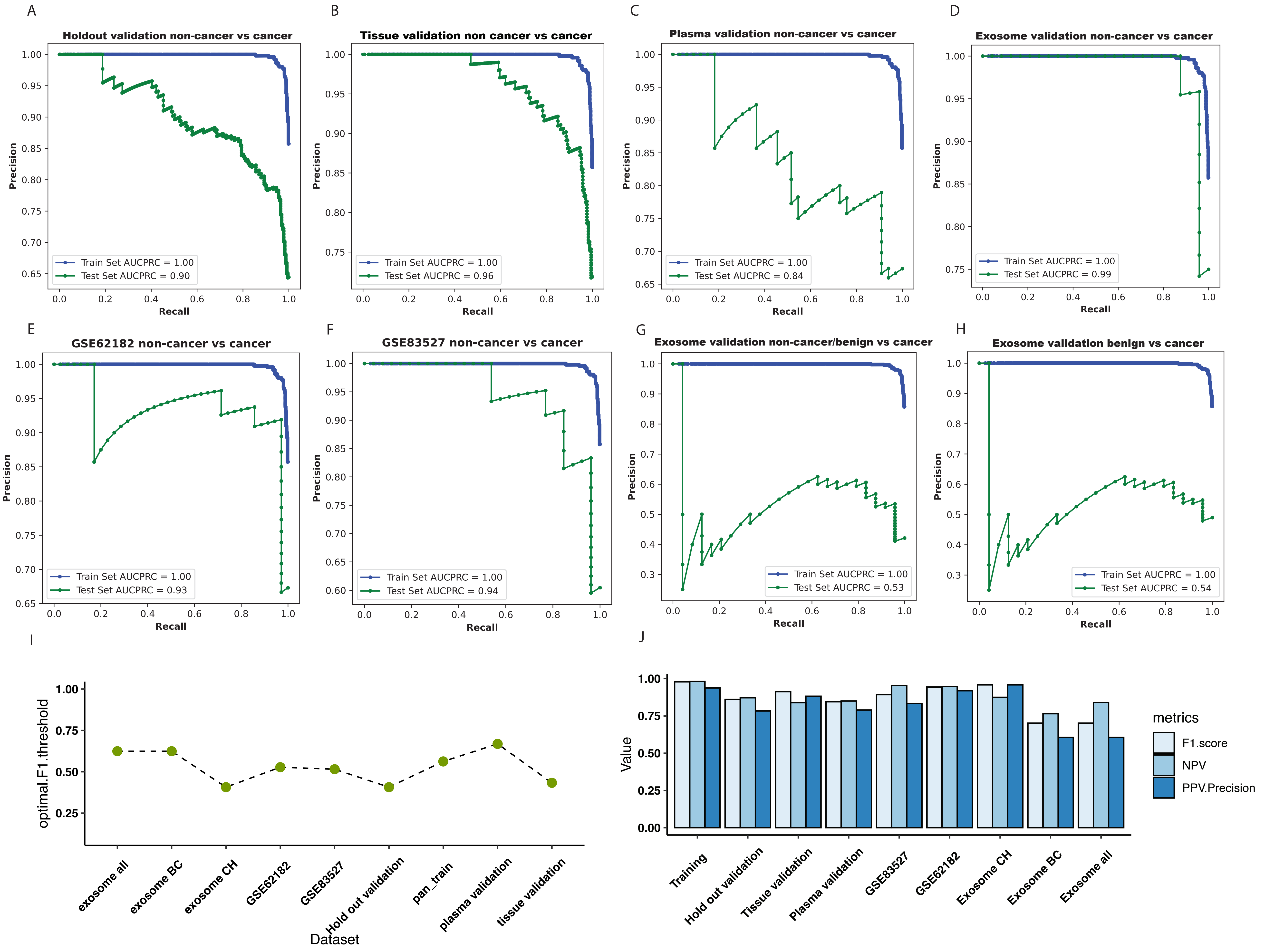

Supplementary Fig 5. 13 piRNAs RF model performance illustrated by the precision-recall curve across multiple sample types in the test set. The corresponding AUPRC values of 13 piRNAs RF model in holdout cohort, tissue cohort, plasma cohort, indenpendent cohorts and exosome cohorts with training cohort are shown in Fig S4 A-H. The optimal F1 threshold in each cohort were shown in (I). (J): F1 score, negative predictive value (NPV), positive predicitive value (PPV) from the performamce measurement of the model in each cohort are shown in the barplot.

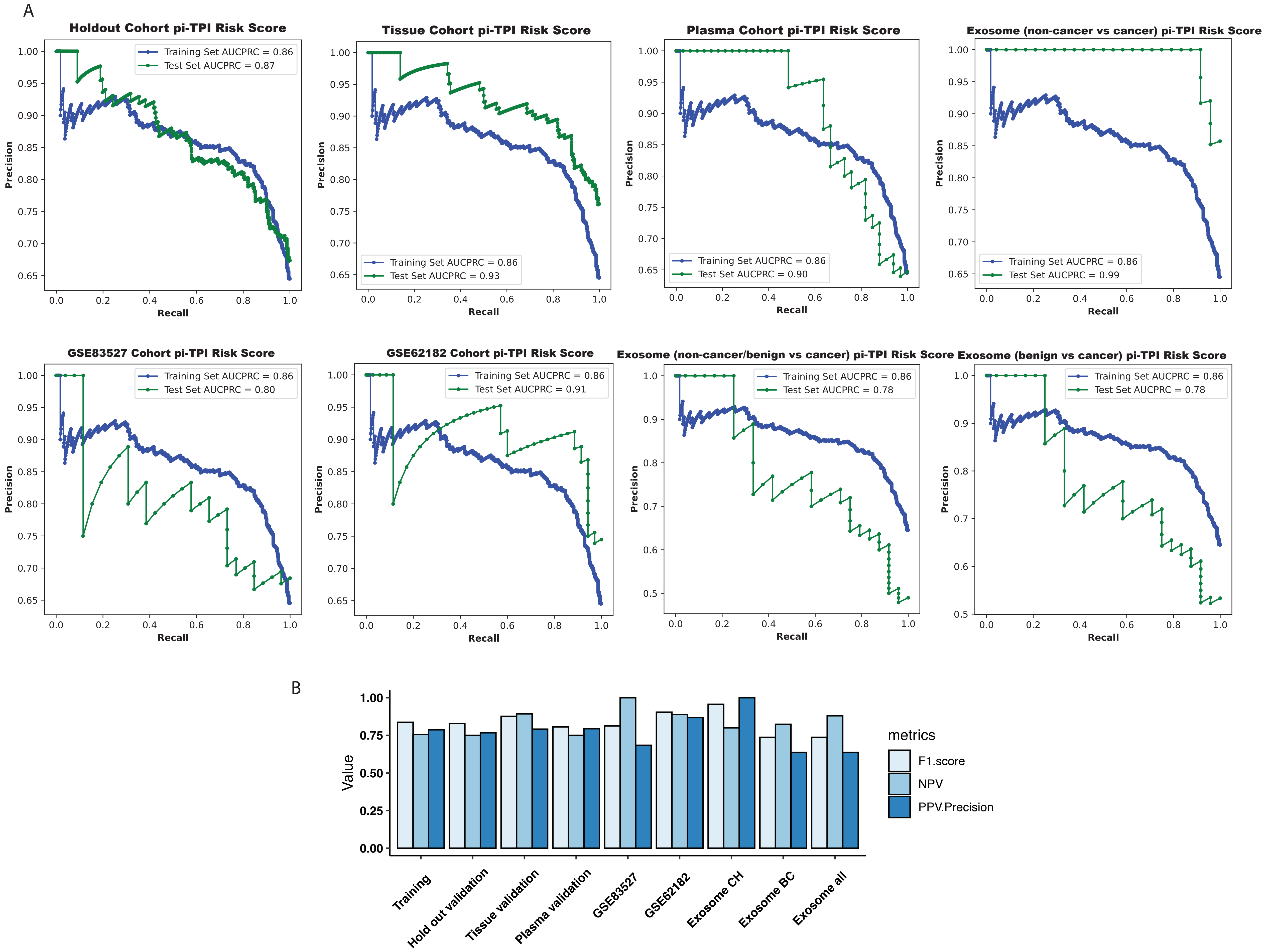

Supplementary Fig 6. (A): pi-TPI Risk Probabilities performance illustrated by the precision-recall curve across all cohorts in the test set. The corresonding AUPRC values of pi-TPI are shown in each test set. (B): F1 score, negative predictive value (NPV), positive predicitive value (PPV) from the performamce measurement of pi-TPI risk probabilities in each cohort are shown in the bar plot.

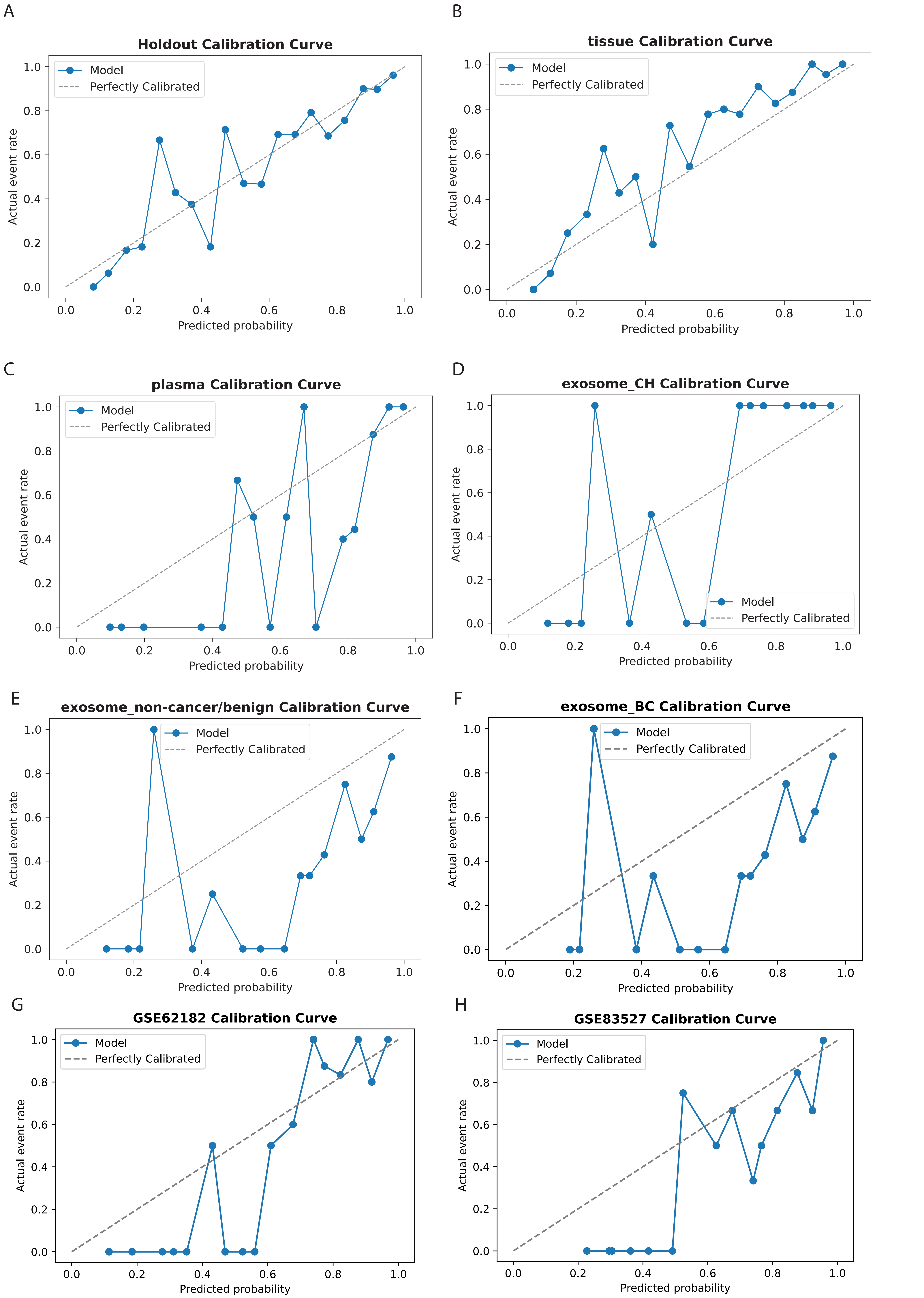

Supplementary Fig 7. Calibration curve of pi-TPI Model

A-H: each curve demonstrated the alignment between the predicted probability and the actual event rate in each cohort. Most curves can provide a good alignment between predicted and observed canse in high probility ranges.

Tissue specific model for pi-TPI risk score

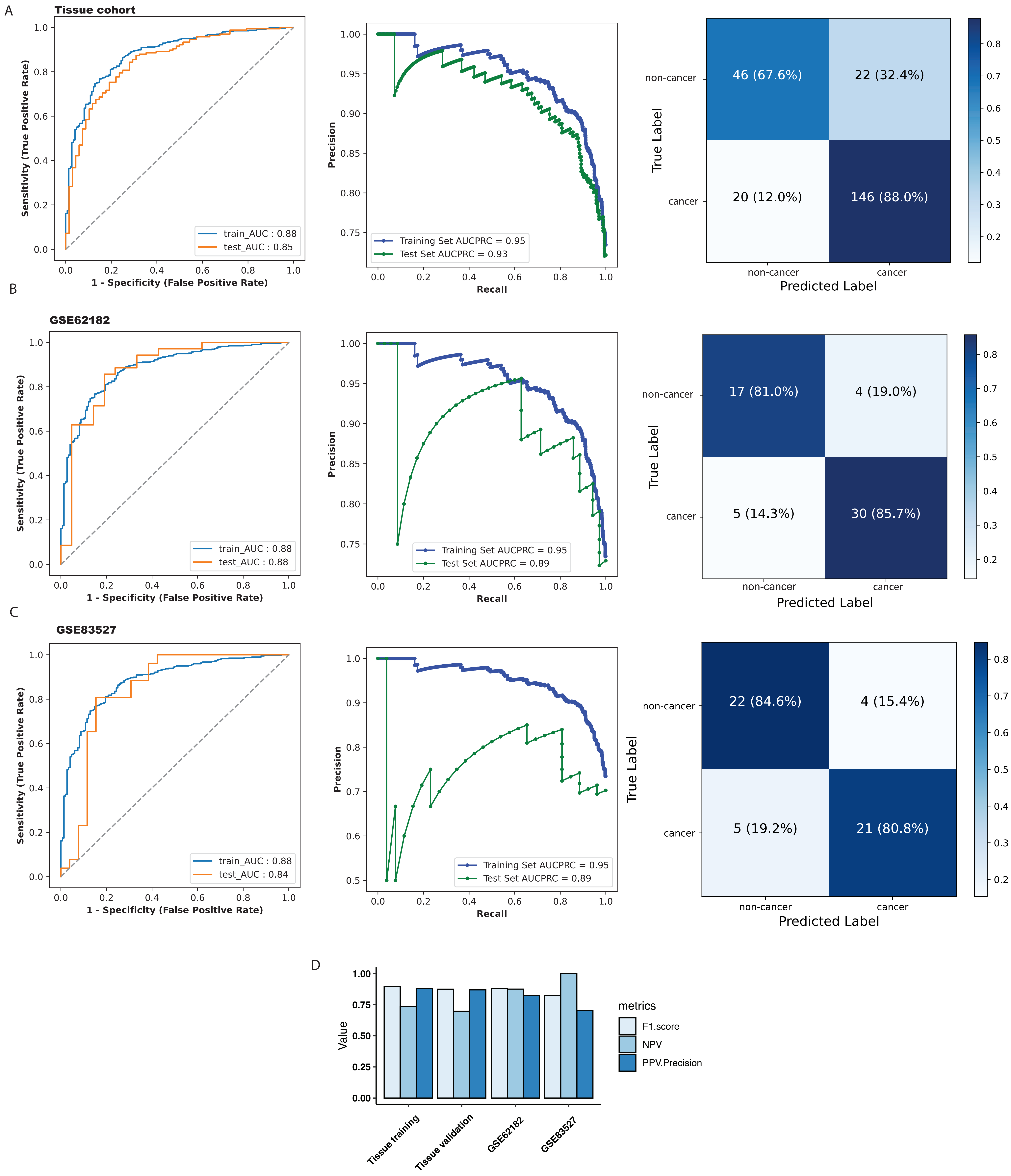

Supplementary Fig 8. Model performance of Tissue specific model based on pi-TPI risk probabilities.
(A-C): ROC curves with AUC values showing the pi-TPI performance of tumor prediction ability in both training and test set. Precision-recall curves with AUPRC values showing the tumor cases prediction ability of pi-TPI in both training and test set. Confusion matrices showing optimal tumor outcome predicted by pi-TPI in all tissue-related cohorts. (D): F1 score, negative predictive value (NPV), positive predicitive value (PPV) from the performamce measurement of pi-TPI risk probabilities in each cohort are shown in the bar plot in tissue training, validation and two independent validations.

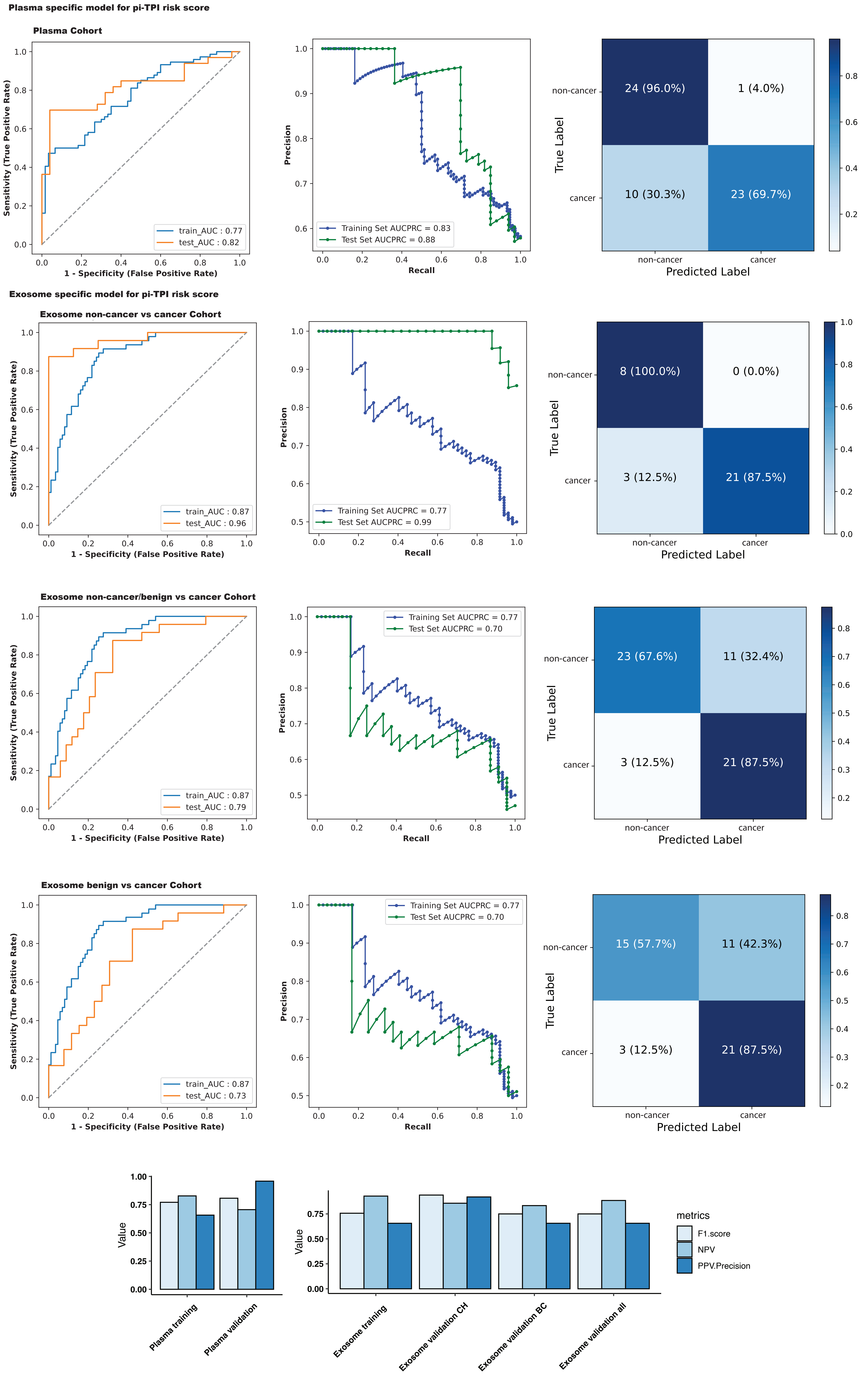

Supplementary Fig 9. Model performance of Plasma and exosome specific model based on pi-TPI risk probabilities. (A-C): ROC curves with AUC values showing the pi-TPI performance of tumor prediction ability in both training and test set. Precision-recall curves with AUPRC values showing the tumor cases prediction ability of pi-TPI in both training and test set. Confusion matrices showing optimal tumor outcome predicted by pi-TPI in all blood-related cohorts. (D): F1 score, negative predictive value (NPV), positive predicitive value (PPV) from the performamce measurement of pi-TPI risk probabilities in each cohort are shown in the bar plot in plasma and exosome training, validation and two subgroup validations within exosome samples.

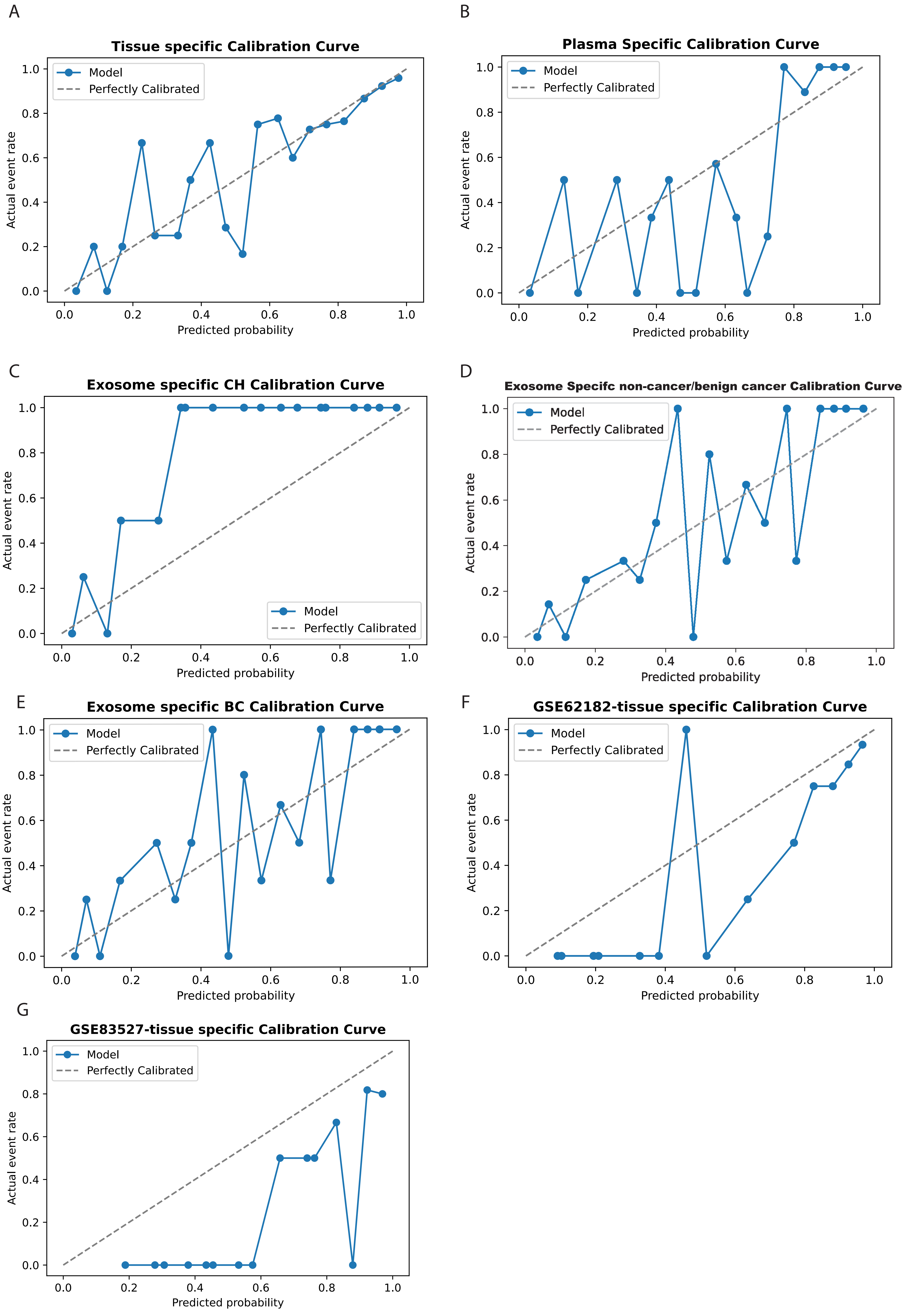

Supplementary Fig 10. Calibration curve of specific pi-TPI Model

A-G: each curve demonstrated the alignment between the predicted probability and the actual event rate from sample type specific models, including tissue-specific model, plasma specific model, and exosome specific model.

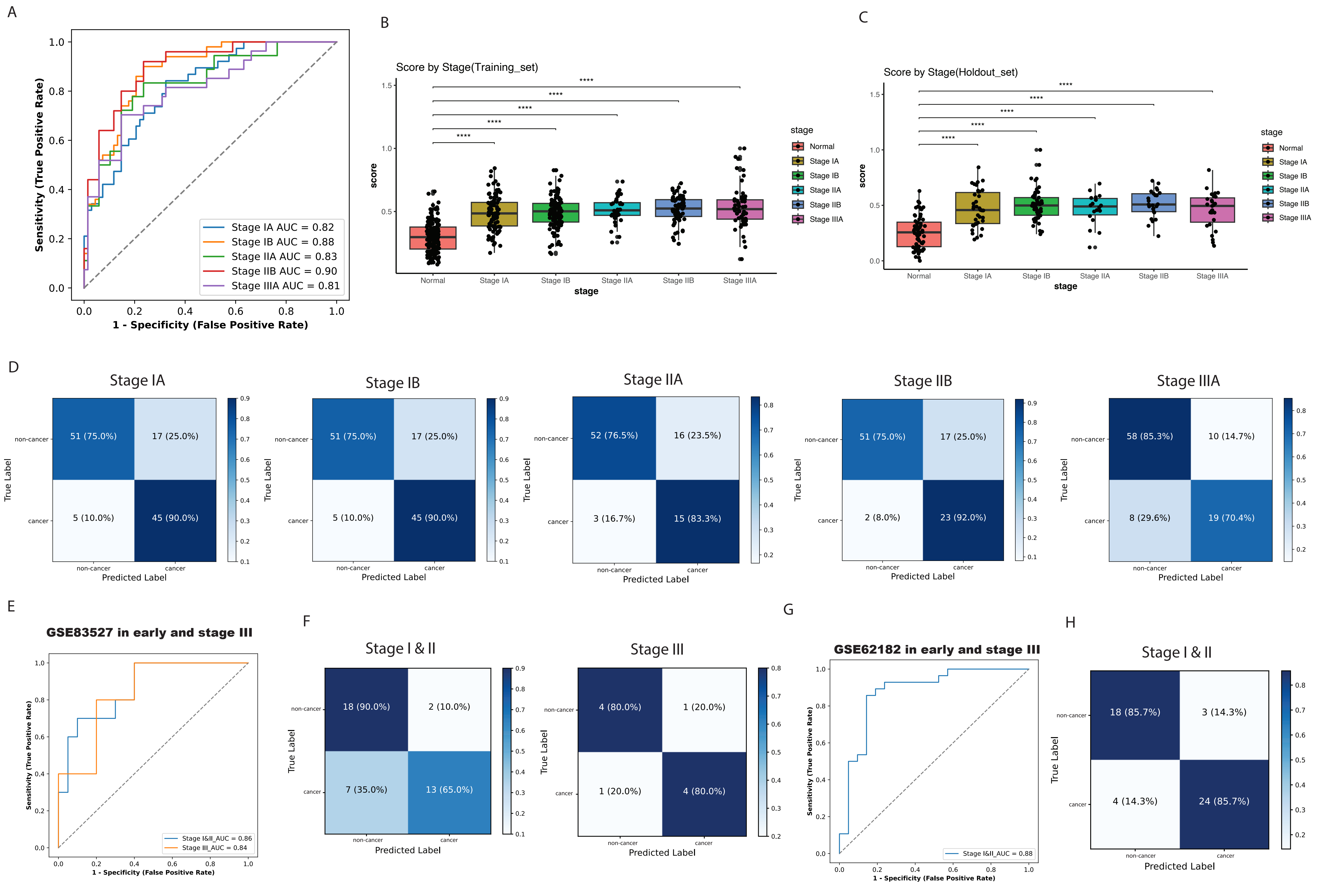

Supplementary Fig 11. pi-TPI validation in tissue samples across clinical stages (Stage I to Stage III).
(A): AUC curve for pi-TPI across tissue samples from stage IA, IB, IIA, IIB and IIIA
(B): PI-TPI risk score for each stage in training set.
(C): pi-TPI risk score for each stage in test set.
(D): Confusion matrices showing optimal tumor prediction outcome in stage IA, IB, IIA, IIB, IIIA across all tissue samples in test set.
(E-F): AUC curve and confusion matrices showing optimal tumor prediction outcome in early stages and stage III from dataset GSE83527.
(G-H): AUC curve and confusion matrices showing optimal tumor prediction outcome in early stages from dataset GSE62182.

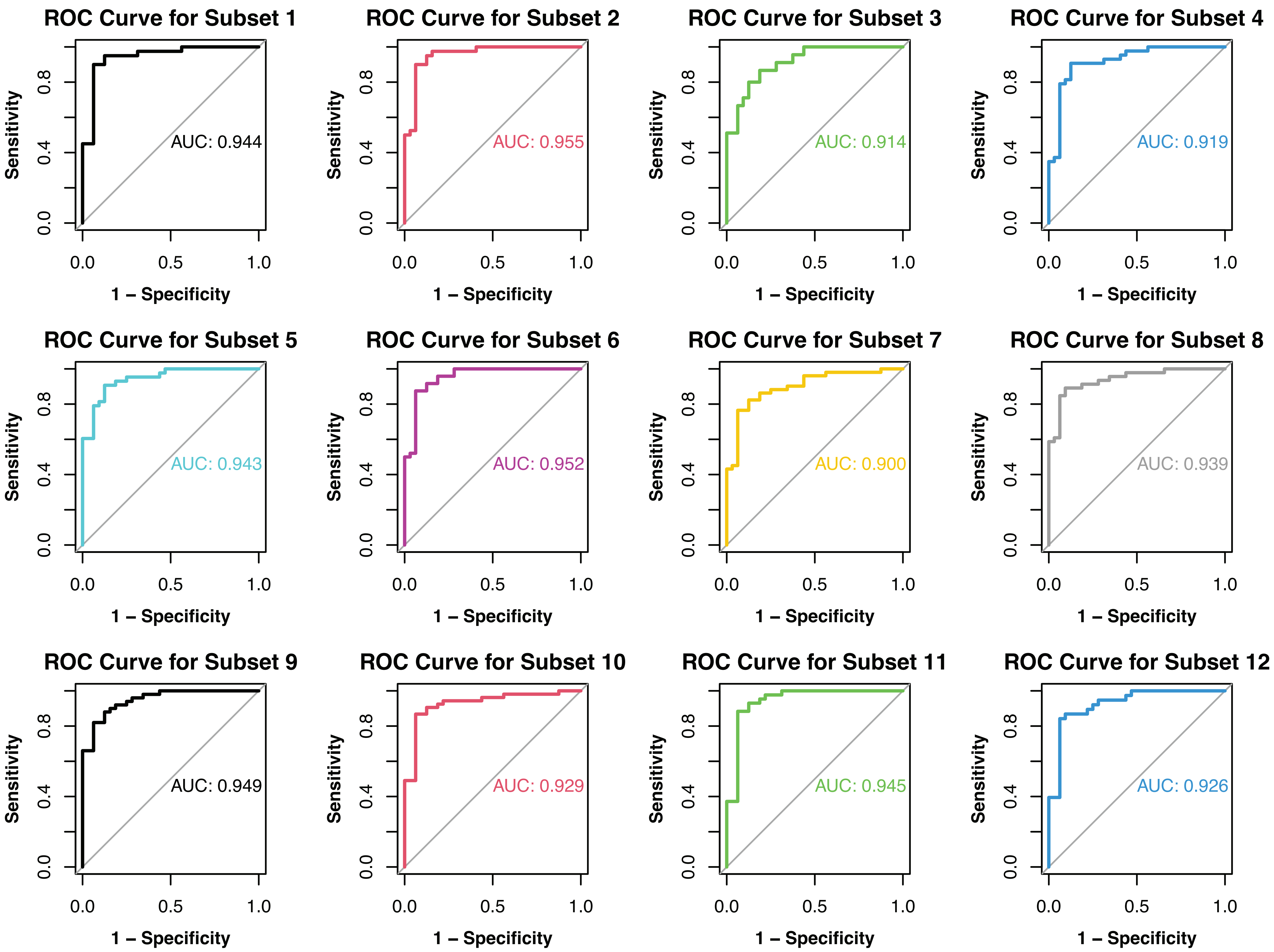

Supplementary Fig 12. 572 TCGA sample verification of pi-TPI.

540 TCGA cancer samples that were not included into holdout validation were split randomly into12 subset datasets, and each subset would pair with 32 TCGA non-cancer samples from holdout validation cohort. ROC curve was utilized to evaluate the each subset validation performace based on pi-TPI risk probabilities.

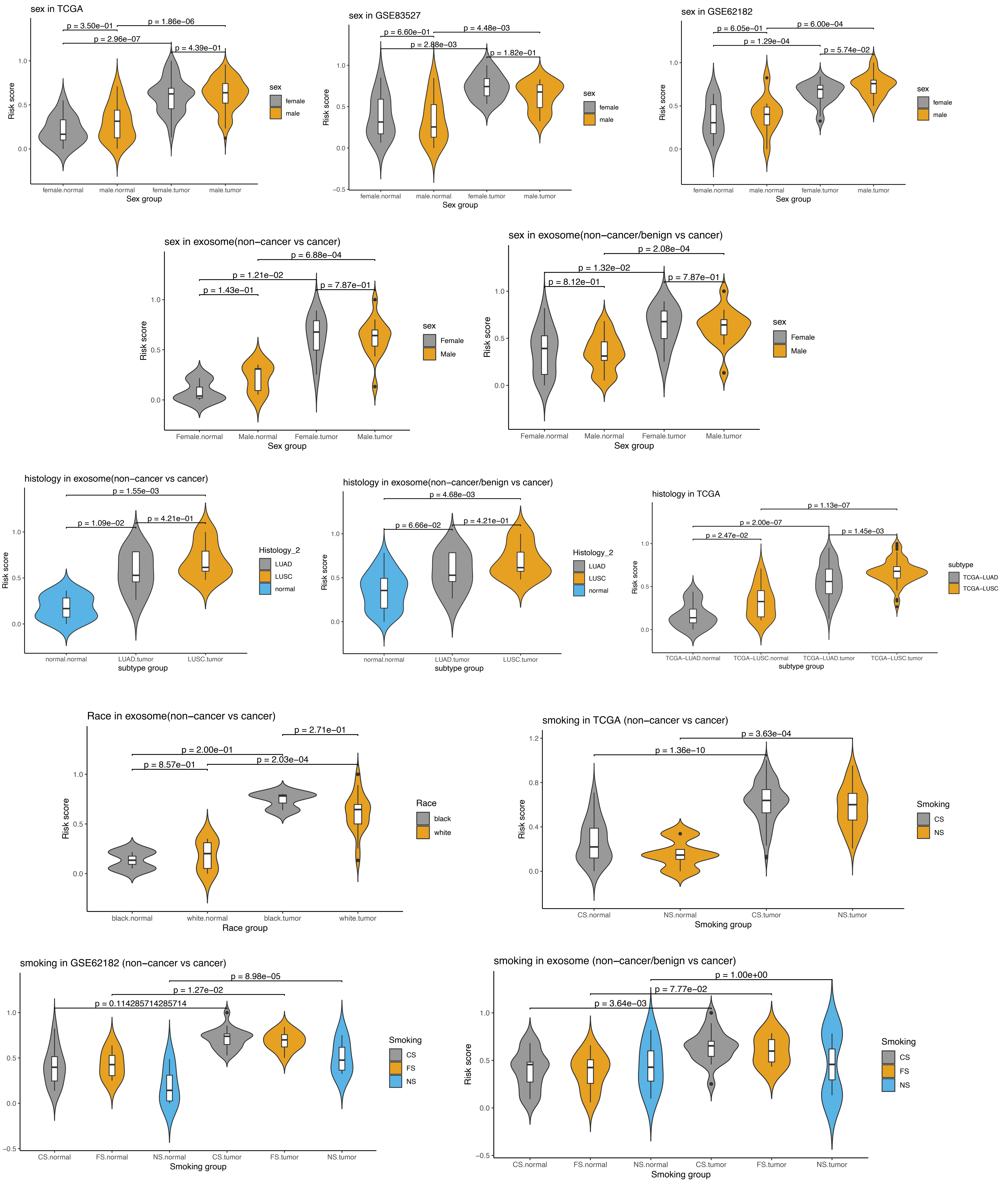

Supplementary Fig 13. Boxplots comparing the pi-TPI predicted risk probabilities between the two groups (non-cancer vs cancer or non-cancer/benign vs cancer) with respect to sex, smoking, cancer histology and race differences in different cohorts. For the boxplot, the center line indicates the median; box limits indicate the first and third quartiles. (CS: current smoke, FS: former smoke, NS: never smoke)

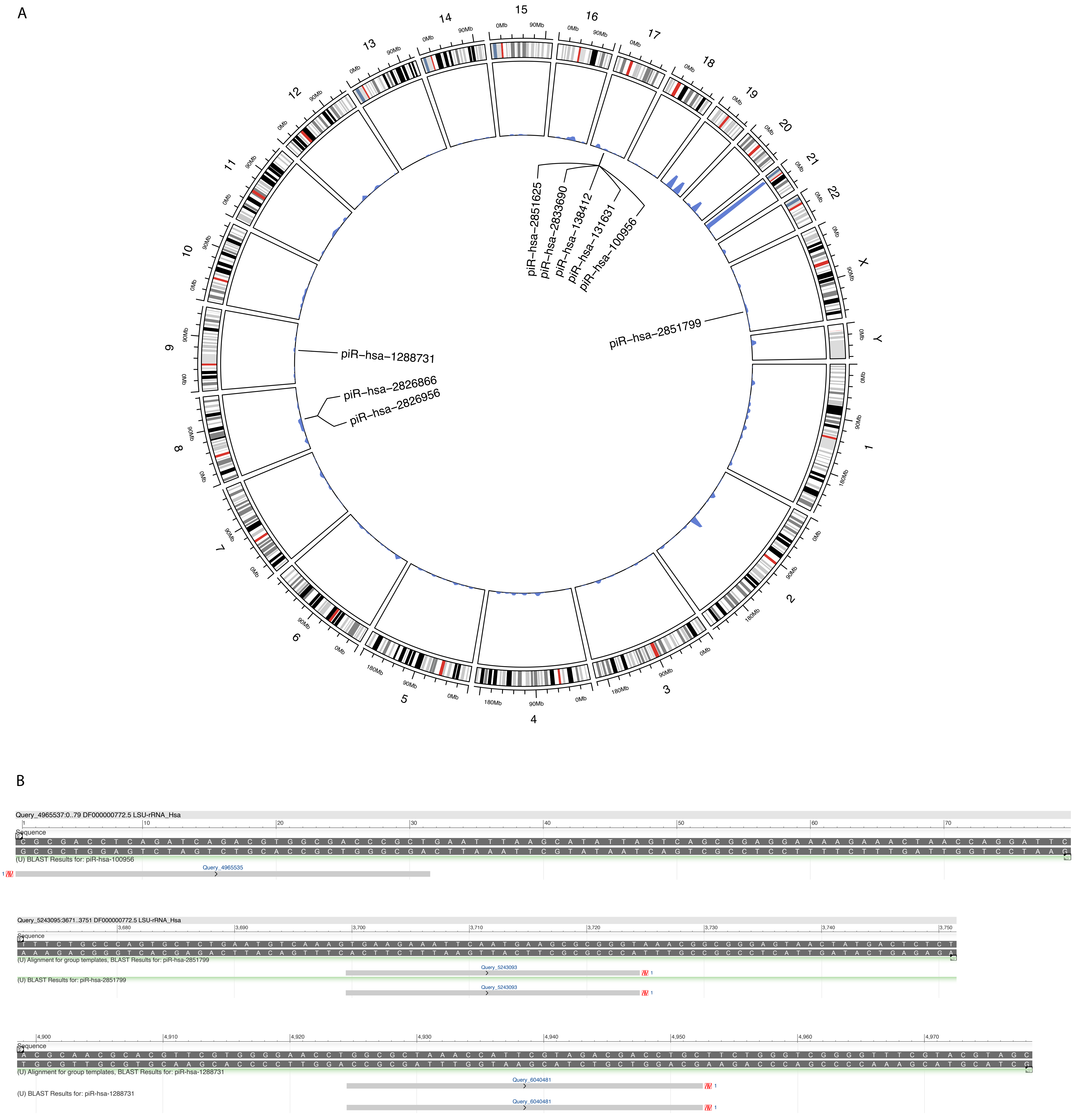

Supplementary Fig 14. piRNA candidates derived from large subnit ribosomal ribonucleic acid (LSU rRNA).
A. Circular genome track showing the distribution of all possible transposon seeds from LSU across all genome and its derived piRNAs out of 13 piRNA candidates.
B. Blast results between LSU and three piRNAs signatures (piR-hsa-100956, piR-hsa-2851799, piR-hsa-1288731) showing the 100% identities match from different sites in LSU.

A

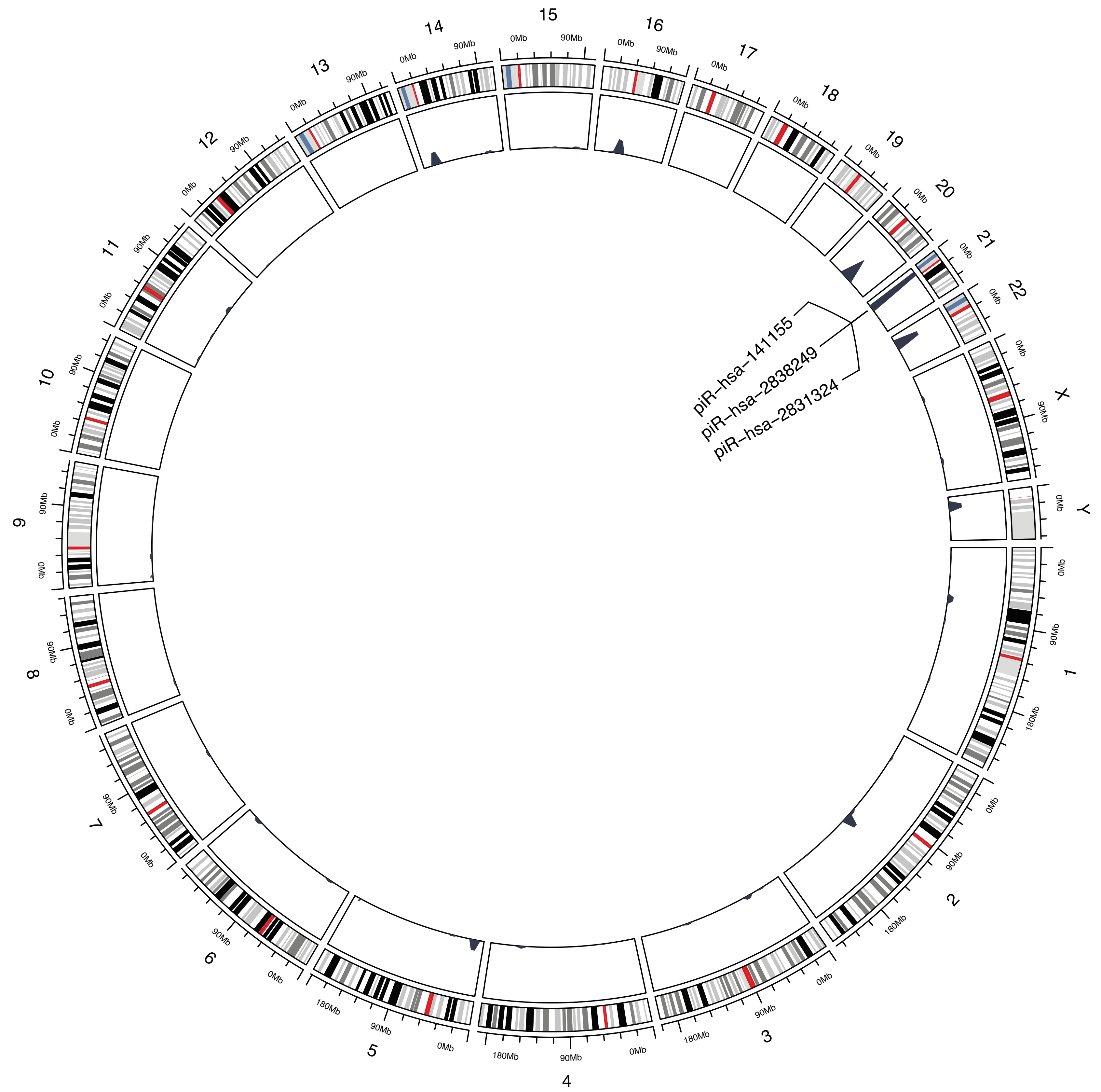

B

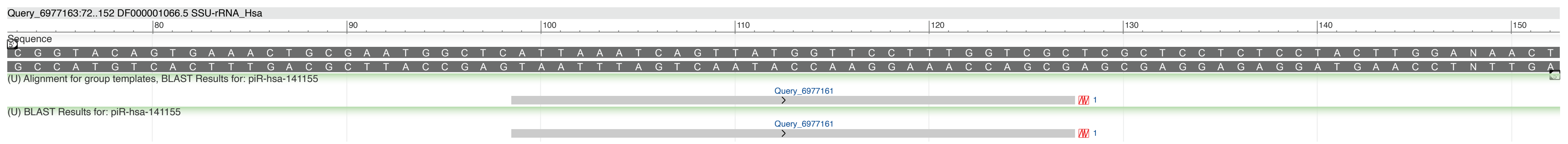

Supplementary Fig 15. piRNA candidates derived from small subnit ribosomal ribonucleic acid (SSU rRNA).
A. Circular genome track showing the distribution of all possible transposon seeds from SSU across all genome and its derived piRNAs out of 13 piRNA candidates.
B. Blast results between SSU and piRNA signature (piR-hsa-14115) showing the 100% identities match.

A

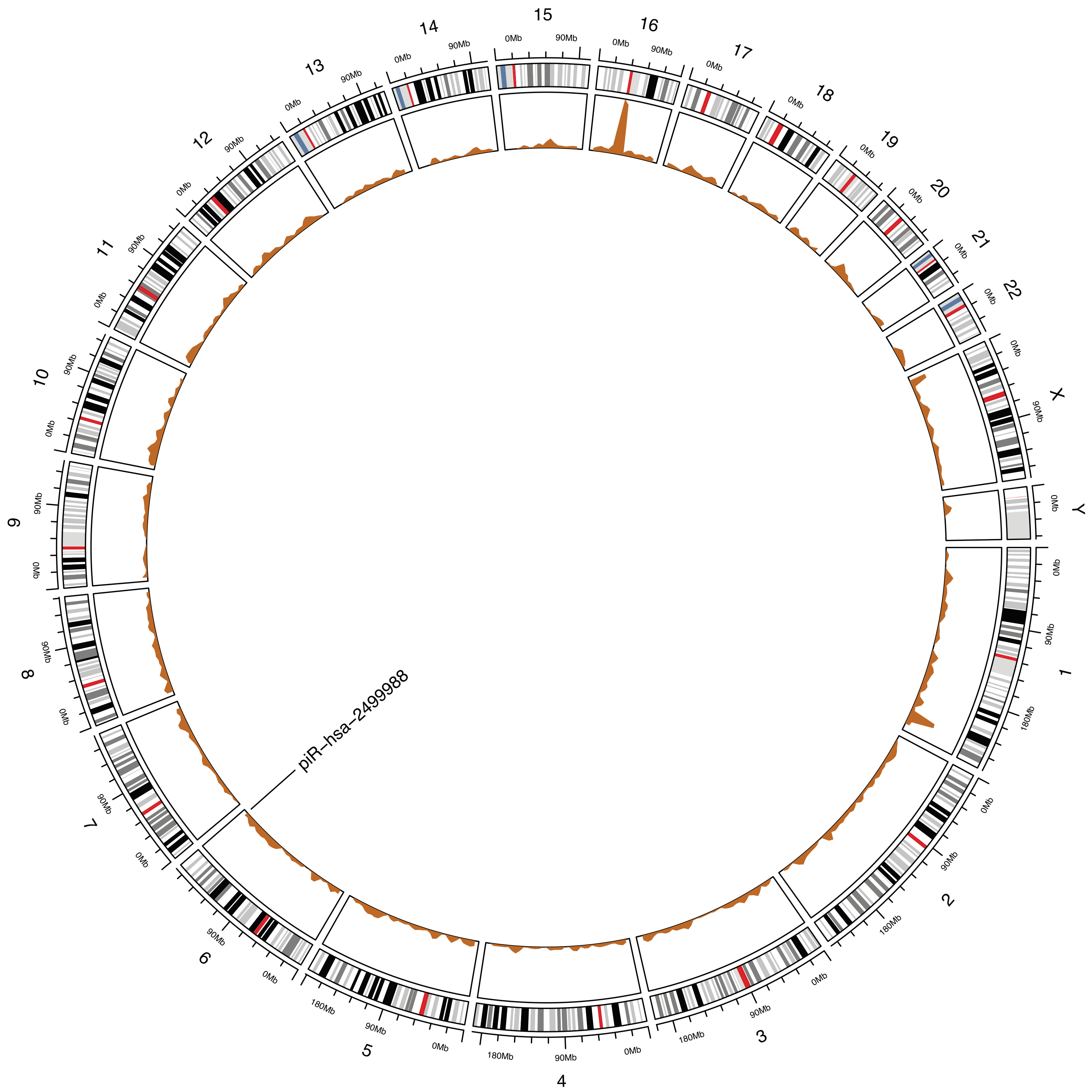

B

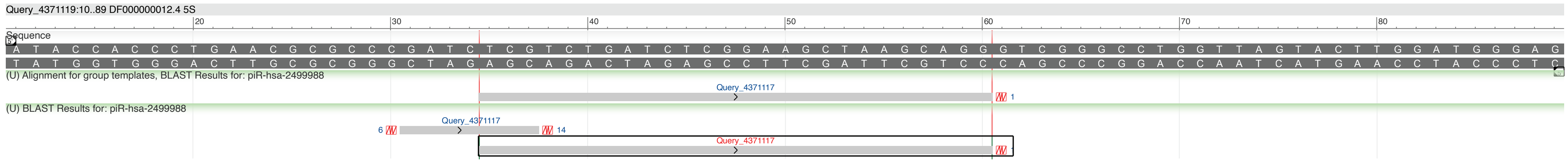

Supplementary Fig 16. piRNA candidates derived from 5S ribosomal ribonucleic acid (5S rRNA).
A. Circular genome track showing the distribution of all possible transposon seeds from 5S across all genome and its derived piRNAs out of 13 piRNA candidates.
B. Blast results between 5S and piRNA signature (piR-hsa-2499988) showing the 100% identities match.

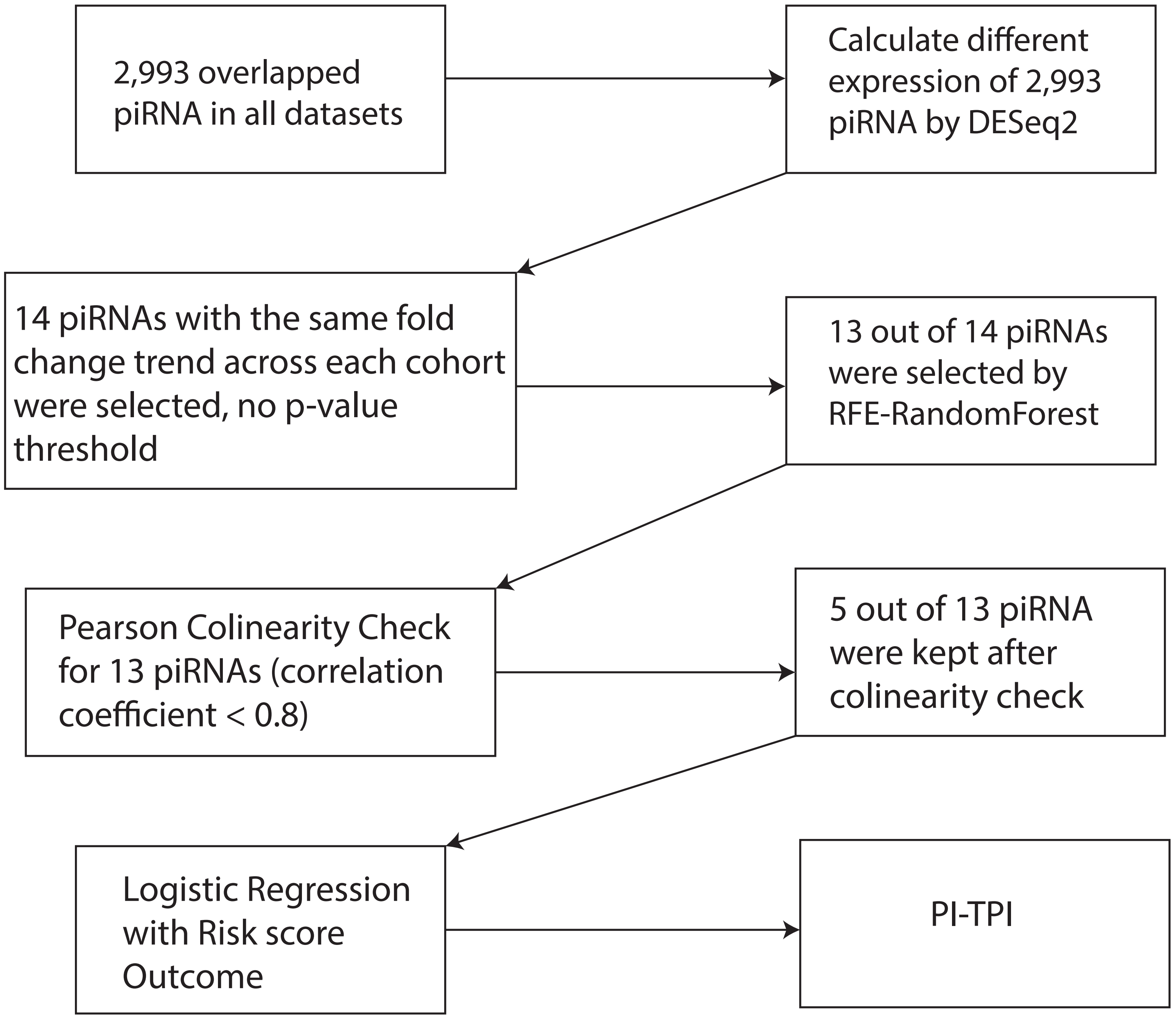

Supplementary Figure 17. Workflow of 5 piRNA signature selection pipeplines
The workflow including eight steps from the downstream analysis presented the whole processing content of how the 5 piRNA signatures were selected.

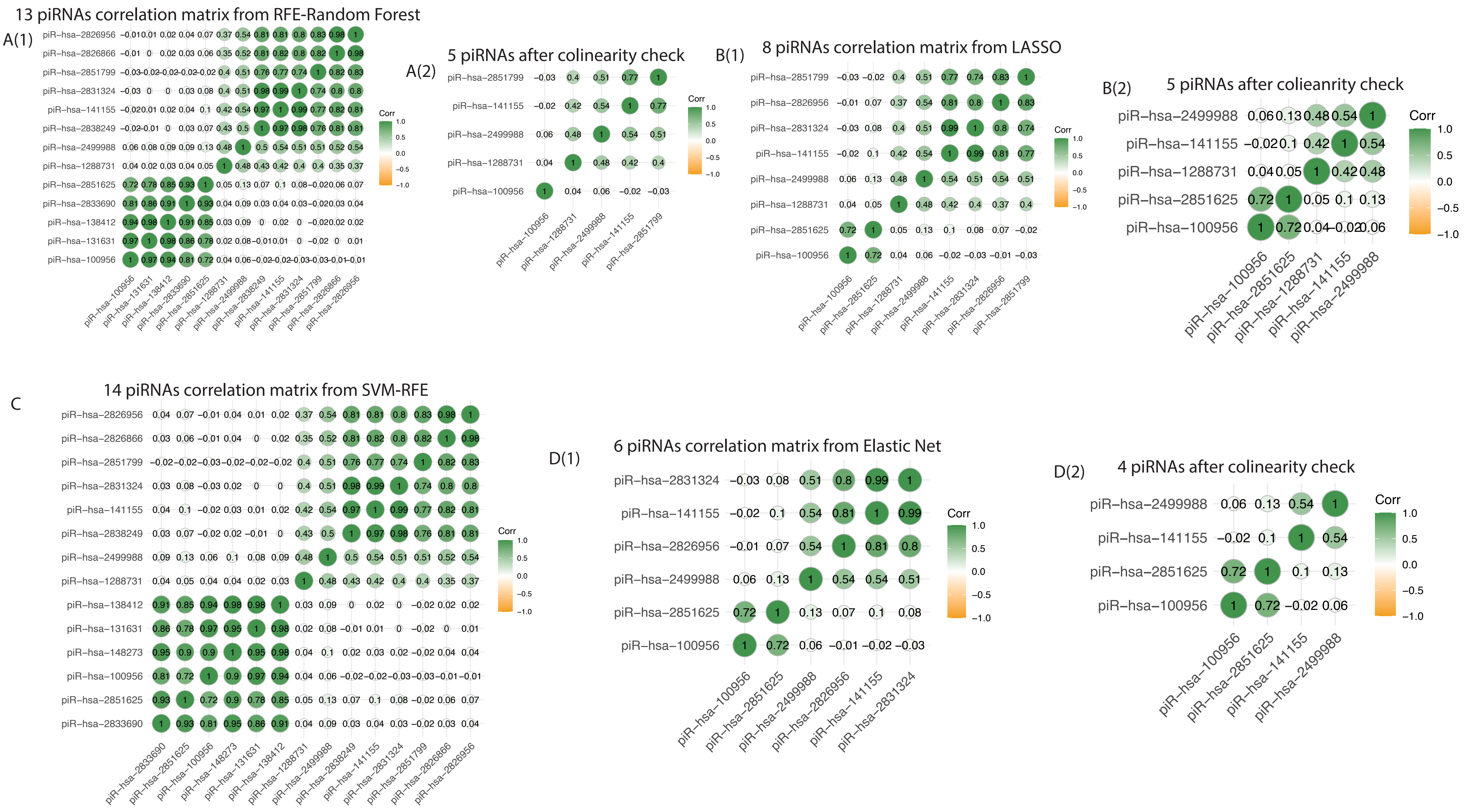

Supplementary Figure 18: Correlation matrix of the piRNA signature before and after collinearity filtering across four different feature selection methods.

A: A(1) correlation matrix of 13 piRNAs out of 14 total feature canididates selected from RFE-Random Forest; A(2) correlation matirx of 5 piRNAs out of 13 piRNA after colinearity check;

B: B(1) correlation matrix of 8 piRNA out of 14 total feature candidiates select from Randomforest-RFE; B(2) correlation matrix of 5 piRNAs out of 8 piRNA after colinearity check;

C: correlation matrix 14 out of out 14 total feature candidate select from SVM-RFE;

D: D(1) correlation matrix of 6 piRNAs out of 14 total feature candidiates selected from SVM-RFE; D(2) correlation matrix of 4 piRNAs out of 6 piRNA after coliearity check.
