# Supplementary material for: A robust machine learning model based on ribosomal‐subunit‐derived piRNAs for diagnostic potential of nonsmall cell lung cancer across multicentre, large‐scale of sequencing data
Source: Clin Transl Med. 2025 Jul 25;15(8):e70418. doi: 10.1002/ctm2.70418 (PMC12410371; doi:10.1002/ctm2.70418)
Supplement: Supplementary file 2 — Supporting Information [file CTM2-15-e70418-s002.pdf]

TableS1-1 Demographic Table

	Tumor	Normal	Benign
**Sample type**			
Tissue	1163	263	-
Plasma	95	73	-
plasma(pooling)	120(12)	60(6)	60(6)
Exosome	71	42	79
**Age**			
>60	879	154	79
<=60	351	87	60
**Sex**			
Female	596	154	70
Male	714	139	69
**Smoking statues**			
Never smoking	307	102	23
Former smoking	73	44	29
current smoking	868	131	27
**Stage**			
Stage I	559		
Stage II	316		
Stage III	189		
Stage IV	33		
**Subtype**			
LUAD	793		
LUSC	645		

Table S1-2 Clinical information in each cohort

	TCGA-LUAD		TCGA-LUSC		GSE110907		GSE175462		GSE83527		GSE62182		GSE148861		GSE148862		GSE204951		RUSH pooling			CHTN pooling			
**Sample type**	tumor	normal	tumor	normal	tumor	normal	tumor	normal	tumor	normal	tumor	normal	tumor	normal	tumor	normal	tumor	normal	tumor	benign	normal	tumor	benign	normal	Total
Tissue	513	46	478	45	48	48	63	77	26	26	35	21													1426
Plasma													36	13	15	12	44	48							168
plasma(pooling)																			120(12)	60(6)	60(6)				240(24)
Exosome																						71	79	42	192
**Age**																									
>60	337	30	367	37	23	23													106	29	40	46	50	24	
<=60	176	16	111	8	25	25													14	31	20	25	29	18	
**Sex**																									
Female	274	26	124	13			56	44	10	10	23	11							78	30	30	31	40	20	
Male	239	20	354	32			21	19	6	6	12	10							42	30	30	40	39	22	
**Smoking statues**																									
Never smoking	163	10	72	10	41	41	23	23	1	1	4	4										3	23	13	
Former smoking							23	23	16	16	10	5										24	29		
current smoking	350	36	406	35	7	7	31	31	9	9	21	12										44	27	1	
**Stage**																									
Stage I	274		230		30				7		18														
Stage II	127		160		6				13		10														
Stage III	85		82		12				5		5														
Stage IV	24		6						1		2														
**Subtype**																									
LUAD	559				48		63		26										60(6)			37			
LUSC			523								35								60(6)			27			

Table S2 piRNA sequence and coordinates

piRNA_ID	piRNA_seq	Chr	Start	End	Strand	Length
piR-hsa-100956	CCGCGACCTCAGATCAGACGTGGCGACCCGCT	chr17	35151226	35151257	-	32
piR-hsa-1288731	GGCGCTAAACCATTCGTAGACGACCTGCC	chr9	76571925	76571953	+	29
piR-hsa-131631	GCGACCTCAGATCAGACGTGGCGACCCGTT	chr17	35151226	35151255	-	30
piR-hsa-138412	CGACCTCAGATCAGACGTGGCGACCCGCCG	chr17	35151225	35151254	-	30
piR-hsa-141155	ATTAAATCAGTTATGGTTCCTTTGGTCGCC	chr21	8988270	8988299	+	30
piR-hsa-2499988	TCGTCTGATCTCGGAAGCTAAGCAGGT	chr6	165409596	165409622	+	27
piR-hsa-2826866	ATTAGTGACGCGCATGAATGGATGAACGAT	chr8	69690214	69690243	-	30
piR-hsa-2826956	AGTGACGCGCATGAATGGATGATCGAGATT	chr8	69690211	69690240	-	30
piR-hsa-2831324	ATCAGTTATGGTTCCTTTGGTCGTT	chr21	8988275	8988299	+	25
piR-hsa-2833690	CTCAGATCAGACGTGGCGGCCCGCT	chr17	35151226	35151250	-	25
piR-hsa-2838249	ATCAGTTATGGTTCCTTTGGCCGC	chr21	8988275	8988298	+	24
piR-hsa-2851625	CAGATCAGACGTGGCGACCCGCTGAATTTAAAC	chr17	35151216	35151248	-	33
piR-hsa-2851799	TGAAGAAATTCAATGAAGCGCGGGTG	chrX	109054183	109054208	+	26

Table S3-1 Feature selection method-RFE-RF/SVM-RFE

RFE-RF/SVM-RFE	AUC	Accuracy	Sensitivity	Specificity	PPV	NPV	Threshold
Training	0.8123	0.7808	0.843	0.675	0.787052811	0.755555556	0.559842
Holdout Validation	0.8025	0.7429	0.794	0.669291339	0.767175573	0.75	0.586697

Table S3-2 Feature selection method-Elastic Net

Elastic Net	AUC	Accuracy	Sensitivity	Specificity	PPV	NPV	Threshold
Training	0.8162	0.7772	0.6712	0.8298	0.6923	0.8156	0.5877304
Holdout Validation	0.8038	0.7571	0.748	0.7623	0.6419	0.8416	0.652106

Table S3-3 Feature selection method-LASSOCV

LASSOCV	AUC	Accuracy	Sensitivity	Specificity	PPV	NPV	Threshold
Training	0.8163	0.7759	0.6847	0.8279	0.6942	0.8215	0.5865535
Holdout Validation	0.8049	0.76	0.7323	0.7758	0.6503	0.8357	0.6450357

Table S4 Model Comparison between pi-TPI and other published NSCLC diagnostic models

	Assay	Model Features	Sample	Model	Metrics	Results	Independent Validation	In-house data	Benign data	Total cohort
**Pi-TPI**	**Small RNA-seq**	**piRNA**	**LUAD vs control vs LUSC**	**RF, LR**	**AUC**	**0.97**	**Yes**	**Yes(n=192)**	**Yes**	**11**
[1]	ELISA, CLIA	IL-6, CEA, CYFRA21-1	LUAD vs benign vs control	NA	AUC	0.767	No	Yes	Yes	1
[2]	Immunoassay	CEA	LUAD vs benign vs control	NA	AUC	0.76	No	Yes (n=212)	Yes	1
[3]	RNA-seq	mRNAs	LUAD vs control	DBN+Rprop +SVM	Acc.	95.97%	No	No	No	1
[4]	RNA-seq	mRNAs	LUAD vs control	SVM	Acc.	91%	Yes	No	No	1
[5]	RNA-seq	mRNAs	LUAD vs. control vs. LUSC	mRMR	F1 score	95.4%	No	No	No	1
[6]	miRNA-seq	miRNA	LUSC vs. control	SVM	F1 score	99.4%	No	No	No	3
[7]	CNV	mRNA	LUAD vs control vs LUSC	EN-PLS-NB	Acc.	84%	Yes	No	No	3
[8]	metDNA	DNA methylation	LUAD vs. control	RF	Acc.	94.57%	Yes	Yes (n=25)	No	7
[9]	metDNA	DNA methylation	LADC vs. SQCLC vs. SCLC	Ensemble	Acc.	86.54%	Yes	No	No	2
[10]	WSI	whole-slide images	LUAD vs. control vs. LUSC	CNN	AUC	0.97	Yes	Yes(n=340)	Yes	4
[11]	WSI	whole-slide images	Lung carcinoma vs. control	CNN	AUC	0.988	Yes	Yes (n=4704)	No	4

[12]	Microarray	mRNA	LUAD vs. control	CNN	AUC	0.816	Yes	No	No	6
[13]	RT-qPCR, Radiographic images	miRNA, images	Lung cancer vs control	RF, KNN, NN, SVM	AUC	0.86	No	Yes(n=205)	Yes	1
[14]	RNA-Seq, miRNA-Seq, whole-slide imaging, copy number variation, DNA methylation	mRNA, miRNA, whole-slide images DNA methylation	LUAD vs. control vs. LUSC	Resnet-18, SVM, ANN	AUC	0.993	No	No	No	1
[15]	RT-qPCR	piRNA	LUAD vs. control vs. LUSC	Decision Tree	AUC	0.74	No	Yes(n=308)	No	1
[16]	RT-qPCR	miRNA	LUAD vs control vs LUSC	MLR	AUC	0.795	No	Yes(n=60)	No	1
[17]	RNA-seq,miRNA-seq, DNA methylation, Clinical information	mRNA, miRNA, DNA methylation, clinical characteristics	LUAD vs control vs LUSC	GAT	AUC	0.82	No	No	No	1
[18]	Microarray	miRNA	Multi-cancer (lung cancer vs non-cancer)	10-fold cross validation	AUC	1.00	Yes	No	No	8
[19]	miRNA-seq	miRNA	LUAD vs control vs LUSC vs SCLC	MR	AUC	0.806	Yes	Yes (n=310)	No	2
[20]	RT-qPCR,	miRNA	NSCLC vs Pulmonary	LASSO	Sensitivity, Specificity	89.9%, 90.9%	Yes	Yes (n=359)	Yes	3

	Radiology images		nodules vs non nodules						
[21]	miRNA-seq, RT-qPCR	miRNA	LUAD vs GGN vs control	NA	Sensitivity, Specificity	0.864, 0.060	No	Yes (n=132)	Yes	1
[22]	PET/CT	Image	CPTAC-LUAD, CPTAC-LSCC, TCGA-LUAD, TCGA-LUSC, In-house images	CNN	Accuracy Sensitivity Specificity AUC	0.8649 0.8000 0.8412 0.8206	Yes	Yes(n=98)	No	6
[23]	CT	Image	TCGA-LUAD TCGA-LUSC NSCLC	Random Forest	AUC	0.85	Yes	No	No	3
[24]	CT	Image	Kaggle	CNN	Accuracy	0.87	No	No	Yes	1
[25]	CT	Image	NIH research dataset (NLST, Northwestern University)	CNN	AUC	0.944	Yes	No	Yes	3
[26]	CT	Image	Kaggle	CNN/Transformer	AUC	1.0	No	No	Yes	1
[27]	CT	Image	NLST, In-house data	Transformer	AUC	0.919	No	Yes	No	2

[28]	CT	Image	LIDC-IDRI	CNN/transformer	AUC	0.9628	No	No	Yes	1

Abbreviation:
LUAD/LADC: lung adenocarcinoma
LUSC: lung squamous cell
SQCLC: squamous cell lung cancer
RF: Random Forest
LR: logistic regression
ELISA: chemiluminescence immunoassay
CLIA: Chemiluminescence Immunoassay
DBN: deep belief network
Rprop: resilient backpropagation
EN: elastic net
PLS: partial least squares
NB: naïve bayes
MR: multivariate regression
K-NN: K-Nearest Neighbors
NN: Neural Networks
SVM: Support Vector Machines
LASSO: least absolute shrinkage and selection operator
GAT: graph attention network
MR: Multivariate Regression
GGN: ground-glass nodules
mRMR: Minimum Redundancy Maximum Relevance
CNN: Convolutional Neural Network
CT: Computed Tomography
LDCT: Low-dose Chest Computed Tomography

Reference:

1. Pan J, Zhuang W, Xia Y, Huang Z, Zheng Y, Wang X, Huang Y: **Combined detection of serum IL-6 and CEA contributes to the diagnosis of lung adenocarcinoma in situ.** *PeerJ* 2024, **12:**e17141.
2. Zhang L, Zhuang J, Pang Y, Song Y, Li X, Liu K, Li S, Sun T: **The Evaluation of Urinary HE4, CEA, ProGRP, CYFRA 21-1 and NSE in the Diagnosis of Lung Cancer.** *Ann Clin Lab Sci* 2024, **54:**845-855.
3. Smolander J, Stupnikov A, Glazko G, Dehmer M, Emmert-Streib F: **Comparing biological information contained in mRNA and non-coding RNAs for classification of lung cancer patients.** *BMC Cancer* 2019, **19:**1176.
4. Fan Z, Xue W, Li L, Zhang C, Lu J, Zhai Y, Suo Z, Zhao J: **Identification of an early diagnostic biomarker of lung adenocarcinoma based on co-expression similarity and construction of a diagnostic model.** *J Transl Med* 2018, **16:**205.
5. Castillo-Secilla D, Galvez JM, Carrillo-Perez F, Verona-Almeida M, Redondo-Sanchez D, Ortuno FM, Herrera LJ, Rojas I: **KnowSeq R-Bioc package: The automatic smart gene expression tool for retrieving relevant biological knowledge.** *Comput Biol Med* 2021, **133:**104387.
6. Ye Z, Sun B, Xiao Z: **Machine learning identifies 10 feature miRNAs for lung squamous cell carcinoma.** *Gene* 2020, **749:**144669.
7. Qiu ZW, Bi JH, Gazdar AF, Song K: **Genome-wide copy number variation pattern analysis and a classification signature for non-small cell lung cancer.** *Genes Chromosomes Cancer* 2017, **56:**559-569.
8. Shen N, Du J, Zhou H, Chen N, Pan Y, Hoheisel JD, Jiang Z, Xiao L, Tao Y, Mo X: **A Diagnostic Panel of DNA Methylation Biomarkers for Lung Adenocarcinoma.** *Front Oncol* 2019, **9:**1281.
9. Cai Z, Xu D, Zhang Q, Zhang J, Ngai SM, Shao J: **Classification of lung cancer using ensemble-based feature selection and machine learning methods.** *Mol Biosyst* 2015, **11:**791-800.
10. Coudray N, Ocampo PS, Sakellaropoulos T, Narula N, Snuderl M, Fenyo D, Moreira AL, Razavian N, Tsirigos A: **Classification and mutation prediction from non-small cell lung cancer histopathology images using deep learning.** *Nat Med* 2018, **24:**1559-1567.
11. Kanavati F, Toyokawa G, Momosaki S, Rambeau M, Kozuma Y, Shoji F, Yamazaki K, Takeo S, Iizuka O, Tsuneki M: **Weakly-supervised learning for lung carcinoma classification using deep learning.** *Sci Rep* 2020, **10:**9297.
12. Lai YH, Chen WN, Hsu TC, Lin C, Tsao Y, Wu S: **Overall survival prediction of non-small cell lung cancer by integrating microarray and clinical data with deep learning.** *Sci Rep* 2020, **10:**4679.
13. Poh KC, Ren TM, Ling GL, Goh JSY, Rose S, Wong A, Mehta SS, Goh A, Chong PY, Cheng SW, et al: **Development of a miRNA-Based Model for Lung Cancer Detection.** *Cancers (Basel)* 2025, **17**.
14. Carrillo-Perez F, Morales JC, Castillo-Secilla D, Gevaert O, Rojas I, Herrera LJ: **Machine-Learning-Based Late Fusion on Multi-Omics and Multi-Scale Data for Non-Small-Cell Lung Cancer Diagnosis.** *J Pers Med* 2022, **12**.
15. He Y, Altuna-Coy A, Acosta-Plasencia M, Molins L, Sanchez-Lorente D, Martinez D, Diaz T, Na R, Marrades RM, Navarro A: **Diagnostic and Prognostic Value of hsa_piR_022710, hsa_piR_019822, and hsa_piR_020840 in Early-Stage Non-Small-Cell Lung Cancer: Implications for Recurrence and Survival in Squamous Cell Carcinoma Patients.** *Int J Mol Sci* 2025, **26**.
16. Alizadeh N, Zahedi H, Koopaie M, Fatahzadeh M, Mousavi R, Kolahdooz S: **Diagnosis of lung cancer using salivary miRNAs expression and clinical characteristics.** *BMC Pulm Med* 2025, **25:**41.

17. Elbashir MK, Almotilag A, Mahmood MA, Mohammed M: **Enhancing Non-Small Cell Lung Cancer Survival Prediction through Multi-Omics Integration Using Graph Attention Network.** *Diagnostics (Basel)* 2024, **14**.
18. Zhang J, Rui H, Hu H: **Noninvasive multi-cancer detection using blood-based cell-free microRNAs.** *Sci Rep* 2024, **14:**22136.
19. Fotopoulos I, Nguyen OTD, Nost TH, Markaki M, Lagani V, Mjelle R, Sandanger TM, Saetrom P, Tsamardinos I, Roe OD: **Promising microRNAs in pre-diagnostic serum associated with lung cancer up to eight years before diagnosis: a HUNT study.** *J Cancer Res Clin Oncol* 2024, **150:**355.
20. Lin Y, Leng Q, Jiang Z, Guarnera MA, Zhou Y, Chen X, Wang H, Zhou W, Cai L, Fang H, et al: **A classifier integrating plasma biomarkers and radiological characteristics for distinguishing malignant from benign pulmonary nodules.** *Int J Cancer* 2017, **141:**1240-1248.
21. He Y, Yang Y, Kuang P, Ren S, Rozeboom L, Rivard CJ, Li X, Zhou C, Hirsch FR: **Seven-microRNA panel for lung adenocarcinoma early diagnosis in patients presenting with ground-glass nodules.** *Onco Targets Ther* 2017, **10:**5915-5926.
22. Choi J, Cho HH, Kwon J, Lee HY, Park H: **A Cascaded Neural Network for Staging in Non-Small Cell Lung Cancer Using Pre-Treatment CT.** *Diagnostics (Basel)* 2021, **11**.
23. Yu L, Tao G, Zhu L, Wang G, Li Z, Ye J, Chen Q: **Prediction of pathologic stage in non-small cell lung cancer using machine learning algorithm based on CT image feature analysis.** *BMC Cancer* 2019, **19:**464.
24. Mohamed TIA, Oyelade ON, Ezugwu AE: **Automatic detection and classification of lung cancer CT scans based on deep learning and ebola optimization search algorithm.** *PLoS One* 2023, **18:**e0285796.
25. Ardila D, Kiraly AP, Bharadwaj S, Choi B, Reicher JJ, Peng L, Tse D, Etemadi M, Ye W, Corrado G, et al: **End-to-end lung cancer screening with three-dimensional deep learning on low-dose chest computed tomography.** *Nat Med* 2019, **25:**954-961.
26. Durgam R, Panduri B, Balaji V, Khadidos AO, Khadidos AO, Selvarajan S: **Enhancing lung cancer detection through integrated deep learning and transformer models.** *Sci Rep* 2025, **15:**15614.
27. Song P, Hou J, Xiao N, Zhao J, Zhao J, Qiang Y, Yang Q: **MSTS-Net: malignancy evolution prediction of pulmonary nodules from longitudinal CT images via multi-task spatial-temporal self-attention network.** *Int J Comput Assist Radiol Surg* 2023, **18:**685-693.
28. Liu D, Liu F, Tie Y, Qi L, Wang F: **Res-trans networks for lung nodule classification.** *Int J Comput Assist Radiol Surg* 2022, **17:**1059-1068.

Table S5 13 piRNA candidates differentially expression analysis by DEseq2

piRNA	baseMean	log2FoldChange	lfcSE	stat	pvalue	padj	batch
piR-hsa-100956	67075.729	-0.043010329	0.237994282	-0.18072	0.856587351	0.959335549	TCGA
piR-hsa-100956	247791.1975	-3.428653496	0.222663986	-15.39833	1.67951549477768e-53	3.22418493382799e-52	GSE175462
piR-hsa-100956	520221.2024	-2.700463593	0.251047714	-10.756774	5.50639962758951e-27	1.58763529153038e-25	GSE110907
piR-hsa-100956	243284.7941	-3.123909832	0.326758227	-9.5603096	1.17404909698914e-21	1.98808860104596e-20	GSE83527
piR-hsa-100956	16361.53785	-1.669121392	0.239049531	-6.9823245	2.90335583953001e-12	1.5251181611921e-11	GSE62182
piR-hsa-100956	34855.44477	-0.880759996	0.24663829	-3.5710594	0.00035554	0.002188936	GSE148861/GSE148862
piR-hsa-100956	291.5216145	-0.304856808	0.069822702	-4.366156	1.26452E-05	6.25622E-05	GSE204951
piR-hsa-100956	41.42091586	-0.067214305	0.107196749	-0.6270181	0.530647353	0.561037869	CHTN
piR-hsa-1288731	652.7600767	-0.379320831	0.122985315	-3.0842774	0.002040472	0.024548905	TCGA
piR-hsa-1288731	863.2019641	-1.521165789	0.167948153	-9.0573535	1.33653922118087e-19	5.60002799123783e-19	GSE175462
piR-hsa-1288731	14998.03839	-0.738195279	0.165049779	-4.4725615	7.72881779216233e-06	3.50935E-05	GSE110907
piR-hsa-1288731	1208.831231	-1.340534997	0.307893216	-4.3538958	1.33739E-05	4.1081E-05	GSE83527
piR-hsa-1288731	195.7204361	-1.196504342	0.233576616	-5.1225348	3.01455440393564e-07	1.02144259754933e-06	GSE62182
piR-hsa-1288731	1860.51893	-0.303063648	0.171716559	-1.7649064	0.077579505	0.166349815	GSE148861/GSE148862
piR-hsa-1288731	381.1751094	-0.483487666	0.197826349	-2.4440004	0.014525412	0.02579725	GSE204951
piR-hsa-1288731	3.080154426	-0.949034244	0.270303497	-3.5109951	0.000446433	0.000835183	CHTN
piR-hsa-131631	66854.60654	-0.043540296	0.239441475	-0.1818411	0.855707447	0.959335549	TCGA
piR-hsa-131631	104128.4801	-3.008827722	0.215240114	-13.978936	2.09598940814071e-44	2.97321148121647e-43	GSE175462
piR-hsa-131631	8442.288345	-2.611519431	0.272697908	-9.5766024	1.00289904699841e-21	2.64096749042916e-20	GSE110907
piR-hsa-131631	100724.2363	-2.763540285	0.312274363	-8.8497188	8.77401214246311e-19	1.152715487149e-17	GSE83527
piR-hsa-131631	9013.087716	-1.489887841	0.23133753	-6.4403205	1.19221453467025e-10	5.67400719207696e-10	GSE62182
piR-hsa-131631	33781.09244	-0.836941146	0.249027218	-3.3608421	0.000777052	0.004094648	GSE148861/GSE148862
piR-hsa-131631	281.1201471	-0.309776214	0.070910415	-4.3685573	1.2507E-05	6.25622E-05	GSE204951
piR-hsa-131631	41.38620148	-0.067965376	0.107237645	-0.6337828	0.526222573	0.561037869	CHTN
piR-hsa-138412	66854.55367	-0.04353852	0.239441484	-0.1818337	0.855713274	0.959335549	TCGA
piR-hsa-138412	55103.53848	-2.828390981	0.20805301	-13.594569	4.31294289091106e-42	5.53455164706966e-41	GSE175462
piR-hsa-138412	2903.172897	-2.472310663	0.251121151	-9.8450913	7.19738357222326e-23	1.99151968792837e-21	GSE110907
piR-hsa-138412	56177.38009	-2.504034852	0.303706748	-8.2449102	1.6528435317171e-16	1.9313635595377e-15	GSE83527
piR-hsa-138412	3522.291623	-0.978831215	0.213460075	-4.585547	4.52799297565158e-06	1.34667E-05	GSE62182
piR-hsa-138412	30356.48163	-0.631378095	0.250906233	-2.5163906	0.011856369	0.039651398	GSE148861/GSE148862
piR-hsa-138412	280.0382266	-0.309251172	0.070659982	-4.3766099	1.20539E-05	6.25622E-05	GSE204951
piR-hsa-138412	41.38620148	-0.067965376	0.107237645	-0.6337828	0.526222573	0.561037869	CHTN
piR-hsa-141155	78.54216948	0.323906681	0.16290762	1.98828441	0.046780243	0.180159628	TCGA
piR-hsa-141155	175.4680501	0.32875479	0.156146563	2.10542444	0.035254378	0.04619015	GSE175462
piR-hsa-141155	675.9914884	0.322510585	0.145826797	2.21160028	0.026994296	0.050308454	GSE110907

piR-hsa-141155	300.2459206	0.387751441	0.249325969	1.55519877	0.119898737	0.160757898	GSE83527
piR-hsa-141155	79.83149635	0.40045481	0.292494722	1.36910098	0.170967713	0.235461784	GSE62182
piR-hsa-141155	61.98810168	0.086833796	0.196168601	0.4426488	0.658019782	0.769208369	GSE148861/GSE148862
piR-hsa-141155	26.54260952	0.205285368	0.166421561	1.23352628	0.21737948	0.286757074	GSE204951
piR-hsa-141155	67.78858576	0.628115179	0.117719493	5.33569389	9.51798615074229e-08	8.06137158637534e-07	CHTN
piR-hsa-2499988	1090.304967	-0.873388546	0.211117951	-4.1369696	3.51923E-05	0.001970429	TCGA
piR-hsa-2499988	350.962468	-0.612205862	0.232593709	-2.6320826	0.008486322	0.011703172	GSE175462
piR-hsa-2499988	66.41270408	-0.042351055	0.211343114	-0.20039	0.84117555	0.882882049	GSE110907
piR-hsa-2499988	685.7107537	-0.152451205	0.328852855	-0.4635849	0.642945203	0.678878714	GSE83527
piR-hsa-2499988	105.5370711	-0.428520009	0.386347028	-1.1091583	0.267361902	0.341413298	GSE62182
piR-hsa-2499988	449.6312907	-0.954762709	0.318700945	-2.995795	0.002737304	0.012000135	GSE148861/GSE148862
piR-hsa-2499988	23.06956284	-0.693300047	0.179686867	-3.8583791	0.000114142	0.000349144	GSE204951
piR-hsa-2499988	10.68439517	-0.607509085	0.215294727	-2.8217555	0.004776157	0.00668362	CHTN
piR-hsa-2826866	30.66871778	0.39652153	0.215203991	1.842538	0.065396493	0.236450656	TCGA
piR-hsa-2826866	66.40902962	0.145306043	0.258754548	0.56155938	0.574416263	0.613288005	GSE175462
piR-hsa-2826866	271.0683914	0.387102445	0.185372515	2.08824078	0.03677612	0.064739749	GSE110907
piR-hsa-2826866	128.7703916	1.301070239	0.33313162	3.90557414	9.40019E-05	0.000255276	GSE83527
piR-hsa-2826866	13.67469435	0.203842657	0.423431657	0.48140627	0.630227778	0.700137946	GSE62182
piR-hsa-2826866	134.2594492	0.683470585	0.168374091	4.05923846	4.9233E-05	0.000439699	GSE148861/GSE148862
piR-hsa-2826866	5.733249588	0.356231995	0.233025613	1.52872464	0.126332722	0.183970491	GSE204951
piR-hsa-2826866	83.32495954	0.718299499	0.129714461	5.53754374	3.06743104436319e-08	3.47473180562574e-07	CHTN
piR-hsa-2826956	40.13347095	0.266797736	0.177719478	1.50122957	0.133296194	0.347805919	TCGA
piR-hsa-2826956	74.96203909	0.346473374	0.250267863	1.38441017	0.166232898	0.197421216	GSE175462
piR-hsa-2826956	399.8934109	0.017380413	0.190370678	0.09129774	0.927256012	0.951698836	GSE110907
piR-hsa-2826956	145.3230753	1.475711668	0.324847175	4.54278744	5.5515221690293e-06	1.76264E-05	GSE83527
piR-hsa-2826956	16.68563373	0.413124961	0.422582631	0.97761936	0.328262619	0.399308857	GSE62182
piR-hsa-2826956	127.5472266	0.63680361	0.175714915	3.6240726	0.00029	0.001963078	GSE148861/GSE148862
piR-hsa-2826956	4.443040704	0.719680131	0.239634871	3.00323624	0.00267125	0.005679521	GSE204951
piR-hsa-2826956	72.41880111	0.687894842	0.131135131	5.24569456	1.55694657243401e-07	1.22412319423492e-06	CHTN
piR-hsa-2831324	77.63345276	0.320692199	0.16325837	1.96432317	0.049492626	0.189415422	TCGA
piR-hsa-2831324	161.0357332	0.378602457	0.157780965	2.39954456	0.016415482	0.022078741	GSE175462
piR-hsa-2831324	522.2711131	0.353835711	0.145201146	2.43686583	0.014815175	0.030127782	GSE110907
piR-hsa-2831324	280.0212438	0.377703009	0.253648328	1.4890814	0.136465934	0.180310113	GSE83527
piR-hsa-2831324	71.64756901	0.459249828	0.291956089	1.57300993	0.115716519	0.165836926	GSE62182
piR-hsa-2831324	56.49259206	0.164803598	0.195366485	0.84356126	0.398914621	0.557582256	GSE148861/GSE148862
piR-hsa-2831324	16.2547818	0.18849386	0.236346498	0.79753185	0.425142213	0.498026451	GSE204951
piR-hsa-2831324	56.07313128	0.685408862	0.124021353	5.52653914	3.26609428178767e-08	3.61387888711964e-07	CHTN

piR-hsa-2833690	66854.55038	-0.043538663	0.239441498	-0.1818342	0.855712811	0.959335549	TCGA
piR-hsa-2833690	7474.759438	-2.249878647	0.19237941	-11.695008	1.35175217742748e-31	1.00174301803221e-30	GSE175462
piR-hsa-2833690	421.4451383	-1.984060762	0.228892771	-8.6680796	4.39469461989369e-18	1.08888547150184e-16	GSE110907
piR-hsa-2833690	4802.047676	-2.669625902	0.302429753	-8.8272595	1.07271742174739e-18	1.38498788502365e-17	GSE83527
piR-hsa-2833690	565.6910573	-2.636718708	0.284972946	-9.2525229	2.19255242688071e-20	2.10638001754517e-18	GSE62182
piR-hsa-2833690	29964.41001	-0.628313218	0.251970706	-2.4935963	0.012645628	0.041919209	GSE148861/GSE148862
piR-hsa-2833690	2.736204935	-0.374231207	0.289557184	-1.2924259	0.196209688	0.268074811	GSE204951
piR-hsa-2833690	41.38620148	-0.067965376	0.107237645	-0.6337828	0.526222573	0.561037869	CHTN
piR-hsa-2838249	77.42788873	0.320353867	0.163356552	1.96107143	0.049870692	0.19079963	TCGA
piR-hsa-2838249	79.31701209	0.415959495	0.141613519	2.93728663	0.003310979	0.004691758	GSE175462
piR-hsa-2838249	217.9271521	0.086840327	0.141266726	0.61472598	0.538735697	0.632761966	GSE110907
piR-hsa-2838249	134.5947641	0.943283328	0.214262052	4.40247501	1.07023E-05	3.31257E-05	GSE83527
piR-hsa-2838249	38.41879426	0.370970678	0.292258623	1.2693233	0.204325781	0.273826375	GSE62182
piR-hsa-2838249	46.9023187	0.077864419	0.172921842	0.45028678	0.652503673	0.767030778	GSE148861/GSE148862
piR-hsa-2838249	8.438088015	0.330513202	0.262970184	1.25684668	0.208809159	0.278148505	GSE204951
piR-hsa-2838249	55.87090682	0.686426374	0.124468927	5.51484126	3.49094911314632e-08	3.82284494094745e-07	CHTN
piR-hsa-2851625	66854.55176	-0.043538603	0.239441493	-0.181834	0.855713005	0.959335549	TCGA
piR-hsa-2851625	3506.829444	-1.3395621	0.183562396	-7.2975845	2.92979679549964e-13	9.43767305636495e-13	GSE175462
piR-hsa-2851625	44.65788093	-0.051505573	0.255507683	-0.2015813	0.84024406	0.882244124	GSE110907
piR-hsa-2851625	983.8369694	-1.18772377	0.259945071	-4.5691337	4.89744415292054e-06	1.55904E-05	GSE83527
piR-hsa-2851625	55.27789905	0.069939444	0.286604194	0.24402799	0.807209145	0.859317351	GSE62182
piR-hsa-2851625	32030.95994	-0.798735252	0.253111926	-3.1556603	0.001601353	0.007696197	GSE148861/GSE148862
piR-hsa-2851625	3.855694764	-0.017993374	0.215453095	-0.0835141	0.933442755	0.956023558	GSE204951
piR-hsa-2851625	41.38620148	-0.067965376	0.107237645	-0.6337828	0.526222573	0.561037869	CHTN
piR-hsa-2851799	154.0397607	0.342706071	0.184156748	1.86094767	0.062751567	0.232814511	TCGA
piR-hsa-2851799	8.586056451	2.078548917	0.269456376	7.71386057	1.22067824413263e-14	4.06849553484969e-14	GSE175462
piR-hsa-2851799	7.139114299	0.805439955	0.230176766	3.49922353	0.000466615	0.001383099	GSE110907
piR-hsa-2851799	16.77736913	2.479108397	0.387011131	6.4057806	1.49601962752996e-10	9.64476314247102e-10	GSE83527
piR-hsa-2851799	2.904882735	0.829393204	0.524540852	1.58117943	0.113837018	0.163341689	GSE62182
piR-hsa-2851799	11.83810968	0.327281308	0.317069194	1.03220784	0.301974763	0.450517068	GSE148861/GSE148862
piR-hsa-2851799	4.757603408	0.26375681	0.193454543	1.36340458	0.172755039	0.240544409	GSE204951
piR-hsa-2851799	79.04157408	0.585286449	0.116691336	5.01568043	5.2846103157129e-07	3.27510438857746e-06	CHTN

Table S6 genes that five piRNA sigatures signicantly correlated with

ENSEMBL	SYMBOL	piRNA	pearson_corr	p_value_p	FDR_p
ENSG00000000005	TNMD	piR-hsa-1288731	0.536705824	5.90649924337985e-08	0.000135865
ENSG00000005007	UPF1	piR-hsa-141155	0.432425304	2.31748848441722e-05	0.009555267
ENSG00000005007	UPF1	piR-hsa-2499988	0.413146758	5.72594092771079e-05	0.018200164
ENSG00000005102	MEOX1	piR-hsa-2499988	0.421335533	3.92555917710093e-05	0.014062833
ENSG00000005102	MEOX1	piR-hsa-141155	0.443476361	1.34537192995576e-05	0.006474673
ENSG00000005102	MEOX1	piR-hsa-2851799	0.792544483	2.19404100850341e-20	1.91781124553283e-16
ENSG00000007080	CCDC124	piR-hsa-141155	0.387428594	0.000176178	0.038790257
ENSG00000011451	WIZ	piR-hsa-141155	0.392591646	0.000141624	0.033740713
ENSG00000014164	ZC3H3	piR-hsa-141155	0.384893076	0.00019586	0.04165489
ENSG00000019549	SNAI2	piR-hsa-141155	0.42071293	4.04125156608015e-05	0.014129832
ENSG00000020256	ZFP64	piR-hsa-141155	0.382482359	0.000216438	0.043793595
ENSG00000025708	TYMP	piR-hsa-2851799	0.411933092	6.05043458526527e-05	0.019024046
ENSG00000033050	ABCF2	piR-hsa-141155	0.443435627	1.3481185413439e-05	0.006474673
ENSG00000035403	VCL	piR-hsa-2851799	0.384230203	0.000201331	0.042303645
ENSG00000037965	HOXC8	piR-hsa-2851799	0.427828494	2.88977126854485e-05	0.011284801
ENSG00000037965	HOXC8	piR-hsa-141155	0.390878308	0.000152326	0.035167867
ENSG00000042062	RIPOR3	piR-hsa-2851799	0.45799772	6.39372176469232e-06	0.003582533
ENSG00000049247	UTS2	piR-hsa-2851799	0.420816325	4.02182106256858e-05	0.014129832
ENSG00000051128	HOMER3	piR-hsa-141155	0.540063431	4.70574328546873e-08	0.000114258
ENSG00000051128	HOMER3	piR-hsa-2499988	0.459264609	5.9821906330032e-06	0.003462936
ENSG00000060558	GNA15	piR-hsa-2499988	0.390440112	0.00015518	0.035508704
ENSG00000064490	RFXANK	piR-hsa-141155	0.38497155	0.000195222	0.04165489
ENSG00000064545	TMEM161A	piR-hsa-141155	0.425128341	3.28487907274317e-05	0.012323231
ENSG00000067225	PKM	piR-hsa-141155	0.510742657	3.15866198472695e-07	0.000378217
ENSG00000067225	PKM	piR-hsa-2499988	0.437225274	1.83418951328417e-05	0.007976443
ENSG00000067798	NAV3	piR-hsa-1288731	0.459020312	6.0595560921155e-06	0.003475529
ENSG00000071539	TRIP13	piR-hsa-141155	0.378610004	0.000253705	0.048846552
ENSG00000074181	NOTCH3	piR-hsa-141155	0.386475964	0.000183348	0.039866823
ENSG00000074181	NOTCH3	piR-hsa-2499988	0.396499925	0.000119762	0.029995393
ENSG00000074800	ENO1	piR-hsa-141155	0.381916247	0.000221551	0.04451898

ENSG00000075618	FSCN1	piR-hsa-141155	0.387030019	0.000179146	0.039147864
ENSG00000075618	FSCN1	piR-hsa-2499988	0.434139161	2.13268265651543e-05	0.009138127
ENSG00000075624	ACTB	piR-hsa-2851799	0.383447963	0.000207967	0.043281954
ENSG00000078098	FAP	piR-hsa-1288731	0.487360673	1.27505898076738e-06	0.001067117
ENSG00000079841	RIMS1	piR-hsa-1288731	0.468979357	3.55982341226723e-06	0.002369454
ENSG00000080200	CRYBG3	piR-hsa-1288731	0.389004122	0.000164886	0.036767048
ENSG00000087303	NID2	piR-hsa-100956	-0.410540763	6.44373254270653e-05	0.019832629
ENSG00000090861	AARS1	piR-hsa-141155	0.384830398	0.000196372	0.041662264
ENSG00000091127	PUS7	piR-hsa-141155	0.399052058	0.000107221	0.028229382
ENSG00000091129	NRCAM	piR-hsa-141155	0.389438381	0.000161893	0.036471893
ENSG00000091140	DLD	piR-hsa-141155	0.4072102	7.48301219017163e-05	0.021875923
ENSG00000092345	DAZL	piR-hsa-2851799	0.406006503	7.89547213943442e-05	0.0225537
ENSG00000099942	CRKL	piR-hsa-2499988	0.48141247	1.78922248801936e-06	0.00144558
ENSG00000099942	CRKL	piR-hsa-2851799	0.790813765	3.02597349744443e-20	2.20416952843015e-16
ENSG00000099942	CRKL	piR-hsa-141155	0.533464446	7.33840091756357e-08	0.000164474
ENSG00000099949	LZTR1	piR-hsa-2499988	0.462170465	5.13039357613677e-06	0.003203198
ENSG00000099949	LZTR1	piR-hsa-141155	0.513202518	2.71072485283431e-07	0.000347424
ENSG00000099949	LZTR1	piR-hsa-2851799	0.844879316	2.32242271390504e-25	4.06005938844879e-21
ENSG00000099957	P2RX6	piR-hsa-141155	0.415376403	5.17162338813653e-05	0.017188274
ENSG00000099957	P2RX6	piR-hsa-2499988	0.446661503	1.14612891783144e-05	0.005818553
ENSG00000099957	P2RX6	piR-hsa-2851799	0.788296902	4.80369215989682e-20	3.22992870535832e-16
ENSG00000099960	SLC7A4	piR-hsa-2851799	0.421077761	3.97307872727315e-05	0.01411735
ENSG00000100023	PPIL2	piR-hsa-2499988	0.468894787	3.57619185991921e-06	0.002369454
ENSG00000100023	PPIL2	piR-hsa-141155	0.476865514	2.30827564433351e-06	0.00175449
ENSG00000100023	PPIL2	piR-hsa-2851799	0.799102182	6.30899873321198e-21	7.87813684671513e-17
ENSG00000100029	PES1	piR-hsa-141155	0.381715663	0.000223389	0.04468291
ENSG00000100030	MAPK1	piR-hsa-141155	0.540339705	4.61805085918485e-08	0.000114258
ENSG00000100030	MAPK1	piR-hsa-2499988	0.511288946	3.0535010366212e-07	0.000375431
ENSG00000100030	MAPK1	piR-hsa-2851799	0.855120149	1.49679003942074e-26	3.27086043364417e-22
ENSG00000100280	AP1B1	piR-hsa-141155	0.454877924	7.52344498639829e-06	0.004084623
ENSG00000100280	AP1B1	piR-hsa-2499988	0.406192615	7.83034829361826e-05	0.022460712
ENSG00000100292	HMOX1	piR-hsa-141155	0.515461174	2.35309099094705e-07	0.000331748

ENSG00000100292	HMOX1	piR-hsa-2499988	0.398309006	0.00011074	0.028808945
ENSG00000100304	TTLL12	piR-hsa-141155	0.453489763	8.08418370405739e-06	0.004335206
ENSG00000100345	MYH9	piR-hsa-141155	0.422969988	3.63637174425302e-05	0.013134515
ENSG00000100401	RANGAP1	piR-hsa-141155	0.461723748	5.25346282315858e-06	0.003256774
ENSG00000100401	RANGAP1	piR-hsa-2499988	0.389937507	0.000158516	0.035803247
ENSG00000100714	MTHFD1	piR-hsa-141155	0.421138327	3.96186564149484e-05	0.01411735
ENSG00000100979	PLTP	piR-hsa-2499988	0.42581849	3.17935444619997e-05	0.01214257
ENSG00000100979	PLTP	piR-hsa-2851799	0.409950114	6.61765138908098e-05	0.020041338
ENSG00000100979	PLTP	piR-hsa-141155	0.528708224	1.00497059354162e-07	0.000204289
ENSG00000100985	MMP9	piR-hsa-2851799	0.794158198	1.62130557443643e-20	1.57464800290543e-16
ENSG00000100985	MMP9	piR-hsa-141155	0.4868278	1.31469341492609e-06	0.001084126
ENSG00000100985	MMP9	piR-hsa-2499988	0.408739422	6.98787005021122e-05	0.020775841
ENSG00000101000	PROCR	piR-hsa-141155	0.487736447	1.24779117551763e-06	0.001058926
ENSG00000101000	PROCR	piR-hsa-2499988	0.385636258	0.000189891	0.040983582
ENSG00000101150	TPD52L2	piR-hsa-141155	0.38083657	0.000231612	0.045700285
ENSG00000101189	MRGBP	piR-hsa-141155	0.39448111	0.000130631	0.03203216
ENSG00000101220	ADISSP	piR-hsa-141155	0.517335213	2.09082303103941e-07	0.000306045
ENSG00000101361	NOP56	piR-hsa-141155	0.45888182	6.10383114471784e-06	0.003475529
ENSG00000101365	IDH3B	piR-hsa-141155	0.414707504	5.33243662628579e-05	0.0173921
ENSG00000101384	JAG1	piR-hsa-141155	0.462329802	5.08715681621736e-06	0.003199053
ENSG00000101444	AHCY	piR-hsa-141155	0.45938083	5.94571194605832e-06	0.003462936
ENSG00000101444	AHCY	piR-hsa-2499988	0.382828935	0.000213362	0.043371955
ENSG00000102359	SRPX2	piR-hsa-1288731	0.397646544	0.000113968	0.029213944
ENSG00000102445	RUBCNL	piR-hsa-2851799	0.429951305	2.61080334562785e-05	0.010664034
ENSG00000103034	NDRG4	piR-hsa-141155	0.429733504	2.63822187592054e-05	0.010676249
ENSG00000103035	PSMD7	piR-hsa-141155	0.406465201	7.73586566922679e-05	0.022421466
ENSG00000103047	TANGO6	piR-hsa-141155	0.39079167	0.000152886	0.035167867
ENSG00000103061	SLC7A6OS	piR-hsa-141155	0.400772772	9.94644096695108e-05	0.026587719
ENSG00000103257	SLC7A5	piR-hsa-2499988	0.383261738	0.000209577	0.043303128
ENSG00000103257	SLC7A5	piR-hsa-141155	0.475185349	2.53375503260998e-06	0.001892953
ENSG00000104142	VPS18	piR-hsa-2499988	0.42542747	3.23874671920843e-05	0.012255362
ENSG00000104517	UBR5	piR-hsa-141155	0.44050514	1.56009292129794e-05	0.007292392

ENSG00000104524	PYCR3	piR-hsa-141155	0.384939922	0.000195479	0.04165489
ENSG00000104974	LILRA1	piR-hsa-2851799	0.397292599	0.000115728	0.029578388
ENSG00000105143	SLC1A6	piR-hsa-2499988	0.383310804	0.000209152	0.043303128
ENSG00000105143	SLC1A6	piR-hsa-141155	0.46149185	5.31844000958079e-06	0.003273837
ENSG00000105220	GPI	piR-hsa-141155	0.45968948	5.84984735399814e-06	0.00343178
ENSG00000105220	GPI	piR-hsa-2499988	0.390814072	0.000152741	0.035167867
ENSG00000105246	EBI3	piR-hsa-2851799	0.730747655	4.34620319493319e-16	2.23471541923006e-12
ENSG00000105329	TGFB1	piR-hsa-141155	0.409864281	6.64328553302188e-05	0.020041338
ENSG00000105364	MRPL4	piR-hsa-141155	0.385655327	0.00018974	0.040983582
ENSG00000105643	ARRDC2	piR-hsa-2851799	0.379817729	0.000241491	0.047223202
ENSG00000105656	ELL	piR-hsa-141155	0.488148673	1.21851251978755e-06	0.001044217
ENSG00000105656	ELL	piR-hsa-2499988	0.491896149	9.80542154373265e-07	0.000865749
ENSG00000105669	COPE	piR-hsa-141155	0.432811098	2.27462755922052e-05	0.009467866
ENSG00000105669	COPE	piR-hsa-2499988	0.398143647	0.000111538	0.02893036
ENSG00000105671	DDX49	piR-hsa-141155	0.379624808	0.000243405	0.047340662
ENSG00000105676	ARMC6	piR-hsa-141155	0.440040513	1.59643943637177e-05	0.007306009
ENSG00000105676	ARMC6	piR-hsa-2499988	0.384737488	0.000197132	0.041722279
ENSG00000105700	KXD1	piR-hsa-141155	0.497065058	7.2353173520042e-07	0.00070271
ENSG00000105993	DNAJB6	piR-hsa-141155	0.415550609	5.13048898884959e-05	0.017116643
ENSG00000106268	NUDT1	piR-hsa-141155	0.422514063	3.71497415983498e-05	0.013363205
ENSG00000107833	NPM3	piR-hsa-2851799	0.493890759	8.72533576199956e-07	0.000822347
ENSG00000107833	NPM3	piR-hsa-141155	0.497222738	7.16797478443331e-07	0.00070271
ENSG00000107859	PITX3	piR-hsa-141155	0.38870627	0.000166968	0.037042332
ENSG00000107984	DKK1	piR-hsa-2851799	0.497988772	6.84917173125909e-07	0.000680325
ENSG00000108010	GLRX3	piR-hsa-2851799	0.4694827	3.4638523193198e-06	0.002365432
ENSG00000108518	PFN1	piR-hsa-2851799	0.538326679	5.29440252220027e-08	0.000125077
ENSG00000109107	ALDOC	piR-hsa-141155	0.379758815	0.000242074	0.047231537
ENSG00000109163	GNRHR	piR-hsa-1288731	0.415619607	5.11428135129735e-05	0.017116643
ENSG00000110104	CCDC86	piR-hsa-141155	0.428741475	2.76654420713375e-05	0.010942246
ENSG00000110880	CORO1C	piR-hsa-141155	0.391753337	0.00014677	0.03421109
ENSG00000111012	CYP27B1	piR-hsa-1288731	0.391816383	0.000146377	0.03421109
ENSG00000111640	GAPDH	piR-hsa-141155	0.413218567	5.70725854376015e-05	0.018200164

ENSG00000112893	MAN2A1	piR-hsa-1288731	0.427813163	2.89188362756179e-05	0.011284801
ENSG00000113430	IRX4	piR-hsa-2499988	0.457476368	6.57068451177611e-06	0.003658239
ENSG00000113430	IRX4	piR-hsa-141155	0.511084589	3.09244513021797e-07	0.000375431
ENSG00000113430	IRX4	piR-hsa-2851799	0.644631858	9.30050761461948e-12	3.87122557425661e-08
ENSG00000120820	GLT8D2	piR-hsa-100956	-0.38415066	0.000201997	0.042341762
ENSG00000120949	TNFRSF8	piR-hsa-2851799	0.470053108	3.35804148864401e-06	0.002311232
ENSG00000120963	ZNF706	piR-hsa-141155	0.437574181	1.803021454274e-05	0.007880105
ENSG00000121297	TSHZ3	piR-hsa-141155	0.390015072	0.000157997	0.035778478
ENSG00000121933	TMIGD3	piR-hsa-100956	-0.383653953	0.0002062	0.04307461
ENSG00000122378	PRXL2A	piR-hsa-141155	0.418714506	4.43443509327819e-05	0.01532071
ENSG00000122378	PRXL2A	piR-hsa-2499988	0.441753391	1.46624636147839e-05	0.006965467
ENSG00000122641	INHBA	piR-hsa-2851799	0.600724244	4.86564619687877e-10	1.84915710464858e-06
ENSG00000122642	FKBP9	piR-hsa-141155	0.403206019	8.93816489157786e-05	0.02456871
ENSG00000122863	CHST3	piR-hsa-141155	0.404616597	8.39796688368117e-05	0.023679558
ENSG00000122965	RBM19	piR-hsa-141155	0.384245229	0.000201205	0.042303645
ENSG00000123159	GIPC1	piR-hsa-2499988	0.391448103	0.000148686	0.034565608
ENSG00000124120	TTPAL	piR-hsa-141155	0.433949774	2.15240523167222e-05	0.009177646
ENSG00000124151	NCOA3	piR-hsa-2499988	0.394915561	0.000128217	0.031839442
ENSG00000124479	NDP	piR-hsa-1288731	0.51484174	2.44644017198635e-07	0.000339434
ENSG00000124575	H1-3	piR-hsa-1288731	0.516692263	2.1775065644612e-07	0.000312026
ENSG00000125520	SLC2A4RG	piR-hsa-141155	0.37894854	0.000250225	0.048282951
ENSG00000126001	CEP250	piR-hsa-141155	0.383621451	0.000206478	0.04307461
ENSG00000126562	WNK4	piR-hsa-2851799	0.402669798	9.15184625246929e-05	0.024920962
ENSG00000126777	KTN1	piR-hsa-141155	0.424146271	3.44068389645237e-05	0.01258369
ENSG00000126777	KTN1	piR-hsa-2851799	0.396986179	0.000117272	0.029763519
ENSG00000126822	PLEKHG3	piR-hsa-141155	0.378353328	0.000256373	0.048973898
ENSG00000127083	OMD	piR-hsa-1288731	0.500150468	6.02034219044082e-07	0.000626474
ENSG00000127507	ADGRE2	piR-hsa-2851799	0.519972442	1.76836365521604e-07	0.00028032
ENSG00000127527	EPS15L1	piR-hsa-141155	0.386609436	0.000182328	0.03974378
ENSG00000127564	PKMYT1	piR-hsa-141155	0.404524903	8.43213964601336e-05	0.023699464
ENSG00000128050	PAICS	piR-hsa-141155	0.401097424	9.8060698083878e-05	0.026373802
ENSG00000128228	SDF2L1	piR-hsa-2851799	0.809557166	7.83992669626444e-22	1.14214665420079e-17

ENSG00000128228	SDF2L1	piR-hsa-141155	0.460675855	5.55313910181648e-06	0.00337632
ENSG00000128228	SDF2L1	piR-hsa-2499988	0.471918653	3.0328542831963e-06	0.002155299
ENSG00000128422	KRT17	piR-hsa-2499988	0.390680351	0.000153609	0.035241441
ENSG00000128510	CPA4	piR-hsa-2851799	0.600085231	5.13149739647786e-10	1.86893411427554e-06
ENSG00000129009	ISLR	piR-hsa-100956	-0.407652727	7.33647576691065e-05	0.021519508
ENSG00000129194	SOX15	piR-hsa-2499988	0.414726723	5.3277519298476e-05	0.0173921
ENSG00000129194	SOX15	piR-hsa-141155	0.405146675	8.20292379065036e-05	0.023204452
ENSG00000129673	AANAT	piR-hsa-141155	0.39927898	0.000106167	0.028036323
ENSG00000130159	ECSIT	piR-hsa-141155	0.396826515	0.000118085	0.029831698
ENSG00000130299	GTPBP3	piR-hsa-141155	0.412048242	6.01892802900747e-05	0.018993303
ENSG00000130309	COLGALT1	piR-hsa-141155	0.424213591	3.42978728641805e-05	0.01258369
ENSG00000131042	LILRB2	piR-hsa-2851799	0.414355788	5.41885014407087e-05	0.017543026
ENSG00000131459	GFPT2	piR-hsa-2851799	0.380341181	0.000236368	0.046325009
ENSG00000131746	TNS4	piR-hsa-2499988	0.438810672	1.6965110693095e-05	0.00760472
ENSG00000131746	TNS4	piR-hsa-141155	0.3890628	0.000164479	0.036767048
ENSG00000131746	TNS4	piR-hsa-2851799	0.382971695	0.000212106	0.043303128
ENSG00000132002	DNAJB1	piR-hsa-141155	0.414767112	5.31791938371094e-05	0.0173921
ENSG00000132382	MYBBP1A	piR-hsa-2851799	0.448513376	1.04337503582901e-05	0.005333416
ENSG00000132382	MYBBP1A	piR-hsa-141155	0.416600941	4.88886559688874e-05	0.016523138
ENSG00000132470	ITGB4	piR-hsa-141155	0.411303155	6.22552472119235e-05	0.019434754
ENSG00000132507	EIF5A	piR-hsa-2851799	0.394340776	0.000131419	0.032087577
ENSG00000132603	NIP7	piR-hsa-141155	0.395521118	0.000124922	0.031109606
ENSG00000132661	NXT1	piR-hsa-141155	0.402832242	9.08661783255909e-05	0.024820665
ENSG00000133101	CCNA1	piR-hsa-141155	0.424919267	3.31748618709665e-05	0.01239237
ENSG00000133597	ADCK2	piR-hsa-141155	0.381031714	0.000229763	0.045518031
ENSG00000133619	KRBA1	piR-hsa-141155	0.382957054	0.000212235	0.043303128
ENSG00000133742	CA1	piR-hsa-1288731	0.530277371	9.06430513676201e-08	0.000193247
ENSG00000134242	PTPN22	piR-hsa-1288731	0.387118856	0.00017848	0.039100197
ENSG00000134294	SLC38A2	piR-hsa-141155	0.440102495	1.59154535770206e-05	0.007306009
ENSG00000134594	RAB33A	piR-hsa-100956	-0.423667339	3.51914257357443e-05	0.012817011
ENSG00000134853	PDGFRA	piR-hsa-100956	-0.411200014	6.2546377462627e-05	0.019456153
ENSG00000134871	COL4A2	piR-hsa-2851799	0.477663671	2.20792302750756e-06	0.001707916

ENSG00000135094	SDS	piR-hsa-2851799	0.438894054	1.68954489961168e-05	0.00760472
ENSG00000135624	CCT7	piR-hsa-141155	0.381476061	0.000225603	0.044920214
ENSG00000135976	ANKRD36	piR-hsa-1288731	0.424697554	3.35239432236066e-05	0.01246948
ENSG00000136040	PLXNC1	piR-hsa-100956	-0.426436417	3.087566155702e-05	0.011941777
ENSG00000136270	TBRG4	piR-hsa-2499988	0.398875201	0.000108049	0.028277085
ENSG00000136270	TBRG4	piR-hsa-141155	0.441276941	1.50142487126639e-05	0.00705589
ENSG00000136997	MYC	piR-hsa-141155	0.408932133	6.92768055539186e-05	0.020667186
ENSG00000137101	CD72	piR-hsa-2851799	0.445401766	1.22137064587455e-05	0.006031639
ENSG00000138031	ADCY3	piR-hsa-141155	0.403482549	8.82978775234749e-05	0.02450196
ENSG00000138172	CALHM2	piR-hsa-2851799	0.381602057	0.000224436	0.044789907
ENSG00000138185	ENTPD1	piR-hsa-100956	-0.406359931	7.77222766896213e-05	0.022421466
ENSG00000138430	OLA1	piR-hsa-2851799	0.397788432	0.00011327	0.029120263
ENSG00000138433	CIR1	piR-hsa-1288731	-0.383197626	0.000210133	0.043303128
ENSG00000139278	GLIPR1	piR-hsa-2851799	0.393551895	0.000135934	0.032732806
ENSG00000139330	KERA	piR-hsa-1288731	0.529578303	9.49138795041378e-08	0.000197534
ENSG00000139547	RDH16	piR-hsa-2499988	0.410290441	6.5169151566244e-05	0.019987493
ENSG00000139547	RDH16	piR-hsa-2851799	0.796718358	9.97729942851927e-21	1.09014467880859e-16
ENSG00000139547	RDH16	piR-hsa-141155	0.464113517	4.62596753013566e-06	0.002995228
ENSG00000140022	STON2	piR-hsa-141155	0.394707965	0.000129365	0.031983671
ENSG00000140511	HAPLN3	piR-hsa-2851799	0.590144738	1.15662095591207e-09	4.04400951025096e-06
ENSG00000140511	HAPLN3	piR-hsa-2499988	0.389169364	0.000163741	0.036699032
ENSG00000140553	UNC45A	piR-hsa-141155	0.389180115	0.000163667	0.036699032
ENSG00000140937	CDH11	piR-hsa-1288731	0.473151858	2.83444168362487e-06	0.002033445
ENSG00000141458	NPC1	piR-hsa-141155	0.400851286	9.91232385071987e-05	0.026577798
ENSG00000141527	CARD14	piR-hsa-2851799	0.37959353	0.000243717	0.047340662
ENSG00000141543	EIF4A3	piR-hsa-2851799	0.480339766	1.90066493784527e-06	0.001496731
ENSG00000141543	EIF4A3	piR-hsa-141155	0.498705365	6.56313415741738e-07	0.000667074
ENSG00000141543	EIF4A3	piR-hsa-2499988	0.460088471	5.72807638097182e-06	0.003406062
ENSG00000141867	BRD4	piR-hsa-141155	0.409853721	6.64644544842492e-05	0.020041338
ENSG00000142512	SIGLEC10	piR-hsa-2851799	0.582902807	2.05538899956761e-09	6.91005970970018e-06
ENSG00000143387	CTSK	piR-hsa-100956	-0.383091365	0.000211059	0.043303128
ENSG00000144583	MARCHF4	piR-hsa-141155	0.454233796	7.7789325403586e-06	0.004197262

ENSG00000144583	MARCHF4	piR-hsa-2499988	0.416205378	4.9785908310417e-05	0.016737639
ENSG00000144583	MARCHF4	piR-hsa-2851799	0.78289242	1.26912861212629e-19	7.92389514185421e-16
ENSG00000144810	COL8A1	piR-hsa-100956	-0.438349599	1.73551931661005e-05	0.007700596
ENSG00000146731	CCT6A	piR-hsa-141155	0.40860854	7.02902515598865e-05	0.020827359
ENSG00000146733	PSPH	piR-hsa-141155	0.470518716	3.27393633695619e-06	0.002286811
ENSG00000146733	PSPH	piR-hsa-2499988	0.413892517	5.53466159634425e-05	0.017786205
ENSG00000147145	LPAR4	piR-hsa-100956	-0.381733369	0.000223226	0.04468291
ENSG00000147650	LRP12	piR-hsa-141155	0.451531885	8.94205561848397e-06	0.004731572
ENSG00000147697	GSDMC	piR-hsa-141155	0.449197927	1.00762955049104e-05	0.005228001
ENSG00000150753	CCT5	piR-hsa-141155	0.440096538	1.59201509059881e-05	0.007306009
ENSG00000152229	PSTPIP2	piR-hsa-2851799	0.391797198	0.000146496	0.03421109
ENSG00000152380	FAM151B	piR-hsa-1288731	0.435331867	2.01230791328185e-05	0.008664819
ENSG00000152578	GRIA4	piR-hsa-1288731	0.429005548	2.73182761041799e-05	0.010867446
ENSG00000152591	DSPP	piR-hsa-1288731	0.43796071	1.76907176735612e-05	0.007809826
ENSG00000154451	GBP5	piR-hsa-2851799	0.517260295	2.10075231736354e-07	0.000306045
ENSG00000154589	LY96	piR-hsa-1288731	0.473128742	2.83804562686235e-06	0.002033445
ENSG00000155034	FBXL18	piR-hsa-2499988	0.385475155	0.00019117	0.041158136
ENSG00000155254	MARVELD1	piR-hsa-2851799	0.405220958	8.17592997027801e-05	0.023203183
ENSG00000155438	NIFK	piR-hsa-141155	0.398058046	0.000111953	0.028952097
ENSG00000155897	ADCY8	piR-hsa-141155	0.448711248	1.03292226617774e-05	0.005311043
ENSG00000156469	MTERF3	piR-hsa-141155	0.413171263	5.71955922852534e-05	0.018200164
ENSG00000156515	HK1	piR-hsa-141155	0.495598642	7.89093567142457e-07	0.000757963
ENSG00000156515	HK1	piR-hsa-2499988	0.407762319	7.30060028304114e-05	0.021486379
ENSG00000156521	TYSND1	piR-hsa-141155	0.382070215	0.000220149	0.044339287
ENSG00000159399	HK2	piR-hsa-141155	0.460514101	5.6008054018513e-06	0.00337632
ENSG00000159840	ZYX	piR-hsa-141155	0.377764806	0.000262588	0.04989738
ENSG00000160349	LCN1	piR-hsa-2851799	0.437605692	1.80023111607676e-05	0.007880105
ENSG00000160767	ENTREP3	piR-hsa-141155	0.404172804	8.56456361773021e-05	0.023994503
ENSG00000160993	ALKBH4	piR-hsa-141155	0.393615343	0.000135566	0.032732806
ENSG00000161036	LRWD1	piR-hsa-2499988	0.383101073	0.000210974	0.043303128
ENSG00000161036	LRWD1	piR-hsa-141155	0.401302361	9.71840875021229e-05	0.026218707
ENSG00000161091	MFSD12	piR-hsa-2851799	0.392007733	0.00014519	0.03421109

ENSG00000161179	YDJC	piR-hsa-2851799	0.882677181	2.84869430208708e-30	1.24502184472716e-25
ENSG00000161179	YDJC	piR-hsa-141155	0.542692672	3.93171858597142e-08	0.00010108
ENSG00000161179	YDJC	piR-hsa-2499988	0.5005664	5.8721717876125e-07	0.000618418
ENSG00000161642	ZNF385A	piR-hsa-141155	0.433277526	2.2237981780383e-05	0.009390444
ENSG00000161642	ZNF385A	piR-hsa-2499988	0.45577376	7.18120120147789e-06	0.003947854
ENSG00000163359	COL6A3	piR-hsa-100956	-0.393961146	0.000133574	0.032353229
ENSG00000163568	AIM2	piR-hsa-1288731	0.377824995	0.000261946	0.049883841
ENSG00000163736	PPBP	piR-hsa-1288731	0.399733414	0.000104085	0.027569817
ENSG00000164051	CCDC51	piR-hsa-141155	0.400511777	0.000100606	0.026810986
ENSG00000164070	HSPA4L	piR-hsa-141155	0.410753349	6.38218278628851e-05	0.019712601
ENSG00000164106	SCRG1	piR-hsa-1288731	0.581668956	2.26375961476655e-09	7.32871214543497e-06
ENSG00000164176	EDIL3	piR-hsa-1288731	0.476872097	2.3074306074292e-06	0.00175449
ENSG00000164251	F2RL1	piR-hsa-141155	0.406420052	7.7514414438399e-05	0.022421466
ENSG00000164251	F2RL1	piR-hsa-2499988	0.382183429	0.000219124	0.044234705
ENSG00000164251	F2RL1	piR-hsa-2851799	0.513512601	2.65874007884757e-07	0.000346866
ENSG00000164465	DCBLD1	piR-hsa-2851799	0.380486401	0.000234965	0.046153434
ENSG00000164620	RELL2	piR-hsa-141155	0.402888876	9.06397827612084e-05	0.024820665
ENSG00000164885	CDK5	piR-hsa-141155	0.379202009	0.000247649	0.047997713
ENSG00000164924	YWHAZ	piR-hsa-141155	0.493722677	8.81181921557215e-07	0.000822347
ENSG00000164932	CTHRC1	piR-hsa-1288731	0.398585782	0.000109417	0.028549649
ENSG00000165071	TMEM71	piR-hsa-1288731	0.428015234	2.86415705674949e-05	0.011277296
ENSG00000166411	IDH3A	piR-hsa-141155	0.39795024	0.000112478	0.029001991
ENSG00000167173	C15orf39	piR-hsa-2499988	0.390071535	0.00015762	0.035778478
ENSG00000167491	GATAD2A	piR-hsa-141155	0.463604502	4.7533970229372e-06	0.003055106
ENSG00000167491	GATAD2A	piR-hsa-2499988	0.401603949	9.59072668399271e-05	0.026034951
ENSG00000167553	TUBA1C	piR-hsa-2499988	0.40779697	7.28929124553822e-05	0.021486379
ENSG00000167693	NXN	piR-hsa-2851799	0.39255567	0.000141841	0.033740713
ENSG00000168003	SLC3A2	piR-hsa-141155	0.432709366	2.28585723174766e-05	0.009469516
ENSG00000168209	DDIT4	piR-hsa-141155	0.438667425	1.70854139435887e-05	0.007619572
ENSG00000168298	H1-4	piR-hsa-1288731	0.520361966	1.72494254148179e-07	0.00028032
ENSG00000168634	WFDC13	piR-hsa-1288731	0.445834786	1.19499980655874e-05	0.005968853
ENSG00000168924	LETM1	piR-hsa-141155	0.524155288	1.35182727289933e-07	0.000255683

ENSG00000168924	LETM1	piR-hsa-2499988	0.381120607	0.000228925	0.045478086
ENSG00000169067	ACTBL2	piR-hsa-2851799	0.412374292	5.93054297672675e-05	0.018782202
ENSG00000169184	MN1	piR-hsa-141155	0.41480315	5.30916018549789e-05	0.0173921
ENSG00000169436	COL22A1	piR-hsa-2851799	0.444497372	1.27821772168585e-05	0.006276911
ENSG00000169548	ZNF280A	piR-hsa-2851799	0.385967297	0.000187286	0.040622038
ENSG00000169635	HIC2	piR-hsa-2851799	0.580125351	2.55299129220834e-09	7.96989174471182e-06
ENSG00000169933	FRMPD4	piR-hsa-141155	0.485285818	1.43604533479185e-06	0.001173128
ENSG00000170191	NANP	piR-hsa-141155	0.429759248	2.63496698220516e-05	0.010676249
ENSG00000170265	ZNF282	piR-hsa-141155	0.417373317	4.71799380694561e-05	0.01618889
ENSG00000170379	TCAF2	piR-hsa-141155	0.396624137	0.000119121	0.02999297
ENSG00000170498	KISS1	piR-hsa-2851799	0.460230178	5.68540442052929e-06	0.003403844
ENSG00000170779	CDCA4	piR-hsa-141155	0.380717543	0.000232747	0.045820729
ENSG00000170891	CYTL1	piR-hsa-1288731	0.393953578	0.000133618	0.032353229
ENSG00000171314	PGAM1	piR-hsa-141155	0.468612135	3.63141492749226e-06	0.002386631
ENSG00000171314	PGAM1	piR-hsa-2499988	0.424467965	3.38890361329693e-05	0.012551867
ENSG00000171603	CLSTN1	piR-hsa-141155	0.409843578	6.64948229666562e-05	0.020041338
ENSG00000172058	SERF1A	piR-hsa-2851799	0.401437679	9.66092580129572e-05	0.02614432
ENSG00000172209	GPR22	piR-hsa-1288731	0.513866977	2.6004881281824e-07	0.000346866
ENSG00000172243	CLEC7A	piR-hsa-1288731	0.390931089	0.000151985	0.035167867
ENSG00000172893	DHCR7	piR-hsa-141155	0.379009088	0.000249607	0.048270318
ENSG00000173065	FAM222B	piR-hsa-1288731	-0.392819211	0.000140256	0.033588369
ENSG00000173432	SAA1	piR-hsa-2851799	0.527961897	1.05533638179937e-07	0.000209652
ENSG00000173546	CSPG4	piR-hsa-2851799	0.425629222	3.20797444898501e-05	0.012191698
ENSG00000174437	ATP2A2	piR-hsa-2499988	0.409913344	6.62862135525357e-05	0.020041338
ENSG00000174469	CNTNAP2	piR-hsa-141155	0.403316299	8.89479685540985e-05	0.024538542
ENSG00000174501	ANKRD36C	piR-hsa-1288731	0.499379471	6.30441007487364e-07	0.000648316
ENSG00000174567	GOLT1A	piR-hsa-141155	-0.383159236	0.000210467	0.043303128
ENSG00000175489	LRRC25	piR-hsa-2851799	0.423071429	3.61909539869359e-05	0.013126354
ENSG00000175592	FOSL1	piR-hsa-141155	0.409624472	6.7153945657024e-05	0.020102488
ENSG00000175602	CCDC85B	piR-hsa-141155	0.440380117	1.56979615978134e-05	0.007298717
ENSG00000175793	SFN	piR-hsa-2499988	0.394612597	0.000129896	0.031983671
ENSG00000176125	UFSP1	piR-hsa-141155	0.451437038	8.98571047780931e-06	0.004731572

ENSG00000176170	SPHK1	piR-hsa-2499988	0.457187269	6.67078718560263e-06	0.003690465
ENSG00000176170	SPHK1	piR-hsa-141155	0.554414684	1.73126271148068e-08	4.8816023745331e-05
ENSG00000176170	SPHK1	piR-hsa-2851799	0.441404854	1.49190362422983e-05	0.007049043
ENSG00000176177	ENTHD1	piR-hsa-1288731	0.416569754	4.89588468055722e-05	0.016523138
ENSG00000176619	LMNB2	piR-hsa-141155	0.38275443	0.00021402	0.043404748
ENSG00000176692	FOXC2	piR-hsa-2851799	0.492303381	9.57512130497377e-07	0.000854042
ENSG00000176853	FAM91A1	piR-hsa-141155	0.39180035	0.000146477	0.03421109
ENSG00000177688	SUMO4	piR-hsa-1288731	0.380988878	0.000230168	0.045518031
ENSG00000177706	FAM20C	piR-hsa-2851799	0.662223477	1.58218272784383e-12	7.27887327583311e-09
ENSG00000178209	PLEC	piR-hsa-141155	0.420488691	4.08369274853472e-05	0.014221338
ENSG00000178342	KCNG2	piR-hsa-2851799	0.601576917	4.53140249591644e-10	1.80040860076389e-06
ENSG00000178802	MPI	piR-hsa-141155	0.425806529	3.18115609433743e-05	0.01214257
ENSG00000179046	TRIML2	piR-hsa-2851799	0.519720494	1.79699946873221e-07	0.00028032
ENSG00000179091	CYC1	piR-hsa-141155	0.390015069	0.000157997	0.035778478
ENSG00000179639	FCER1A	piR-hsa-1288731	0.414571787	5.36562810309439e-05	0.017435299
ENSG00000179826	MRGPRX3	piR-hsa-141155	0.439917839	1.60616736594922e-05	0.007312244
ENSG00000179826	MRGPRX3	piR-hsa-2499988	0.410865947	6.34980432676932e-05	0.019682142
ENSG00000179826	MRGPRX3	piR-hsa-2851799	0.791037218	2.9034393346807e-20	2.20416952843015e-16
ENSG00000179886	TIGD5	piR-hsa-141155	0.417351408	4.72276285649537e-05	0.01618889
ENSG00000179934	CCR8	piR-hsa-100956	-0.388908466	0.000165552	0.036821632
ENSG00000180044	C3orf80	piR-hsa-100956	-0.394675042	0.000129548	0.031983671
ENSG00000180332	KCTD4	piR-hsa-1288731	0.493581455	8.8851084324462e-07	0.000822347
ENSG00000180596	H2BC4	piR-hsa-1288731	0.467341604	3.88971316551518e-06	0.002537312
ENSG00000180921	FAM83H	piR-hsa-141155	0.420145049	4.14954005075509e-05	0.014393305
ENSG00000180957	PITPNB	piR-hsa-2851799	0.420753003	4.03371058822372e-05	0.014129832
ENSG00000180957	PITPNB	piR-hsa-141155	0.428979761	2.73519975568535e-05	0.010867446
ENSG00000181333	HEPHL1	piR-hsa-2851799	0.566632174	7.1151323053187e-09	2.14459901657899e-05
ENSG00000181722	ZBTB20	piR-hsa-1288731	0.481280091	1.80263376919541e-06	0.00144558
ENSG00000181938	GINS3	piR-hsa-141155	0.424244939	3.42472423390907e-05	0.01258369
ENSG00000182782	HCAR2	piR-hsa-2499988	0.446063593	1.18128198885617e-05	0.005934245
ENSG00000182782	HCAR2	piR-hsa-141155	0.444224866	1.29582688390475e-05	0.006327834
ENSG00000182782	HCAR2	piR-hsa-2851799	0.3940817	0.000132886	0.032353229

ENSG00000183054	RGPD6	piR-hsa-2851799	0.475245208	2.52537707980402e-06	0.001892953
ENSG00000183153	GJD3	piR-hsa-2851799	0.502816048	5.12879121552327e-07	0.000557551
ENSG00000183421	RIPK4	piR-hsa-2499988	0.387560997	0.000175202	0.038672828
ENSG00000183454	GRIN2A	piR-hsa-1288731	0.455358366	7.33803721035001e-06	0.004008861
ENSG00000183876	ARSI	piR-hsa-2851799	0.655781118	3.06804797084453e-12	1.3408903656576e-08
ENSG00000183876	ARSI	piR-hsa-141155	0.403305292	8.89911666641261e-05	0.024538542
ENSG00000184162	NR2C2AP	piR-hsa-141155	0.417153238	4.76610607202723e-05	0.016273646
ENSG00000184254	ALDH1A3	piR-hsa-2851799	0.420947921	3.99721706314314e-05	0.014129832
ENSG00000184254	ALDH1A3	piR-hsa-2499988	0.390366058	0.000155668	0.035527205
ENSG00000184260	H2AC20	piR-hsa-1288731	0.480677695	1.86486977449858e-06	0.001481893
ENSG00000184270	H2AC21	piR-hsa-1288731	0.513015697	2.74250892668209e-07	0.000347424
ENSG00000184357	H1-5	piR-hsa-1288731	0.521239417	1.63081133148822e-07	0.000274133
ENSG00000184436	THAP7	piR-hsa-2499988	0.394446321	0.000130826	0.03203216
ENSG00000184436	THAP7	piR-hsa-2851799	0.68979366	7.66584130189257e-14	3.7226177122135e-10
ENSG00000184436	THAP7	piR-hsa-141155	0.473128252	2.8381220572828e-06	0.002033445
ENSG00000185122	HSF1	piR-hsa-141155	0.378330874	0.000256607	0.048973898
ENSG00000185130	H2BC13	piR-hsa-1288731	0.502694256	5.16664039090256e-07	0.000557551
ENSG00000185261	KIAA0825	piR-hsa-1288731	0.498048722	6.82479644298015e-07	0.000680325
ENSG00000185340	GAS2L1	piR-hsa-141155	0.425270632	3.26285862343738e-05	0.012293382
ENSG00000185386	MAPK11	piR-hsa-141155	0.387322093	0.000176967	0.038865972
ENSG00000185651	UBE2L3	piR-hsa-141155	0.514156289	2.55383148910276e-07	0.000346866
ENSG00000185651	UBE2L3	piR-hsa-2499988	0.493383408	8.98886008407237e-07	0.000822347
ENSG00000185651	UBE2L3	piR-hsa-2851799	0.901834679	1.85731019547334e-33	1.62347484186325e-28
ENSG00000185838	GNB1L	piR-hsa-141155	0.446567405	1.15159546033401e-05	0.005818553
ENSG00000185838	GNB1L	piR-hsa-2499988	0.403955603	8.64721286258822e-05	0.024148654
ENSG00000185942	NKAIN3	piR-hsa-1288731	0.549700529	2.4169382241491e-08	6.60201781790228e-05
ENSG00000186340	THBS2	piR-hsa-100956	-0.38436466	0.00020021	0.042271356
ENSG00000186575	NF2	piR-hsa-141155	0.429563293	2.65983642447392e-05	0.010684311
ENSG00000186792	HYAL3	piR-hsa-2499988	0.399825576	0.000103667	0.02754267
ENSG00000186792	HYAL3	piR-hsa-141155	0.42952548	2.6646605691307e-05	0.010684311
ENSG00000187037	GPR141	piR-hsa-100956	-0.382923689	0.000212528	0.043303128
ENSG00000187581	COX8C	piR-hsa-141155	0.392521148	0.00014205	0.033740713

ENSG00000188171	ZNF626	piR-hsa-1288731	0.396946421	0.000117474	0.029763519
ENSG00000188186	LAMTOR4	piR-hsa-141155	0.437081982	1.84713534022059e-05	0.007992975
ENSG00000188394	GPR21	piR-hsa-1288731	0.405915855	7.92737325717504e-05	0.022571065
ENSG00000188522	FAM83G	piR-hsa-2499988	0.385190472	0.000193451	0.041444996
ENSG00000189099	PRSS48	piR-hsa-1288731	0.453001814	8.29044228016228e-06	0.004418705
ENSG00000189410	SH2D5	piR-hsa-2499988	0.433431584	2.20724431301282e-05	0.009365788
ENSG00000189410	SH2D5	piR-hsa-141155	0.502987121	5.07607004730689e-07	0.000557551
ENSG00000196460	RFX8	piR-hsa-1288731	0.438819126	1.69580349854515e-05	0.00760472
ENSG00000196562	SULF2	piR-hsa-2499988	0.470393273	3.29639833763718e-06	0.002286811
ENSG00000196562	SULF2	piR-hsa-141155	0.531002487	8.64065286178373e-08	0.00018882
ENSG00000196747	H2AC13	piR-hsa-1288731	0.409768368	6.67203906073472e-05	0.020041338
ENSG00000196839	ADA	piR-hsa-2851799	0.458717473	6.15676667348444e-06	0.003475529
ENSG00000196839	ADA	piR-hsa-141155	0.416653734	4.87700531558822e-05	0.016523138
ENSG00000196912	ANKRD36B	piR-hsa-1288731	0.489629055	1.11863083497608e-06	0.000977795
ENSG00000197043	ANXA6	piR-hsa-100956	-0.385312186	0.000192473	0.041336752
ENSG00000197061	H4C3	piR-hsa-1288731	0.522593225	1.4950895228183e-07	0.000263187
ENSG00000197153	H3C12	piR-hsa-1288731	0.432913101	2.26341984818153e-05	0.009466293
ENSG00000197238	H4C11	piR-hsa-1288731	0.473836481	2.72964014259145e-06	0.002022016
ENSG00000197457	STMN3	piR-hsa-2851799	0.425829732	3.17766212579156e-05	0.01214257
ENSG00000197714	ZNF460	piR-hsa-1288731	0.492421873	9.50907792657651e-07	0.000854042
ENSG00000198142	SOWAHC	piR-hsa-141155	0.378420573	0.000255671	0.048973898
ENSG00000203737	GPR52	piR-hsa-1288731	0.468884589	3.57817045875659e-06	0.002369454
ENSG00000204291	COL15A1	piR-hsa-2851799	0.469069383	3.54247656771703e-06	0.002369454
ENSG00000204361	NXPE2	piR-hsa-1288731	0.443727415	1.32855965007936e-05	0.006451633
ENSG00000204644	ZFP57	piR-hsa-2499988	0.442819293	1.39032242407483e-05	0.006640879
ENSG00000204644	ZFP57	piR-hsa-141155	0.445632005	1.20728195763976e-05	0.005995938
ENSG00000204644	ZFP57	piR-hsa-2851799	0.877667683	1.5725919973235e-29	4.58200888286824e-25
ENSG00000204961	PCDHA9	piR-hsa-1288731	0.397048215	0.000116958	0.029763519
ENSG00000205323	SARNP	piR-hsa-1288731	0.396568261	0.000119409	0.02999297
ENSG00000206140	TMEM191C	piR-hsa-2851799	0.519452312	1.82796402281568e-07	0.00028032
ENSG00000212126	TAS2R50	piR-hsa-1288731	0.392175695	0.000144156	0.034148144
ENSG00000212128	TAS2R13	piR-hsa-1288731	0.427144374	2.98544770247001e-05	0.011598133

ENSG00000214078	CPNE1	piR-hsa-141155	0.378365661	0.000256244	0.048973898
ENSG00000216937	CCDC7	piR-hsa-1288731	0.403842272	8.69063081274559e-05	0.024192613
ENSG00000221983	UBA52	piR-hsa-141155	0.398922539	0.000107827	0.028277085
ENSG00000222038	POTEJ	piR-hsa-2851799	0.460566666	5.58527315076493e-06	0.00337632
ENSG00000229894	GK3	piR-hsa-1288731	0.526473187	1.16305823736547e-07	0.000225918
ENSG00000236279	CLEC2L	piR-hsa-141155	0.388552951	0.000168049	0.037187815
ENSG00000236444	UBE2L5	piR-hsa-2851799	0.740000655	1.18290215638608e-16	6.89316516598048e-13
ENSG00000242114	MTFP1	piR-hsa-141155	0.556909717	1.44796605364224e-08	4.21889042496227e-05
ENSG00000242372	EIF6	piR-hsa-141155	0.459869424	5.79463061292813e-06	0.003422356
ENSG00000242372	EIF6	piR-hsa-2499988	0.39338978	0.00013688	0.032869915
ENSG00000244005	NFS1	piR-hsa-141155	0.458698239	6.1629900640758e-06	0.003475529
ENSG00000248483	POU5F2	piR-hsa-1288731	0.500886644	5.76044924584476e-07	0.00061405
ENSG00000253276	CCDC71L	piR-hsa-2851799	0.487268154	1.28185841846496e-06	0.001067117
ENSG00000253626	EIF5AL1	piR-hsa-2851799	0.542929558	3.86827991945936e-08	0.00010108
ENSG00000254521	SIGLEC12	piR-hsa-2851799	0.738438422	1.47922717867951e-16	8.0812029805235e-13
ENSG00000254521	SIGLEC12	piR-hsa-141155	0.462513932	5.03761934361414e-06	0.003190857
ENSG00000254858	MPV17L2	piR-hsa-141155	0.415158617	5.22347970407098e-05	0.017294862
ENSG00000254858	MPV17L2	piR-hsa-2499988	0.391911887	0.000145784	0.03421109
ENSG00000255398	HCAR3	piR-hsa-2851799	0.41391057	5.5301057784171e-05	0.017786205
ENSG00000255398	HCAR3	piR-hsa-141155	0.406172898	7.83722368030845e-05	0.022460712
ENSG00000255398	HCAR3	piR-hsa-2499988	0.411639917	6.13134426013105e-05	0.019209348
ENSG00000256188	TAS2R30	piR-hsa-1288731	0.449136479	1.01079067903306e-05	0.005228001
ENSG00000261236	BOP1	piR-hsa-141155	0.46263858	5.00434260207237e-06	0.003190857
ENSG00000262406	MMP12	piR-hsa-141155	0.473616582	2.76289714868841e-06	0.002029452
ENSG00000273703	H2BC14	piR-hsa-1288731	0.502884684	5.10757697331716e-07	0.000557551
ENSG00000274641	H2BC17	piR-hsa-1288731	0.449256552	1.00462234854235e-05	0.005228001
ENSG00000274997	H2AC12	piR-hsa-1288731	0.507557678	3.84351248680806e-07	0.000442055
ENSG00000275379	H3C11	piR-hsa-1288731	0.513683883	2.630432134296e-07	0.000346866
ENSG00000275714	H3C1	piR-hsa-1288731	0.488292678	1.20843831961976e-06	0.001044217
ENSG00000276368	H2AC14	piR-hsa-1288731	0.522024376	1.55075600690972e-07	0.000265787
ENSG00000276410	H2BC3	piR-hsa-1288731	0.523566961	1.40418149147798e-07	0.000255707
ENSG00000276903	H2AC16	piR-hsa-1288731	0.470873938	3.21110965596846e-06	0.002263573

ENSG00000276966	H4C5	piR-hsa-1288731	0.432167508	2.34654868091544e-05	0.009629663
ENSG00000277157	H4C4	piR-hsa-1288731	0.517425812	2.07887487521204e-07	0.000306045
ENSG00000277224	H2BC7	piR-hsa-1288731	0.479811224	1.95795387045626e-06	0.001528078
ENSG00000277775	H3C7	piR-hsa-1288731	0.510210549	3.26438892882987e-07	0.000385595
ENSG00000278463	H2AC4	piR-hsa-1288731	0.512280041	2.87113792129745e-07	0.000358523
ENSG00000278558	TMEM191B	piR-hsa-2851799	0.433154592	2.23709094452215e-05	0.00940116
ENSG00000278588	H2BC10	piR-hsa-1288731	0.52389453	1.37479864392129e-07	0.000255683
ENSG00000278637	H4C1	piR-hsa-1288731	0.506206485	4.1746826026423e-07	0.000473908
ENSG00000278705	H4C2	piR-hsa-1288731	0.522485568	1.50547678014285e-07	0.000263187
ENSG00000286522	H3C2	piR-hsa-1288731	0.509768763	3.35471063395029e-07	0.00039098
ENSG00000287080	H3C3	piR-hsa-1288731	0.520116339	1.75220360537901e-07	0.00028032

Table S7 pathways annotated by five piRNA signature correlated genes

piRNA	Pathway.name	pearson_correlation
piR-hsa-2851799	Signaling by Rho GTPases, Miro GTPases and RHOBTB3	0.383447963
piR-hsa-2851799	Cooperation of Prefoldin and TriC/CCT in actin and tubulin folding	0.383447963
piR-hsa-2851799	Chaperonin-mediated protein folding	0.383447963
piR-hsa-2851799	Prefoldin mediated transfer of substrate to CCT/TriC	0.383447963
piR-hsa-2851799	UCH proteinases	0.383447963
piR-hsa-2851799	Positive epigenetic regulation of rRNA expression	0.383447963
piR-hsa-2851799	Chromatin organization	0.383447963
piR-hsa-2851799	Folding of actin by CCT/TriC	0.383447963
piR-hsa-2851799	Cell junction organization	0.383447963
piR-hsa-2851799	RHO GTPase Effectors	0.383447963
piR-hsa-2851799	Deubiquitination	0.383447963
piR-hsa-2851799	Adherens junctions interactions	0.383447963
piR-hsa-2851799	Signaling by Rho GTPases	0.383447963
piR-hsa-2851799	HATs acetylate histones	0.383447963
piR-hsa-2851799	B-WICH complex positively regulates rRNA expression	0.383447963
piR-hsa-2851799	Chromatin modifying enzymes	0.383447963
piR-hsa-2851799	Protein folding	0.383447963
piR-hsa-2851799	Epigenetic regulation of gene expression	0.383447963
piR-hsa-2499988	Signaling by Nuclear Receptors	0.390366058
piR-hsa-2851799	Signaling by Nuclear Receptors	0.420947921
piR-hsa-141155	Glycolysis	0.379758815
piR-hsa-2499988	Pre-NOTCH Expression and Processing	0.409913344
piR-hsa-2499988	Signaling by NOTCH	0.409913344
piR-hsa-141155	Processing of DNA double-strand break ends	0.424919267
piR-hsa-141155	Cellular Senescence	0.424919267
piR-hsa-141155	Chromosome Maintenance	0.424919267
piR-hsa-141155	DNA Damage/Telomere Stress Induced Senescence	0.424919267
piR-hsa-141155	DNA Replication	0.424919267
piR-hsa-141155	Deubiquitination	0.424919267
piR-hsa-141155	Cell Cycle Checkpoints	0.424919267
piR-hsa-141155	Senescence-Associated Secretory Phenotype (SASP)	0.424919267
piR-hsa-141155	Ub-specific processing proteases	0.424919267
piR-hsa-141155	Telomere Maintenance	0.424919267
piR-hsa-141155	Formation of tubulin folding intermediates by CCT/TriC	0.440096538
piR-hsa-141155	Association of TriC/CCT with target proteins during biosynthesis	0.440096538

piR-hsa-141155	Cooperation of PDCL (PhLP1) and TRiC/CCT in G-protein beta folding	0.440096538
piR-hsa-141155	Prefoldin mediated transfer of substrate to CCT/TriC	0.440096538
piR-hsa-141155	Chaperonin-mediated protein folding	0.440096538
piR-hsa-141155	Folding of actin by CCT/TriC	0.440096538
piR-hsa-141155	Protein folding	0.440096538
piR-hsa-141155	Cooperation of Prefoldin and TriC/CCT in actin and tubulin folding	0.440096538
piR-hsa-141155	Folding of actin by CCT/TriC	0.40860854
piR-hsa-141155	Prefoldin mediated transfer of substrate to CCT/TriC	0.40860854
piR-hsa-141155	Cooperation of Prefoldin and TriC/CCT in actin and tubulin folding	0.40860854
piR-hsa-141155	Chaperonin-mediated protein folding	0.40860854
piR-hsa-141155	Signaling by Rho GTPases	0.40860854
piR-hsa-141155	Signaling by Rho GTPases, Miro GTPases and RHOBTB3	0.40860854
piR-hsa-141155	Cooperation of PDCL (PhLP1) and TRiC/CCT in G-protein beta folding	0.40860854
piR-hsa-141155	Association of TriC/CCT with target proteins during biosynthesis	0.40860854
piR-hsa-141155	Protein folding	0.40860854
piR-hsa-141155	Formation of tubulin folding intermediates by CCT/TriC	0.40860854
piR-hsa-141155	Folding of actin by CCT/TriC	0.381476061
piR-hsa-141155	Cooperation of Prefoldin and TriC/CCT in actin and tubulin folding	0.381476061
piR-hsa-141155	Formation of tubulin folding intermediates by CCT/TriC	0.381476061
piR-hsa-141155	Cooperation of PDCL (PhLP1) and TRiC/CCT in G-protein beta folding	0.381476061
piR-hsa-141155	Chaperonin-mediated protein folding	0.381476061
piR-hsa-141155	Signaling by Rho GTPases	0.381476061
piR-hsa-141155	Prefoldin mediated transfer of substrate to CCT/TriC	0.381476061
piR-hsa-141155	Association of TriC/CCT with target proteins during biosynthesis	0.381476061
piR-hsa-141155	Protein folding	0.381476061
piR-hsa-141155	Signaling by Rho GTPases, Miro GTPases and RHOBTB3	0.381476061
piR-hsa-1288731	Regulation of Homotypic Cell-Cell Adhesion	0.473151858
piR-hsa-1288731	Regulation of CDH11 Expression and Function	0.473151858
piR-hsa-1288731	Regulation of Expression and Function of Type II Classical Cadherins	0.473151858
piR-hsa-1288731	Cell junction organization	0.473151858
piR-hsa-1288731	Regulation of CDH11 mRNA translation by microRNAs	0.473151858
piR-hsa-1288731	Adherens junctions interactions	0.473151858
piR-hsa-1288731	Regulation of CDH11 gene transcription	0.473151858
piR-hsa-141155	Diseases of programmed cell death	0.379202009
piR-hsa-2851799	Collagen formation	0.469069383
piR-hsa-2851799	Assembly of collagen fibrils and other multimeric structures	0.469069383
piR-hsa-2851799	Collagen degradation	0.469069383

piR-hsa-2851799	Collagen formation	0.444497372
piR-hsa-2851799	Collagen formation	0.477663671
piR-hsa-2851799	Collagen degradation	0.477663671
piR-hsa-2851799	Assembly of collagen fibrils and other multimeric structures	0.477663671
piR-hsa-100956	Assembly of collagen fibrils and other multimeric structures	-0.393961146
piR-hsa-100956	Collagen formation	-0.393961146
piR-hsa-100956	Collagen degradation	-0.393961146
piR-hsa-100956	Assembly of collagen fibrils and other multimeric structures	-0.438349599
piR-hsa-100956	Collagen degradation	-0.438349599
piR-hsa-100956	Collagen formation	-0.438349599
piR-hsa-141155	Collagen formation	0.424213591
piR-hsa-100956	Collagen degradation	-0.383091365
piR-hsa-2851799	Signaling by WNT	0.497988772
piR-hsa-2851799	TCF dependent signaling in response to WNT	0.497988772
piR-hsa-141155	Signaling by Nuclear Receptors	0.4072102
piR-hsa-2499988	Maternal to zygotic transition (MZT)	0.460088471
piR-hsa-141155	Maternal to zygotic transition (MZT)	0.498705365
piR-hsa-2851799	Maternal to zygotic transition (MZT)	0.480339766
piR-hsa-141155	Glycolysis	0.381916247
piR-hsa-141155	Signaling by Rho GTPases	0.39180035
piR-hsa-141155	Signaling by Rho GTPases, Miro GTPases and RHOBTB3	0.39180035
piR-hsa-141155	Chaperonin-mediated protein folding	0.403206019
piR-hsa-141155	Association of TriC/CCT with target proteins during biosynthesis	0.403206019
piR-hsa-141155	Protein folding	0.403206019
piR-hsa-141155	Glycolysis	0.413218567
piR-hsa-141155	RNA Polymerase I Transcription	0.463604502
piR-hsa-2499988	RNA Polymerase I Transcription	0.401603949
piR-hsa-141155	HDACs deacetylate histones	0.463604502
piR-hsa-2499988	HDACs deacetylate histones	0.401603949
piR-hsa-141155	Positive epigenetic regulation of rRNA expression	0.463604502
piR-hsa-2499988	Positive epigenetic regulation of rRNA expression	0.401603949
piR-hsa-141155	RNA Polymerase I Promoter Clearance	0.463604502
piR-hsa-2499988	RNA Polymerase I Promoter Clearance	0.401603949
piR-hsa-141155	Chromatin modifying enzymes	0.463604502
piR-hsa-2499988	Chromatin modifying enzymes	0.401603949
piR-hsa-141155	Epigenetic regulation of gene expression	0.463604502
piR-hsa-2499988	Epigenetic regulation of gene expression	0.401603949

piR-hsa-141155	ERCC6 (CSB) and EHMT2 (G9a) positively regulate rRNA expression	0.463604502
piR-hsa-2499988	ERCC6 (CSB) and EHMT2 (G9a) positively regulate rRNA expression	0.401603949
piR-hsa-141155	Chromatin organization	0.463604502
piR-hsa-2499988	Chromatin organization	0.401603949
piR-hsa-141155	DNA Replication	0.424244939
piR-hsa-2499988	Chaperonin-mediated protein folding	0.390440112
piR-hsa-2499988	Protein folding	0.390440112
piR-hsa-2499988	Cooperation of PDCL (PhLP1) and TRiC/CCT in G-protein beta folding	0.390440112
piR-hsa-141155	Signaling by WNT	0.446567405
piR-hsa-2499988	Signaling by WNT	0.403955603
piR-hsa-141155	Signaling by Nuclear Receptors	0.446567405
piR-hsa-2499988	Signaling by Nuclear Receptors	0.403955603
piR-hsa-141155	Cooperation of PDCL (PhLP1) and TRiC/CCT in G-protein beta folding	0.446567405
piR-hsa-2499988	Cooperation of PDCL (PhLP1) and TRiC/CCT in G-protein beta folding	0.403955603
piR-hsa-141155	Chaperonin-mediated protein folding	0.446567405
piR-hsa-2499988	Chaperonin-mediated protein folding	0.403955603
piR-hsa-141155	ESR-mediated signaling	0.446567405
piR-hsa-2499988	ESR-mediated signaling	0.403955603
piR-hsa-141155	Protein folding	0.446567405
piR-hsa-2499988	Protein folding	0.403955603
piR-hsa-141155	Glycolysis	0.45968948
piR-hsa-2499988	Glycolysis	0.390814072
piR-hsa-1288731	Cellular Senescence	0.516692263
piR-hsa-1288731	DNA Damage/Telomere Stress Induced Senescence	0.516692263
piR-hsa-1288731	DNA Damage/Telomere Stress Induced Senescence	0.520361966
piR-hsa-1288731	Cellular Senescence	0.520361966
piR-hsa-1288731	Cellular Senescence	0.521239417
piR-hsa-1288731	DNA Damage/Telomere Stress Induced Senescence	0.521239417
piR-hsa-1288731	Defective pyroptosis	0.507557678
piR-hsa-1288731	Amyloid fiber formation	0.507557678
piR-hsa-1288731	B-WICH complex positively regulates rRNA expression	0.507557678
piR-hsa-1288731	Signaling by Rho GTPases, Miro GTPases and RHOBTB3	0.507557678
piR-hsa-1288731	Nucleosome assembly	0.507557678
piR-hsa-1288731	RNA Polymerase I Transcription	0.507557678
piR-hsa-1288731	Ub-specific processing proteases	0.507557678
piR-hsa-1288731	Meiotic recombination	0.507557678
piR-hsa-1288731	Diseases of programmed cell death	0.507557678

piR-hsa-1288731	Chromosome Maintenance	0.507557678
piR-hsa-1288731	RNA Polymerase I Promoter Clearance	0.507557678
piR-hsa-1288731	Oxidative Stress Induced Senescence	0.507557678
piR-hsa-1288731	HCMV Late Events	0.507557678
piR-hsa-1288731	RNA Polymerase I Promoter Opening	0.507557678
piR-hsa-1288731	Gene Silencing by RNA	0.507557678
piR-hsa-1288731	Signaling by Nuclear Receptors	0.507557678
piR-hsa-1288731	Maternal to zygotic transition (MZT)	0.507557678
piR-hsa-1288731	Deubiquitination	0.507557678
piR-hsa-1288731	Depyrimidination	0.507557678
piR-hsa-1288731	Meiotic synapsis	0.507557678
piR-hsa-1288731	Chromatin modifying enzymes	0.507557678
piR-hsa-1288731	Cleavage of the damaged pyrimidine	0.507557678
piR-hsa-1288731	Pre-NOTCH Expression and Processing	0.507557678
piR-hsa-1288731	Signaling by NOTCH	0.507557678
piR-hsa-1288731	RUNX1 regulates genes involved in megakaryocyte differentiation and platelet function	0.507557678
piR-hsa-1288731	Epigenetic regulation of gene expression	0.507557678
piR-hsa-1288731	Chromatin organization	0.507557678
piR-hsa-1288731	RUNX1 regulates transcription of genes involved in differentiation of HSCs	0.507557678
piR-hsa-1288731	HATs acetylate histones	0.507557678
piR-hsa-1288731	TCF dependent signaling in response to WNT	0.507557678
piR-hsa-1288731	Cleavage of the damaged purine	0.507557678
piR-hsa-1288731	Metalloprotease DUBs	0.507557678
piR-hsa-1288731	DNA Replication	0.507557678
piR-hsa-1288731	Estrogen-dependent gene expression	0.507557678
piR-hsa-1288731	Base Excision Repair	0.507557678
piR-hsa-1288731	Activation of HOX genes during differentiation	0.507557678
piR-hsa-1288731	Transcriptional regulation of granulopoiesis	0.507557678
piR-hsa-1288731	Activation of anterior HOX genes in hindbrain development during early embryogenesis	0.507557678
piR-hsa-1288731	Depurination	0.507557678
piR-hsa-1288731	Signaling by WNT	0.507557678
piR-hsa-1288731	DNA Replication Pre-Initiation	0.507557678
piR-hsa-1288731	DNA Damage/Telomere Stress Induced Senescence	0.507557678
piR-hsa-1288731	UCH proteinases	0.507557678
piR-hsa-1288731	Positive epigenetic regulation of rRNA expression	0.507557678
piR-hsa-1288731	RMTs methylate histone arginines	0.507557678
piR-hsa-1288731	ESR-mediated signaling	0.507557678

piR-hsa-1288731	Mitotic Prophase	0.507557678
piR-hsa-1288731	HDACs deacetylate histones	0.507557678
piR-hsa-1288731	Packaging Of Telomere Ends	0.507557678
piR-hsa-1288731	Deposition of new CENPA-containing nucleosomes at the centromere	0.507557678
piR-hsa-1288731	Meiosis	0.507557678
piR-hsa-1288731	Transcriptional regulation by small RNAs	0.507557678
piR-hsa-1288731	Negative epigenetic regulation of rRNA expression	0.507557678
piR-hsa-1288731	Telomere Maintenance	0.507557678
piR-hsa-1288731	HCMV Infection	0.507557678
piR-hsa-1288731	HCMV Early Events	0.507557678
piR-hsa-1288731	ERCC6 (CSB) and EHMT2 (G9a) positively regulate rRNA expression	0.507557678
piR-hsa-1288731	Chromatin modifications during the maternal to zygotic transition (MZT)	0.507557678
piR-hsa-1288731	Assembly of the ORC complex at the origin of replication	0.507557678
piR-hsa-1288731	Recognition and association of DNA glycosylase with site containing an affected purine	0.507557678
piR-hsa-1288731	PRC2 methylates histones and DNA	0.507557678
piR-hsa-1288731	Cellular Senescence	0.507557678
piR-hsa-1288731	Reproduction	0.507557678
piR-hsa-1288731	Activated PKN1 stimulates transcription of AR (androgen receptor) regulated genes KLK2 and KLK3	0.507557678
piR-hsa-1288731	NoRC negatively regulates rRNA expression	0.507557678
piR-hsa-1288731	Base-Excision Repair, AP Site Formation	0.507557678
piR-hsa-1288731	Assembly of the pre-replicative complex	0.507557678
piR-hsa-1288731	SIRT1 negatively regulates rRNA expression	0.507557678
piR-hsa-1288731	Senescence-Associated Secretory Phenotype (SASP)	0.507557678
piR-hsa-1288731	RHO GTPase Effectors	0.507557678
piR-hsa-1288731	Signaling by Rho GTPases	0.507557678
piR-hsa-1288731	Pre-NOTCH Transcription and Translation	0.507557678
piR-hsa-1288731	Inhibition of DNA recombination at telomere	0.507557678
piR-hsa-1288731	Formation of the beta-catenin:TCF transactivating complex	0.507557678
piR-hsa-1288731	DNA methylation	0.507557678
piR-hsa-1288731	RNA Polymerase I Promoter Escape	0.507557678
piR-hsa-1288731	Recognition and association of DNA glycosylase with site containing an affected pyrimidine	0.507557678
piR-hsa-1288731	Condensation of Prophase Chromosomes	0.507557678
piR-hsa-1288731	RHO GTPases activate PKNs	0.507557678
piR-hsa-1288731	Deubiquitination	0.409768368
piR-hsa-1288731	Ub-specific processing proteases	0.409768368
piR-hsa-1288731	RNA Polymerase I Promoter Clearance	0.409768368
piR-hsa-1288731	RNA Polymerase I Transcription	0.409768368

piR-hsa-1288731	Meiotic recombination	0.409768368
piR-hsa-1288731	Amyloid fiber formation	0.409768368
piR-hsa-1288731	Maternal to zygotic transition (MZT)	0.409768368
piR-hsa-1288731	Depyrimidination	0.409768368
piR-hsa-1288731	B-WICH complex positively regulates rRNA expression	0.409768368
piR-hsa-1288731	HATs acetylate histones	0.409768368
piR-hsa-1288731	Positive epigenetic regulation of rRNA expression	0.409768368
piR-hsa-1288731	Epigenetic regulation of gene expression	0.409768368
piR-hsa-1288731	DNA Replication	0.409768368
piR-hsa-1288731	Signaling by NOTCH	0.409768368
piR-hsa-1288731	Pre-NOTCH Expression and Processing	0.409768368
piR-hsa-1288731	Nucleosome assembly	0.409768368
piR-hsa-1288731	DNA Damage/Telomere Stress Induced Senescence	0.409768368
piR-hsa-1288731	Diseases of programmed cell death	0.409768368
piR-hsa-1288731	Base Excision Repair	0.409768368
piR-hsa-1288731	Depurination	0.409768368
piR-hsa-1288731	HCMV Infection	0.409768368
piR-hsa-1288731	Deposition of new CENPA-containing nucleosomes at the centromere	0.409768368
piR-hsa-1288731	Gene Silencing by RNA	0.409768368
piR-hsa-1288731	Reproduction	0.409768368
piR-hsa-1288731	Signaling by Rho GTPases, Miro GTPases and RHOBTB3	0.409768368
piR-hsa-1288731	HCMV Late Events	0.409768368
piR-hsa-1288731	Signaling by WNT	0.409768368
piR-hsa-1288731	RHO GTPase Effectors	0.409768368
piR-hsa-1288731	DNA Replication Pre-Initiation	0.409768368
piR-hsa-1288731	RUNX1 regulates genes involved in megakaryocyte differentiation and platelet function	0.409768368
piR-hsa-1288731	Chromatin organization	0.409768368
piR-hsa-1288731	Cleavage of the damaged purine	0.409768368
piR-hsa-1288731	SIRT1 negatively regulates rRNA expression	0.409768368
piR-hsa-1288731	Signaling by Nuclear Receptors	0.409768368
piR-hsa-1288731	Oxidative Stress Induced Senescence	0.409768368
piR-hsa-1288731	Condensation of Prophase Chromosomes	0.409768368
piR-hsa-1288731	Metalloprotease DUBs	0.409768368
piR-hsa-1288731	Chromatin modifying enzymes	0.409768368
piR-hsa-1288731	Activation of HOX genes during differentiation	0.409768368
piR-hsa-1288731	Meiotic synapsis	0.409768368
piR-hsa-1288731	Transcriptional regulation by small RNAs	0.409768368

piR-hsa-1288731	TCF dependent signaling in response to WNT	0.409768368
piR-hsa-1288731	Cleavage of the damaged pyrimidine	0.409768368
piR-hsa-1288731	RNA Polymerase I Promoter Opening	0.409768368
piR-hsa-1288731	DNA methylation	0.409768368
piR-hsa-1288731	RUNX1 regulates transcription of genes involved in differentiation of HSCs	0.409768368
piR-hsa-1288731	Defective pyroptosis	0.409768368
piR-hsa-1288731	Activation of anterior HOX genes in hindbrain development during early embryogenesis	0.409768368
piR-hsa-1288731	Signaling by Rho GTPases	0.409768368
piR-hsa-1288731	Base-Excision Repair, AP Site Formation	0.409768368
piR-hsa-1288731	HCMV Early Events	0.409768368
piR-hsa-1288731	Recognition and association of DNA glycosylase with site containing an affected purine	0.409768368
piR-hsa-1288731	Estrogen-dependent gene expression	0.409768368
piR-hsa-1288731	RHO GTPases activate PKNs	0.409768368
piR-hsa-1288731	UCH proteinases	0.409768368
piR-hsa-1288731	Meiosis	0.409768368
piR-hsa-1288731	ESR-mediated signaling	0.409768368
piR-hsa-1288731	Assembly of the ORC complex at the origin of replication	0.409768368
piR-hsa-1288731	Recognition and association of DNA glycosylase with site containing an affected pyrimidine	0.409768368
piR-hsa-1288731	HDACs deacetylate histones	0.409768368
piR-hsa-1288731	PRC2 methylates histones and DNA	0.409768368
piR-hsa-1288731	RNA Polymerase I Promoter Escape	0.409768368
piR-hsa-1288731	Chromosome Maintenance	0.409768368
piR-hsa-1288731	Mitotic Prophase	0.409768368
piR-hsa-1288731	Telomere Maintenance	0.409768368
piR-hsa-1288731	Packaging Of Telomere Ends	0.409768368
piR-hsa-1288731	Inhibition of DNA recombination at telomere	0.409768368
piR-hsa-1288731	NoRC negatively regulates rRNA expression	0.409768368
piR-hsa-1288731	Cellular Senescence	0.409768368
piR-hsa-1288731	Formation of the beta-catenin:TCF transactivating complex	0.409768368
piR-hsa-1288731	Pre-NOTCH Transcription and Translation	0.409768368
piR-hsa-1288731	Negative epigenetic regulation of rRNA expression	0.409768368
piR-hsa-1288731	Senescence-Associated Secretory Phenotype (SASP)	0.409768368
piR-hsa-1288731	RMTs methylate histone arginines	0.409768368
piR-hsa-1288731	Transcriptional regulation of granulopoiesis	0.409768368
piR-hsa-1288731	Activated PKN1 stimulates transcription of AR (androgen receptor) regulated genes KLK2 and KLK3	0.409768368
piR-hsa-1288731	ERCC6 (CSB) and EHMT2 (G9a) positively regulate rRNA expression	0.409768368
piR-hsa-1288731	Assembly of the pre-replicative complex	0.409768368

piR-hsa-1288731	Chromatin modifications during the maternal to zygotic transition (MZT)	0.409768368
piR-hsa-1288731	RNA Polymerase I Transcription	0.522024376
piR-hsa-1288731	DNA Replication	0.522024376
piR-hsa-1288731	Maternal to zygotic transition (MZT)	0.522024376
piR-hsa-1288731	Epigenetic regulation of gene expression	0.522024376
piR-hsa-1288731	RUNX1 regulates genes involved in megakaryocyte differentiation and platelet function	0.522024376
piR-hsa-1288731	Depyrimidination	0.522024376
piR-hsa-1288731	Signaling by Rho GTPases, Miro GTPases and RHOBTB3	0.522024376
piR-hsa-1288731	RNA Polymerase I Promoter Clearance	0.522024376
piR-hsa-1288731	Deubiquitination	0.522024376
piR-hsa-1288731	DNA Damage/Telomere Stress Induced Senescence	0.522024376
piR-hsa-1288731	RHO GTPase Effectors	0.522024376
piR-hsa-1288731	Amyloid fiber formation	0.522024376
piR-hsa-1288731	Base Excision Repair	0.522024376
piR-hsa-1288731	RNA Polymerase I Promoter Opening	0.522024376
piR-hsa-1288731	PRC2 methylates histones and DNA	0.522024376
piR-hsa-1288731	Mitotic Prophase	0.522024376
piR-hsa-1288731	Cleavage of the damaged pyrimidine	0.522024376
piR-hsa-1288731	Chromatin organization	0.522024376
piR-hsa-1288731	Positive epigenetic regulation of rRNA expression	0.522024376
piR-hsa-1288731	Condensation of Prophase Chromosomes	0.522024376
piR-hsa-1288731	Cellular Senescence	0.522024376
piR-hsa-1288731	Oxidative Stress Induced Senescence	0.522024376
piR-hsa-1288731	HCMV Infection	0.522024376
piR-hsa-1288731	Activation of anterior HOX genes in hindbrain development during early embryogenesis	0.522024376
piR-hsa-1288731	SIRT1 negatively regulates rRNA expression	0.522024376
piR-hsa-1288731	Chromatin modifying enzymes	0.522024376
piR-hsa-1288731	Cleavage of the damaged purine	0.522024376
piR-hsa-1288731	Meiotic recombination	0.522024376
piR-hsa-1288731	TCF dependent signaling in response to WNT	0.522024376
piR-hsa-1288731	Assembly of the ORC complex at the origin of replication	0.522024376
piR-hsa-1288731	Defective pyroptosis	0.522024376
piR-hsa-1288731	Recognition and association of DNA glycosylase with site containing an affected purine	0.522024376
piR-hsa-1288731	DNA methylation	0.522024376
piR-hsa-1288731	Diseases of programmed cell death	0.522024376
piR-hsa-1288731	ERCC6 (CSB) and EHMT2 (G9a) positively regulate rRNA expression	0.522024376
piR-hsa-1288731	Reproduction	0.522024376

piR-hsa-1288731	HATs acetylate histones	0.522024376
piR-hsa-1288731	Metalloprotease DUBs	0.522024376
piR-hsa-1288731	HCMV Late Events	0.522024376
piR-hsa-1288731	Depurination	0.522024376
piR-hsa-1288731	Pre-NOTCH Expression and Processing	0.522024376
piR-hsa-1288731	Base-Excision Repair, AP Site Formation	0.522024376
piR-hsa-1288731	Gene Silencing by RNA	0.522024376
piR-hsa-1288731	Nucleosome assembly	0.522024376
piR-hsa-1288731	Recognition and association of DNA glycosylase with site containing an affected pyrimidine	0.522024376
piR-hsa-1288731	B-WICH complex positively regulates rRNA expression	0.522024376
piR-hsa-1288731	RNA Polymerase I Promoter Escape	0.522024376
piR-hsa-1288731	Formation of the beta-catenin:TCF transactivating complex	0.522024376
piR-hsa-1288731	Signaling by Nuclear Receptors	0.522024376
piR-hsa-1288731	Deposition of new CENPA-containing nucleosomes at the centromere	0.522024376
piR-hsa-1288731	Estrogen-dependent gene expression	0.522024376
piR-hsa-1288731	Signaling by NOTCH	0.522024376
piR-hsa-1288731	DNA Replication Pre-Initiation	0.522024376
piR-hsa-1288731	Telomere Maintenance	0.522024376
piR-hsa-1288731	Meiosis	0.522024376
piR-hsa-1288731	Pre-NOTCH Transcription and Translation	0.522024376
piR-hsa-1288731	HDACs deacetylate histones	0.522024376
piR-hsa-1288731	Inhibition of DNA recombination at telomere	0.522024376
piR-hsa-1288731	RUNX1 regulates transcription of genes involved in differentiation of HSCs	0.522024376
piR-hsa-1288731	NoRC negatively regulates rRNA expression	0.522024376
piR-hsa-1288731	Negative epigenetic regulation of rRNA expression	0.522024376
piR-hsa-1288731	Transcriptional regulation by small RNAs	0.522024376
piR-hsa-1288731	Ub-specific processing proteases	0.522024376
piR-hsa-1288731	Activation of HOX genes during differentiation	0.522024376
piR-hsa-1288731	Packaging Of Telomere Ends	0.522024376
piR-hsa-1288731	HCMV Early Events	0.522024376
piR-hsa-1288731	RMTs methylate histone arginines	0.522024376
piR-hsa-1288731	UCH proteinases	0.522024376
piR-hsa-1288731	Transcriptional regulation of granulopoiesis	0.522024376
piR-hsa-1288731	ESR-mediated signaling	0.522024376
piR-hsa-1288731	Signaling by WNT	0.522024376
piR-hsa-1288731	Chromosome Maintenance	0.522024376
piR-hsa-1288731	Meiotic synapsis	0.522024376

piR-hsa-1288731	Chromatin modifications during the maternal to zygotic transition (MZT)	0.522024376
piR-hsa-1288731	Assembly of the pre-replicative complex	0.522024376
piR-hsa-1288731	Activated PKN1 stimulates transcription of AR (androgen receptor) regulated genes KLK2 and KLK3	0.522024376
piR-hsa-1288731	Senescence-Associated Secretory Phenotype (SASP)	0.522024376
piR-hsa-1288731	Signaling by Rho GTPases	0.522024376
piR-hsa-1288731	RHO GTPases activate PKNs	0.522024376
piR-hsa-1288731	HDACs deacetylate histones	0.470873938
piR-hsa-1288731	RUNX1 regulates transcription of genes involved in differentiation of HSCs	0.470873938
piR-hsa-1288731	Cleavage of the damaged purine	0.470873938
piR-hsa-1288731	Signaling by Rho GTPases, Miro GTPases and RHOBTB3	0.470873938
piR-hsa-1288731	Diseases of programmed cell death	0.470873938
piR-hsa-1288731	Depyrimidination	0.470873938
piR-hsa-1288731	HATs acetylate histones	0.470873938
piR-hsa-1288731	Meiotic recombination	0.470873938
piR-hsa-1288731	Meiotic synapsis	0.470873938
piR-hsa-1288731	RMTs methylate histone arginines	0.470873938
piR-hsa-1288731	Assembly of the ORC complex at the origin of replication	0.470873938
piR-hsa-1288731	PRC2 methylates histones and DNA	0.470873938
piR-hsa-1288731	Transcriptional regulation of granulopoiesis	0.470873938
piR-hsa-1288731	Pre-NOTCH Expression and Processing	0.470873938
piR-hsa-1288731	Chromatin modifying enzymes	0.470873938
piR-hsa-1288731	Reproduction	0.470873938
piR-hsa-1288731	Signaling by NOTCH	0.470873938
piR-hsa-1288731	RNA Polymerase I Transcription	0.470873938
piR-hsa-1288731	Maternal to zygotic transition (MZT)	0.470873938
piR-hsa-1288731	Amyloid fiber formation	0.470873938
piR-hsa-1288731	RNA Polymerase I Promoter Clearance	0.470873938
piR-hsa-1288731	Formation of the beta-catenin:TCF transactivating complex	0.470873938
piR-hsa-1288731	Depurination	0.470873938
piR-hsa-1288731	Activated PKN1 stimulates transcription of AR (androgen receptor) regulated genes KLK2 and KLK3	0.470873938
piR-hsa-1288731	ESR-mediated signaling	0.470873938
piR-hsa-1288731	RHO GTPase Effectors	0.470873938
piR-hsa-1288731	Mitotic Prophase	0.470873938
piR-hsa-1288731	RNA Polymerase I Promoter Opening	0.470873938
piR-hsa-1288731	Meiosis	0.470873938
piR-hsa-1288731	HCMV Infection	0.470873938
piR-hsa-1288731	Nucleosome assembly	0.470873938

piR-hsa-1288731	Oxidative Stress Induced Senescence	0.470873938
piR-hsa-1288731	Ub-specific processing proteases	0.470873938
piR-hsa-1288731	Metalloprotease DUBs	0.470873938
piR-hsa-1288731	Chromosome Maintenance	0.470873938
piR-hsa-1288731	Defective pyroptosis	0.470873938
piR-hsa-1288731	SIRT1 negatively regulates rRNA expression	0.470873938
piR-hsa-1288731	Activation of HOX genes during differentiation	0.470873938
piR-hsa-1288731	Chromatin modifications during the maternal to zygotic transition (MZT)	0.470873938
piR-hsa-1288731	Assembly of the pre-replicative complex	0.470873938
piR-hsa-1288731	DNA Damage/Telomere Stress Induced Senescence	0.470873938
piR-hsa-1288731	Signaling by Nuclear Receptors	0.470873938
piR-hsa-1288731	Deubiquitination	0.470873938
piR-hsa-1288731	Gene Silencing by RNA	0.470873938
piR-hsa-1288731	Epigenetic regulation of gene expression	0.470873938
piR-hsa-1288731	Pre-NOTCH Transcription and Translation	0.470873938
piR-hsa-1288731	TCF dependent signaling in response to WNT	0.470873938
piR-hsa-1288731	Positive epigenetic regulation of rRNA expression	0.470873938
piR-hsa-1288731	DNA methylation	0.470873938
piR-hsa-1288731	HCMV Late Events	0.470873938
piR-hsa-1288731	Base-Excision Repair, AP Site Formation	0.470873938
piR-hsa-1288731	Deposition of new CENPA-containing nucleosomes at the centromere	0.470873938
piR-hsa-1288731	Activation of anterior HOX genes in hindbrain development during early embryogenesis	0.470873938
piR-hsa-1288731	Negative epigenetic regulation of rRNA expression	0.470873938
piR-hsa-1288731	B-WICH complex positively regulates rRNA expression	0.470873938
piR-hsa-1288731	Signaling by WNT	0.470873938
piR-hsa-1288731	UCH proteinases	0.470873938
piR-hsa-1288731	RUNX1 regulates genes involved in megakaryocyte differentiation and platelet function	0.470873938
piR-hsa-1288731	Condensation of Prophase Chromosomes	0.470873938
piR-hsa-1288731	Cellular Senescence	0.470873938
piR-hsa-1288731	Estrogen-dependent gene expression	0.470873938
piR-hsa-1288731	RHO GTPases activate PKNs	0.470873938
piR-hsa-1288731	Cleavage of the damaged pyrimidine	0.470873938
piR-hsa-1288731	DNA Replication Pre-Initiation	0.470873938
piR-hsa-1288731	Packaging Of Telomere Ends	0.470873938
piR-hsa-1288731	Telomere Maintenance	0.470873938
piR-hsa-1288731	RNA Polymerase I Promoter Escape	0.470873938
piR-hsa-1288731	Base Excision Repair	0.470873938

piR-hsa-1288731	HCMV Early Events	0.470873938
piR-hsa-1288731	Senescence-Associated Secretory Phenotype (SASP)	0.470873938
piR-hsa-1288731	NoRC negatively regulates rRNA expression	0.470873938
piR-hsa-1288731	Transcriptional regulation by small RNAs	0.470873938
piR-hsa-1288731	Inhibition of DNA recombination at telomere	0.470873938
piR-hsa-1288731	ERCC6 (CSB) and EHMT2 (G9a) positively regulate rRNA expression	0.470873938
piR-hsa-1288731	DNA Replication	0.470873938
piR-hsa-1288731	Chromatin organization	0.470873938
piR-hsa-1288731	Recognition and association of DNA glycosylase with site containing an affected pyrimidine	0.470873938
piR-hsa-1288731	Signaling by Rho GTPases	0.470873938
piR-hsa-1288731	Recognition and association of DNA glycosylase with site containing an affected purine	0.470873938
piR-hsa-1288731	Packaging Of Telomere Ends	0.480677695
piR-hsa-1288731	Epigenetic regulation of gene expression	0.480677695
piR-hsa-1288731	Negative epigenetic regulation of rRNA expression	0.480677695
piR-hsa-1288731	Depyrimidination	0.480677695
piR-hsa-1288731	ESR-mediated signaling	0.480677695
piR-hsa-1288731	UCH proteinases	0.480677695
piR-hsa-1288731	Chromatin organization	0.480677695
piR-hsa-1288731	Senescence-Associated Secretory Phenotype (SASP)	0.480677695
piR-hsa-1288731	Recognition and association of DNA glycosylase with site containing an affected pyrimidine	0.480677695
piR-hsa-1288731	Nucleosome assembly	0.480677695
piR-hsa-1288731	Activated PKN1 stimulates transcription of AR (androgen receptor) regulated genes KLK2 and KLK3	0.480677695
piR-hsa-1288731	RNA Polymerase I Promoter Opening	0.480677695
piR-hsa-1288731	Cellular Senescence	0.480677695
piR-hsa-1288731	RNA Polymerase I Promoter Escape	0.480677695
piR-hsa-1288731	HCMV Infection	0.480677695
piR-hsa-1288731	Recognition and association of DNA glycosylase with site containing an affected purine	0.480677695
piR-hsa-1288731	Chromatin modifications during the maternal to zygotic transition (MZT)	0.480677695
piR-hsa-1288731	Cleavage of the damaged purine	0.480677695
piR-hsa-1288731	Deposition of new CENPA-containing nucleosomes at the centromere	0.480677695
piR-hsa-1288731	Amyloid fiber formation	0.480677695
piR-hsa-1288731	DNA Damage/Telomere Stress Induced Senescence	0.480677695
piR-hsa-1288731	Base Excision Repair	0.480677695
piR-hsa-1288731	ERCC6 (CSB) and EHMT2 (G9a) positively regulate rRNA expression	0.480677695
piR-hsa-1288731	Ub-specific processing proteases	0.480677695
piR-hsa-1288731	DNA Replication	0.480677695
piR-hsa-1288731	Telomere Maintenance	0.480677695

piR-hsa-1288731	SIRT1 negatively regulates rRNA expression	0.480677695
piR-hsa-1288731	RUNX1 regulates transcription of genes involved in differentiation of HSCs	0.480677695
piR-hsa-1288731	Inhibition of DNA recombination at telomere	0.480677695
piR-hsa-1288731	Reproduction	0.480677695
piR-hsa-1288731	Pre-NOTCH Expression and Processing	0.480677695
piR-hsa-1288731	RHO GTPase Effectors	0.480677695
piR-hsa-1288731	Mitotic Prophase	0.480677695
piR-hsa-1288731	HDACs deacetylate histones	0.480677695
piR-hsa-1288731	Activation of HOX genes during differentiation	0.480677695
piR-hsa-1288731	PRC2 methylates histones and DNA	0.480677695
piR-hsa-1288731	RUNX1 regulates genes involved in megakaryocyte differentiation and platelet function	0.480677695
piR-hsa-1288731	Transcriptional regulation by small RNAs	0.480677695
piR-hsa-1288731	Signaling by NOTCH	0.480677695
piR-hsa-1288731	Depurination	0.480677695
piR-hsa-1288731	Assembly of the pre-replicative complex	0.480677695
piR-hsa-1288731	Signaling by Rho GTPases	0.480677695
piR-hsa-1288731	Condensation of Prophase Chromosomes	0.480677695
piR-hsa-1288731	Activation of anterior HOX genes in hindbrain development during early embryogenesis	0.480677695
piR-hsa-1288731	HATs acetylate histones	0.480677695
piR-hsa-1288731	Transcriptional regulation of granulopoiesis	0.480677695
piR-hsa-1288731	HCMV Late Events	0.480677695
piR-hsa-1288731	DNA Replication Pre-Initiation	0.480677695
piR-hsa-1288731	Metalloprotease DUBs	0.480677695
piR-hsa-1288731	Gene Silencing by RNA	0.480677695
piR-hsa-1288731	Meiotic synapsis	0.480677695
piR-hsa-1288731	RNA Polymerase I Promoter Clearance	0.480677695
piR-hsa-1288731	Signaling by WNT	0.480677695
piR-hsa-1288731	B-WICH complex positively regulates rRNA expression	0.480677695
piR-hsa-1288731	Defective pyroptosis	0.480677695
piR-hsa-1288731	Meiotic recombination	0.480677695
piR-hsa-1288731	RHO GTPases activate PKNs	0.480677695
piR-hsa-1288731	NoRC negatively regulates rRNA expression	0.480677695
piR-hsa-1288731	Assembly of the ORC complex at the origin of replication	0.480677695
piR-hsa-1288731	DNA methylation	0.480677695
piR-hsa-1288731	TCF dependent signaling in response to WNT	0.480677695
piR-hsa-1288731	Signaling by Nuclear Receptors	0.480677695
piR-hsa-1288731	RNA Polymerase I Transcription	0.480677695

piR-hsa-1288731	Oxidative Stress Induced Senescence	0.480677695
piR-hsa-1288731	RMTs methylate histone arginines	0.480677695
piR-hsa-1288731	Diseases of programmed cell death	0.480677695
piR-hsa-1288731	Cleavage of the damaged pyrimidine	0.480677695
piR-hsa-1288731	Chromosome Maintenance	0.480677695
piR-hsa-1288731	Chromatin modifying enzymes	0.480677695
piR-hsa-1288731	Pre-NOTCH Transcription and Translation	0.480677695
piR-hsa-1288731	Meiosis	0.480677695
piR-hsa-1288731	Base-Excision Repair, AP Site Formation	0.480677695
piR-hsa-1288731	Deubiquitination	0.480677695
piR-hsa-1288731	Signaling by Rho GTPases, Miro GTPases and RHOBTB3	0.480677695
piR-hsa-1288731	Formation of the beta-catenin:TCF transactivating complex	0.480677695
piR-hsa-1288731	Estrogen-dependent gene expression	0.480677695
piR-hsa-1288731	Positive epigenetic regulation of rRNA expression	0.480677695
piR-hsa-1288731	HCMV Early Events	0.480677695
piR-hsa-1288731	Maternal to zygotic transition (MZT)	0.480677695
piR-hsa-1288731	Ub-specific processing proteases	0.513015697
piR-hsa-1288731	Deubiquitination	0.513015697
piR-hsa-1288731	RMTs methylate histone arginines	0.513015697
piR-hsa-1288731	Chromatin organization	0.513015697
piR-hsa-1288731	HCMV Early Events	0.513015697
piR-hsa-1288731	Chromatin modifying enzymes	0.513015697
piR-hsa-1288731	HCMV Infection	0.513015697
piR-hsa-1288731	HATs acetylate histones	0.513015697
piR-hsa-1288731	HCMV Late Events	0.513015697
piR-hsa-1288731	Metalloprotease DUBs	0.513015697
piR-hsa-1288731	UCH proteinases	0.513015697
piR-hsa-1288731	HDACs deacetylate histones	0.513015697
piR-hsa-1288731	Deubiquitination	0.512280041
piR-hsa-1288731	B-WICH complex positively regulates rRNA expression	0.512280041
piR-hsa-1288731	SIRT1 negatively regulates rRNA expression	0.512280041
piR-hsa-1288731	Recognition and association of DNA glycosylase with site containing an affected pyrimidine	0.512280041
piR-hsa-1288731	Signaling by WNT	0.512280041
piR-hsa-1288731	Positive epigenetic regulation of rRNA expression	0.512280041
piR-hsa-1288731	Signaling by Nuclear Receptors	0.512280041
piR-hsa-1288731	Deposition of new CENPA-containing nucleosomes at the centromere	0.512280041
piR-hsa-1288731	HCMV Late Events	0.512280041

piR-hsa-1288731	Amyloid fiber formation	0.512280041
piR-hsa-1288731	RNA Polymerase I Promoter Clearance	0.512280041
piR-hsa-1288731	Signaling by NOTCH	0.512280041
piR-hsa-1288731	DNA Damage/Telomere Stress Induced Senescence	0.512280041
piR-hsa-1288731	Cleavage of the damaged pyrimidine	0.512280041
piR-hsa-1288731	Metalloprotease DUBs	0.512280041
piR-hsa-1288731	Telomere Maintenance	0.512280041
piR-hsa-1288731	Defective pyroptosis	0.512280041
piR-hsa-1288731	RMTs methylate histone arginines	0.512280041
piR-hsa-1288731	Oxidative Stress Induced Senescence	0.512280041
piR-hsa-1288731	Pre-NOTCH Expression and Processing	0.512280041
piR-hsa-1288731	RNA Polymerase I Promoter Opening	0.512280041
piR-hsa-1288731	Nucleosome assembly	0.512280041
piR-hsa-1288731	Meiosis	0.512280041
piR-hsa-1288731	HDACs deacetylate histones	0.512280041
piR-hsa-1288731	Ub-specific processing proteases	0.512280041
piR-hsa-1288731	Activated PKN1 stimulates transcription of AR (androgen receptor) regulated genes KLK2 and KLK3	0.512280041
piR-hsa-1288731	Cellular Senescence	0.512280041
piR-hsa-1288731	Chromatin modifying enzymes	0.512280041
piR-hsa-1288731	Meiotic synapsis	0.512280041
piR-hsa-1288731	ERCC6 (CSB) and EHMT2 (G9a) positively regulate rRNA expression	0.512280041
piR-hsa-1288731	DNA Replication Pre-Initiation	0.512280041
piR-hsa-1288731	Inhibition of DNA recombination at telomere	0.512280041
piR-hsa-1288731	Activation of HOX genes during differentiation	0.512280041
piR-hsa-1288731	HATs acetylate histones	0.512280041
piR-hsa-1288731	Mitotic Prophase	0.512280041
piR-hsa-1288731	Chromatin organization	0.512280041
piR-hsa-1288731	Recognition and association of DNA glycosylase with site containing an affected purine	0.512280041
piR-hsa-1288731	Estrogen-dependent gene expression	0.512280041
piR-hsa-1288731	Chromatin modifications during the maternal to zygotic transition (MZT)	0.512280041
piR-hsa-1288731	RHO GTPase Effectors	0.512280041
piR-hsa-1288731	HCMV Early Events	0.512280041
piR-hsa-1288731	UCH proteinases	0.512280041
piR-hsa-1288731	Depyrimidination	0.512280041
piR-hsa-1288731	Packaging Of Telomere Ends	0.512280041
piR-hsa-1288731	Base Excision Repair	0.512280041
piR-hsa-1288731	DNA Replication	0.512280041

piR-hsa-1288731	Transcriptional regulation of granulopoiesis	0.512280041
piR-hsa-1288731	RUNX1 regulates transcription of genes involved in differentiation of HSCs	0.512280041
piR-hsa-1288731	RNA Polymerase I Transcription	0.512280041
piR-hsa-1288731	Negative epigenetic regulation of rRNA expression	0.512280041
piR-hsa-1288731	ESR-mediated signaling	0.512280041
piR-hsa-1288731	DNA methylation	0.512280041
piR-hsa-1288731	RHO GTPases activate PKNs	0.512280041
piR-hsa-1288731	Depurination	0.512280041
piR-hsa-1288731	Assembly of the ORC complex at the origin of replication	0.512280041
piR-hsa-1288731	Gene Silencing by RNA	0.512280041
piR-hsa-1288731	Formation of the beta-catenin:TCF transactivating complex	0.512280041
piR-hsa-1288731	Assembly of the pre-replicative complex	0.512280041
piR-hsa-1288731	RUNX1 regulates genes involved in megakaryocyte differentiation and platelet function	0.512280041
piR-hsa-1288731	Condensation of Prophase Chromosomes	0.512280041
piR-hsa-1288731	Cleavage of the damaged purine	0.512280041
piR-hsa-1288731	Activation of anterior HOX genes in hindbrain development during early embryogenesis	0.512280041
piR-hsa-1288731	RNA Polymerase I Promoter Escape	0.512280041
piR-hsa-1288731	HCMV Infection	0.512280041
piR-hsa-1288731	Signaling by Rho GTPases, Miro GTPases and RHOBTB3	0.512280041
piR-hsa-1288731	Maternal to zygotic transition (MZT)	0.512280041
piR-hsa-1288731	Chromosome Maintenance	0.512280041
piR-hsa-1288731	PRC2 methylates histones and DNA	0.512280041
piR-hsa-1288731	Senescence-Associated Secretory Phenotype (SASP)	0.512280041
piR-hsa-1288731	Epigenetic regulation of gene expression	0.512280041
piR-hsa-1288731	Signaling by Rho GTPases	0.512280041
piR-hsa-1288731	TCF dependent signaling in response to WNT	0.512280041
piR-hsa-1288731	Pre-NOTCH Transcription and Translation	0.512280041
piR-hsa-1288731	Transcriptional regulation by small RNAs	0.512280041
piR-hsa-1288731	Base-Excision Repair, AP Site Formation	0.512280041
piR-hsa-1288731	Diseases of programmed cell death	0.512280041
piR-hsa-1288731	NoRC negatively regulates rRNA expression	0.512280041
piR-hsa-1288731	Reproduction	0.512280041
piR-hsa-1288731	Meiotic recombination	0.512280041
piR-hsa-1288731	Activated PKN1 stimulates transcription of AR (androgen receptor) regulated genes KLK2 and KLK3	0.52389453
piR-hsa-1288731	Reproduction	0.52389453
piR-hsa-1288731	DNA Replication	0.52389453
piR-hsa-1288731	Recognition and association of DNA glycosylase with site containing an affected pyrimidine	0.52389453

piR-hsa-1288731	Formation of the beta-catenin:TCF transactivating complex	0.52389453
piR-hsa-1288731	Packaging Of Telomere Ends	0.52389453
piR-hsa-1288731	Assembly of the pre-replicative complex	0.52389453
piR-hsa-1288731	ESR-mediated signaling	0.52389453
piR-hsa-1288731	RUNX1 regulates transcription of genes involved in differentiation of HSCs	0.52389453
piR-hsa-1288731	HCMV Infection	0.52389453
piR-hsa-1288731	RNA Polymerase I Promoter Opening	0.52389453
piR-hsa-1288731	Chromosome Maintenance	0.52389453
piR-hsa-1288731	Depyrimidination	0.52389453
piR-hsa-1288731	Inhibition of DNA recombination at telomere	0.52389453
piR-hsa-1288731	Depurination	0.52389453
piR-hsa-1288731	Signaling by NOTCH	0.52389453
piR-hsa-1288731	Chromatin modifications during the maternal to zygotic transition (MZT)	0.52389453
piR-hsa-1288731	Condensation of Prophase Chromosomes	0.52389453
piR-hsa-1288731	Activation of anterior HOX genes in hindbrain development during early embryogenesis	0.52389453
piR-hsa-1288731	Negative epigenetic regulation of rRNA expression	0.52389453
piR-hsa-1288731	Cellular Senescence	0.52389453
piR-hsa-1288731	RUNX1 regulates genes involved in megakaryocyte differentiation and platelet function	0.52389453
piR-hsa-1288731	DNA Damage/Telomere Stress Induced Senescence	0.52389453
piR-hsa-1288731	Chromatin organization	0.52389453
piR-hsa-1288731	Epigenetic regulation of gene expression	0.52389453
piR-hsa-1288731	Nucleosome assembly	0.52389453
piR-hsa-1288731	RHO GTPase Effectors	0.52389453
piR-hsa-1288731	Transcriptional regulation by small RNAs	0.52389453
piR-hsa-1288731	SIRT1 negatively regulates rRNA expression	0.52389453
piR-hsa-1288731	Signaling by Rho GTPases	0.52389453
piR-hsa-1288731	Senescence-Associated Secretory Phenotype (SASP)	0.52389453
piR-hsa-1288731	Processing of DNA double-strand break ends	0.52389453
piR-hsa-1288731	Cleavage of the damaged purine	0.52389453
piR-hsa-1288731	Estrogen-dependent gene expression	0.52389453
piR-hsa-1288731	Mitotic Prophase	0.52389453
piR-hsa-1288731	Signaling by Nuclear Receptors	0.52389453
piR-hsa-1288731	Deubiquitination	0.52389453
piR-hsa-1288731	Base Excision Repair	0.52389453
piR-hsa-1288731	HCMV Early Events	0.52389453
piR-hsa-1288731	Gene Silencing by RNA	0.52389453
piR-hsa-1288731	G2/M DNA damage checkpoint	0.52389453

piR-hsa-1288731	E3 ubiquitin ligases ubiquitinate target proteins	0.52389453
piR-hsa-1288731	HATs acetylate histones	0.52389453
piR-hsa-1288731	Deposition of new CENPA-containing nucleosomes at the centromere	0.52389453
piR-hsa-1288731	RNA Polymerase I Promoter Escape	0.52389453
piR-hsa-1288731	Transcriptional regulation of granulopoiesis	0.52389453
piR-hsa-1288731	Recruitment and ATM-mediated phosphorylation of repair and signaling proteins at DNA double strand breaks	0.52389453
piR-hsa-1288731	Pre-NOTCH Transcription and Translation	0.52389453
piR-hsa-1288731	ERCC6 (CSB) and EHMT2 (G9a) positively regulate rRNA expression	0.52389453
piR-hsa-1288731	Meiosis	0.52389453
piR-hsa-1288731	HDACs deacetylate histones	0.52389453
piR-hsa-1288731	Protein ubiquitination	0.52389453
piR-hsa-1288731	Signaling by Rho GTPases, Miro GTPases and RHOBTB3	0.52389453
piR-hsa-1288731	B-WICH complex positively regulates rRNA expression	0.52389453
piR-hsa-1288731	Meiotic synapsis	0.52389453
piR-hsa-1288731	RNA Polymerase I Promoter Clearance	0.52389453
piR-hsa-1288731	Meiotic recombination	0.52389453
piR-hsa-1288731	Diseases of programmed cell death	0.52389453
piR-hsa-1288731	NoRC negatively regulates rRNA expression	0.52389453
piR-hsa-1288731	Telomere Maintenance	0.52389453
piR-hsa-1288731	Recognition and association of DNA glycosylase with site containing an affected purine	0.52389453
piR-hsa-1288731	DNA Replication Pre-Initiation	0.52389453
piR-hsa-1288731	Base-Excision Repair, AP Site Formation	0.52389453
piR-hsa-1288731	Replacement of protamines by nucleosomes in the male pronucleus	0.52389453
piR-hsa-1288731	Chromatin modifying enzymes	0.52389453
piR-hsa-1288731	RNA Polymerase I Transcription	0.52389453
piR-hsa-1288731	HCMV Late Events	0.52389453
piR-hsa-1288731	Defective pyroptosis	0.52389453
piR-hsa-1288731	PRC2 methylates histones and DNA	0.52389453
piR-hsa-1288731	DNA Double Strand Break Response	0.52389453
piR-hsa-1288731	Oxidative Stress Induced Senescence	0.52389453
piR-hsa-1288731	Signaling by WNT	0.52389453
piR-hsa-1288731	TCF dependent signaling in response to WNT	0.52389453
piR-hsa-1288731	Maternal to zygotic transition (MZT)	0.52389453
piR-hsa-1288731	Pre-NOTCH Expression and Processing	0.52389453
piR-hsa-1288731	Nonhomologous End-Joining (NHEJ)	0.52389453
piR-hsa-1288731	Ub-specific processing proteases	0.52389453
piR-hsa-1288731	Cell Cycle Checkpoints	0.52389453

piR-hsa-1288731	Activation of HOX genes during differentiation	0.52389453
piR-hsa-1288731	Amyloid fiber formation	0.52389453
piR-hsa-1288731	Positive epigenetic regulation of rRNA expression	0.52389453
piR-hsa-1288731	RHO GTPases activate PKNs	0.52389453
piR-hsa-1288731	Assembly of the ORC complex at the origin of replication	0.52389453
piR-hsa-1288731	G2/M Checkpoints	0.52389453
piR-hsa-1288731	Cleavage of the damaged pyrimidine	0.52389453
piR-hsa-1288731	DNA methylation	0.52389453
piR-hsa-1288731	RUNX1 regulates genes involved in megakaryocyte differentiation and platelet function	0.502694256
piR-hsa-1288731	ESR-mediated signaling	0.502694256
piR-hsa-1288731	HCMV Infection	0.502694256
piR-hsa-1288731	Chromosome Maintenance	0.502694256
piR-hsa-1288731	Chromatin organization	0.502694256
piR-hsa-1288731	Activation of anterior HOX genes in hindbrain development during early embryogenesis	0.502694256
piR-hsa-1288731	Reproduction	0.502694256
piR-hsa-1288731	Depyrimidination	0.502694256
piR-hsa-1288731	DNA methylation	0.502694256
piR-hsa-1288731	DNA Double Strand Break Response	0.502694256
piR-hsa-1288731	RNA Polymerase I Promoter Opening	0.502694256
piR-hsa-1288731	DNA Replication	0.502694256
piR-hsa-1288731	Chromatin modifying enzymes	0.502694256
piR-hsa-1288731	Chromatin modifications during the maternal to zygotic transition (MZT)	0.502694256
piR-hsa-1288731	Assembly of the pre-replicative complex	0.502694256
piR-hsa-1288731	Estrogen-dependent gene expression	0.502694256
piR-hsa-1288731	Senescence-Associated Secretory Phenotype (SASP)	0.502694256
piR-hsa-1288731	Meiotic synapsis	0.502694256
piR-hsa-1288731	Epigenetic regulation of gene expression	0.502694256
piR-hsa-1288731	TCF dependent signaling in response to WNT	0.502694256
piR-hsa-1288731	Activation of HOX genes during differentiation	0.502694256
piR-hsa-1288731	RHO GTPase Effectors	0.502694256
piR-hsa-1288731	Pre-NOTCH Expression and Processing	0.502694256
piR-hsa-1288731	RNA Polymerase I Promoter Clearance	0.502694256
piR-hsa-1288731	HCMV Late Events	0.502694256
piR-hsa-1288731	Base Excision Repair	0.502694256
piR-hsa-1288731	Signaling by Rho GTPases	0.502694256
piR-hsa-1288731	Cellular Senescence	0.502694256
piR-hsa-1288731	Mitotic Prophase	0.502694256

piR-hsa-1288731	RHO GTPases activate PKNs	0.502694256
piR-hsa-1288731	DNA Replication Pre-Initiation	0.502694256
piR-hsa-1288731	Transcriptional regulation of granulopoiesis	0.502694256
piR-hsa-1288731	Assembly of the ORC complex at the origin of replication	0.502694256
piR-hsa-1288731	Processing of DNA double-strand break ends	0.502694256
piR-hsa-1288731	HDACs deacetylate histones	0.502694256
piR-hsa-1288731	Protein ubiquitination	0.502694256
piR-hsa-1288731	Telomere Maintenance	0.502694256
piR-hsa-1288731	Condensation of Prophase Chromosomes	0.502694256
piR-hsa-1288731	ERCC6 (CSB) and EHMT2 (G9a) positively regulate rRNA expression	0.502694256
piR-hsa-1288731	Signaling by Nuclear Receptors	0.502694256
piR-hsa-1288731	RNA Polymerase I Promoter Escape	0.502694256
piR-hsa-1288731	NoRC negatively regulates rRNA expression	0.502694256
piR-hsa-1288731	Nucleosome assembly	0.502694256
piR-hsa-1288731	Depurination	0.502694256
piR-hsa-1288731	Recruitment and ATM-mediated phosphorylation of repair and signaling proteins at DNA double strand breaks	0.502694256
piR-hsa-1288731	Defective pyroptosis	0.502694256
piR-hsa-1288731	Transcriptional regulation by small RNAs	0.502694256
piR-hsa-1288731	Deubiquitination	0.502694256
piR-hsa-1288731	Inhibition of DNA recombination at telomere	0.502694256
piR-hsa-1288731	DNA Damage/Telomere Stress Induced Senescence	0.502694256
piR-hsa-1288731	Cleavage of the damaged pyrimidine	0.502694256
piR-hsa-1288731	RUNX1 regulates transcription of genes involved in differentiation of HSCs	0.502694256
piR-hsa-1288731	Cleavage of the damaged purine	0.502694256
piR-hsa-1288731	Meiosis	0.502694256
piR-hsa-1288731	Nonhomologous End-Joining (NHEJ)	0.502694256
piR-hsa-1288731	Pre-NOTCH Transcription and Translation	0.502694256
piR-hsa-1288731	HATs acetylate histones	0.502694256
piR-hsa-1288731	Negative epigenetic regulation of rRNA expression	0.502694256
piR-hsa-1288731	Signaling by Rho GTPases, Miro GTPases and RHOBTB3	0.502694256
piR-hsa-1288731	Signaling by WNT	0.502694256
piR-hsa-1288731	SIRT1 negatively regulates rRNA expression	0.502694256
piR-hsa-1288731	Cell Cycle Checkpoints	0.502694256
piR-hsa-1288731	Formation of the beta-catenin:TCF transactivating complex	0.502694256
piR-hsa-1288731	Signaling by NOTCH	0.502694256
piR-hsa-1288731	B-WICH complex positively regulates rRNA expression	0.502694256
piR-hsa-1288731	Replacement of protamines by nucleosomes in the male pronucleus	0.502694256

piR-hsa-1288731	HCMV Early Events	0.502694256
piR-hsa-1288731	Ub-specific processing proteases	0.502694256
piR-hsa-1288731	PRC2 methylates histones and DNA	0.502694256
piR-hsa-1288731	G2/M DNA damage checkpoint	0.502694256
piR-hsa-1288731	E3 ubiquitin ligases ubiquitinate target proteins	0.502694256
piR-hsa-1288731	Activated PKN1 stimulates transcription of AR (androgen receptor) regulated genes KLK2 and KLK3	0.502694256
piR-hsa-1288731	Deposition of new CENPA-containing nucleosomes at the centromere	0.502694256
piR-hsa-1288731	Meiotic recombination	0.502694256
piR-hsa-1288731	Packaging Of Telomere Ends	0.502694256
piR-hsa-1288731	Base-Excision Repair, AP Site Formation	0.502694256
piR-hsa-1288731	Positive epigenetic regulation of rRNA expression	0.502694256
piR-hsa-1288731	Diseases of programmed cell death	0.502694256
piR-hsa-1288731	G2/M Checkpoints	0.502694256
piR-hsa-1288731	Oxidative Stress Induced Senescence	0.502694256
piR-hsa-1288731	Amyloid fiber formation	0.502694256
piR-hsa-1288731	Recognition and association of DNA glycosylase with site containing an affected purine	0.502694256
piR-hsa-1288731	RNA Polymerase I Transcription	0.502694256
piR-hsa-1288731	Gene Silencing by RNA	0.502694256
piR-hsa-1288731	Recognition and association of DNA glycosylase with site containing an affected pyrimidine	0.502694256
piR-hsa-1288731	Maternal to zygotic transition (MZT)	0.502694256
piR-hsa-1288731	RUNX1 regulates genes involved in megakaryocyte differentiation and platelet function	0.502884684
piR-hsa-1288731	ESR-mediated signaling	0.502884684
piR-hsa-1288731	Reproduction	0.502884684
piR-hsa-1288731	HDACs deacetylate histones	0.502884684
piR-hsa-1288731	Depyrimidination	0.502884684
piR-hsa-1288731	Mitotic Prophase	0.502884684
piR-hsa-1288731	Signaling by Rho GTPases	0.502884684
piR-hsa-1288731	Chromatin organization	0.502884684
piR-hsa-1288731	Activation of anterior HOX genes in hindbrain development during early embryogenesis	0.502884684
piR-hsa-1288731	RNA Polymerase I Promoter Opening	0.502884684
piR-hsa-1288731	Epigenetic regulation of gene expression	0.502884684
piR-hsa-1288731	HCMV Infection	0.502884684
piR-hsa-1288731	RHO GTPase Effectors	0.502884684
piR-hsa-1288731	Inhibition of DNA recombination at telomere	0.502884684
piR-hsa-1288731	Assembly of the pre-replicative complex	0.502884684
piR-hsa-1288731	TCF dependent signaling in response to WNT	0.502884684
piR-hsa-1288731	Cellular Senescence	0.502884684

piR-hsa-1288731	DNA Replication Pre-Initiation	0.502884684
piR-hsa-1288731	RNA Polymerase I Promoter Escape	0.502884684
piR-hsa-1288731	E3 ubiquitin ligases ubiquitinate target proteins	0.502884684
piR-hsa-1288731	RHO GTPases activate PKNs	0.502884684
piR-hsa-1288731	Recruitment and ATM-mediated phosphorylation of repair and signaling proteins at DNA double strand breaks	0.502884684
piR-hsa-1288731	DNA Replication	0.502884684
piR-hsa-1288731	Base Excision Repair	0.502884684
piR-hsa-1288731	HCMV Late Events	0.502884684
piR-hsa-1288731	Estrogen-dependent gene expression	0.502884684
piR-hsa-1288731	Deubiquitination	0.502884684
piR-hsa-1288731	RNA Polymerase I Promoter Clearance	0.502884684
piR-hsa-1288731	Negative epigenetic regulation of rRNA expression	0.502884684
piR-hsa-1288731	Activated PKN1 stimulates transcription of AR (androgen receptor) regulated genes KLK2 and KLK3	0.502884684
piR-hsa-1288731	Recognition and association of DNA glycosylase with site containing an affected purine	0.502884684
piR-hsa-1288731	Condensation of Prophase Chromosomes	0.502884684
piR-hsa-1288731	Positive epigenetic regulation of rRNA expression	0.502884684
piR-hsa-1288731	Defective pyroptosis	0.502884684
piR-hsa-1288731	Meiotic synapsis	0.502884684
piR-hsa-1288731	Pre-NOTCH Expression and Processing	0.502884684
piR-hsa-1288731	ERCC6 (CSB) and EHMT2 (G9a) positively regulate rRNA expression	0.502884684
piR-hsa-1288731	Transcriptional regulation of granulopoiesis	0.502884684
piR-hsa-1288731	Signaling by Nuclear Receptors	0.502884684
piR-hsa-1288731	Chromosome Maintenance	0.502884684
piR-hsa-1288731	Depurination	0.502884684
piR-hsa-1288731	Processing of DNA double-strand break ends	0.502884684
piR-hsa-1288731	SIRT1 negatively regulates rRNA expression	0.502884684
piR-hsa-1288731	Deposition of new CENPA-containing nucleosomes at the centromere	0.502884684
piR-hsa-1288731	B-WICH complex positively regulates rRNA expression	0.502884684
piR-hsa-1288731	Signaling by NOTCH	0.502884684
piR-hsa-1288731	Signaling by Rho GTPases, Miro GTPases and RHOBTB3	0.502884684
piR-hsa-1288731	Activation of HOX genes during differentiation	0.502884684
piR-hsa-1288731	DNA Damage/Telomere Stress Induced Senescence	0.502884684
piR-hsa-1288731	RUNX1 regulates transcription of genes involved in differentiation of HSCs	0.502884684
piR-hsa-1288731	Cleavage of the damaged purine	0.502884684
piR-hsa-1288731	G2/M Checkpoints	0.502884684
piR-hsa-1288731	Meiosis	0.502884684
piR-hsa-1288731	Ub-specific processing proteases	0.502884684

piR-hsa-1288731	Senescence-Associated Secretory Phenotype (SASP)	0.502884684
piR-hsa-1288731	Chromatin modifications during the maternal to zygotic transition (MZT)	0.502884684
piR-hsa-1288731	Transcriptional regulation by small RNAs	0.502884684
piR-hsa-1288731	Telomere Maintenance	0.502884684
piR-hsa-1288731	Recognition and association of DNA glycosylase with site containing an affected pyrimidine	0.502884684
piR-hsa-1288731	Maternal to zygotic transition (MZT)	0.502884684
piR-hsa-1288731	Base-Excision Repair, AP Site Formation	0.502884684
piR-hsa-1288731	Nucleosome assembly	0.502884684
piR-hsa-1288731	Nonhomologous End-Joining (NHEJ)	0.502884684
piR-hsa-1288731	Assembly of the ORC complex at the origin of replication	0.502884684
piR-hsa-1288731	G2/M DNA damage checkpoint	0.502884684
piR-hsa-1288731	Cleavage of the damaged pyrimidine	0.502884684
piR-hsa-1288731	Protein ubiquitination	0.502884684
piR-hsa-1288731	HCMV Early Events	0.502884684
piR-hsa-1288731	PRC2 methylates histones and DNA	0.502884684
piR-hsa-1288731	Oxidative Stress Induced Senescence	0.502884684
piR-hsa-1288731	Meiotic recombination	0.502884684
piR-hsa-1288731	Chromatin modifying enzymes	0.502884684
piR-hsa-1288731	DNA methylation	0.502884684
piR-hsa-1288731	NoRC negatively regulates rRNA expression	0.502884684
piR-hsa-1288731	Pre-NOTCH Transcription and Translation	0.502884684
piR-hsa-1288731	DNA Double Strand Break Response	0.502884684
piR-hsa-1288731	HATs acetylate histones	0.502884684
piR-hsa-1288731	Cell Cycle Checkpoints	0.502884684
piR-hsa-1288731	Formation of the beta-catenin:TCF transactivating complex	0.502884684
piR-hsa-1288731	Packaging Of Telomere Ends	0.502884684
piR-hsa-1288731	Gene Silencing by RNA	0.502884684
piR-hsa-1288731	RNA Polymerase I Transcription	0.502884684
piR-hsa-1288731	Diseases of programmed cell death	0.502884684
piR-hsa-1288731	Signaling by WNT	0.502884684
piR-hsa-1288731	Amyloid fiber formation	0.502884684
piR-hsa-1288731	Replacement of protamines by nucleosomes in the male pronucleus	0.502884684
piR-hsa-1288731	Activation of anterior HOX genes in hindbrain development during early embryogenesis	0.449256552
piR-hsa-1288731	Recognition and association of DNA glycosylase with site containing an affected pyrimidine	0.449256552
piR-hsa-1288731	Signaling by Rho GTPases	0.449256552
piR-hsa-1288731	Epigenetic regulation of gene expression	0.449256552
piR-hsa-1288731	TCF dependent signaling in response to WNT	0.449256552

piR-hsa-1288731	Cell Cycle Checkpoints	0.449256552
piR-hsa-1288731	RUNX1 regulates genes involved in megakaryocyte differentiation and platelet function	0.449256552
piR-hsa-1288731	Signaling by Rho GTPases, Miro GTPases and RHOBTB3	0.449256552
piR-hsa-1288731	Telomere Maintenance	0.449256552
piR-hsa-1288731	Reproduction	0.449256552
piR-hsa-1288731	Meiosis	0.449256552
piR-hsa-1288731	Inhibition of DNA recombination at telomere	0.449256552
piR-hsa-1288731	RNA Polymerase I Promoter Opening	0.449256552
piR-hsa-1288731	Nonhomologous End-Joining (NHEJ)	0.449256552
piR-hsa-1288731	RHO GTPase Effectors	0.449256552
piR-hsa-1288731	Oxidative Stress Induced Senescence	0.449256552
piR-hsa-1288731	E3 ubiquitin ligases ubiquitinate target proteins	0.449256552
piR-hsa-1288731	RNA Polymerase I Transcription	0.449256552
piR-hsa-1288731	Ub-specific processing proteases	0.449256552
piR-hsa-1288731	HCMV Infection	0.449256552
piR-hsa-1288731	DNA Replication	0.449256552
piR-hsa-1288731	DNA Double Strand Break Response	0.449256552
piR-hsa-1288731	Processing of DNA double-strand break ends	0.449256552
piR-hsa-1288731	Nucleosome assembly	0.449256552
piR-hsa-1288731	HATs acetylate histones	0.449256552
piR-hsa-1288731	RNA Polymerase I Promoter Clearance	0.449256552
piR-hsa-1288731	Deposition of new CENPA-containing nucleosomes at the centromere	0.449256552
piR-hsa-1288731	Chromatin modifying enzymes	0.449256552
piR-hsa-1288731	Activation of HOX genes during differentiation	0.449256552
piR-hsa-1288731	Depurination	0.449256552
piR-hsa-1288731	Pre-NOTCH Expression and Processing	0.449256552
piR-hsa-1288731	RNA Polymerase I Promoter Escape	0.449256552
piR-hsa-1288731	Assembly of the ORC complex at the origin of replication	0.449256552
piR-hsa-1288731	G2/M Checkpoints	0.449256552
piR-hsa-1288731	Defective pyroptosis	0.449256552
piR-hsa-1288731	Assembly of the pre-replicative complex	0.449256552
piR-hsa-1288731	Transcriptional regulation by small RNAs	0.449256552
piR-hsa-1288731	Base Excision Repair	0.449256552
piR-hsa-1288731	RHO GTPases activate PKNs	0.449256552
piR-hsa-1288731	ESR-mediated signaling	0.449256552
piR-hsa-1288731	Meiotic synapsis	0.449256552
piR-hsa-1288731	PRC2 methylates histones and DNA	0.449256552

piR-hsa-1288731	DNA methylation	0.449256552
piR-hsa-1288731	Estrogen-dependent gene expression	0.449256552
piR-hsa-1288731	Pre-NOTCH Transcription and Translation	0.449256552
piR-hsa-1288731	Chromatin modifications during the maternal to zygotic transition (MZT)	0.449256552
piR-hsa-1288731	Cleavage of the damaged purine	0.449256552
piR-hsa-1288731	Diseases of programmed cell death	0.449256552
piR-hsa-1288731	Deubiquitination	0.449256552
piR-hsa-1288731	Chromatin organization	0.449256552
piR-hsa-1288731	DNA Replication Pre-Initiation	0.449256552
piR-hsa-1288731	Transcriptional regulation of granulopoiesis	0.449256552
piR-hsa-1288731	Depyrimidination	0.449256552
piR-hsa-1288731	Chromosome Maintenance	0.449256552
piR-hsa-1288731	Senescence-Associated Secretory Phenotype (SASP)	0.449256552
piR-hsa-1288731	G2/M DNA damage checkpoint	0.449256552
piR-hsa-1288731	HCMV Early Events	0.449256552
piR-hsa-1288731	Gene Silencing by RNA	0.449256552
piR-hsa-1288731	ERCC6 (CSB) and EHMT2 (G9a) positively regulate rRNA expression	0.449256552
piR-hsa-1288731	Formation of the beta-catenin:TCF transactivating complex	0.449256552
piR-hsa-1288731	HCMV Late Events	0.449256552
piR-hsa-1288731	SIRT1 negatively regulates rRNA expression	0.449256552
piR-hsa-1288731	Replacement of protamines by nucleosomes in the male pronucleus	0.449256552
piR-hsa-1288731	Maternal to zygotic transition (MZT)	0.449256552
piR-hsa-1288731	RUNX1 regulates transcription of genes involved in differentiation of HSCs	0.449256552
piR-hsa-1288731	Recruitment and ATM-mediated phosphorylation of repair and signaling proteins at DNA double strand breaks	0.449256552
piR-hsa-1288731	Meiotic recombination	0.449256552
piR-hsa-1288731	Mitotic Prophase	0.449256552
piR-hsa-1288731	Positive epigenetic regulation of rRNA expression	0.449256552
piR-hsa-1288731	Signaling by WNT	0.449256552
piR-hsa-1288731	NoRC negatively regulates rRNA expression	0.449256552
piR-hsa-1288731	DNA Damage/Telomere Stress Induced Senescence	0.449256552
piR-hsa-1288731	Activated PKN1 stimulates transcription of AR (androgen receptor) regulated genes KLK2 and KLK3	0.449256552
piR-hsa-1288731	Signaling by Nuclear Receptors	0.449256552
piR-hsa-1288731	Cleavage of the damaged pyrimidine	0.449256552
piR-hsa-1288731	Protein ubiquitination	0.449256552
piR-hsa-1288731	HDACs deacetylate histones	0.449256552
piR-hsa-1288731	Packaging Of Telomere Ends	0.449256552
piR-hsa-1288731	B-WICH complex positively regulates rRNA expression	0.449256552

piR-hsa-1288731	Signaling by NOTCH	0.449256552
piR-hsa-1288731	Negative epigenetic regulation of rRNA expression	0.449256552
piR-hsa-1288731	Cellular Senescence	0.449256552
piR-hsa-1288731	Condensation of Prophase Chromosomes	0.449256552
piR-hsa-1288731	Recognition and association of DNA glycosylase with site containing an affected purine	0.449256552
piR-hsa-1288731	Base-Excision Repair, AP Site Formation	0.449256552
piR-hsa-1288731	Amyloid fiber formation	0.449256552
piR-hsa-1288731	DNA Damage/Telomere Stress Induced Senescence	0.523566961
piR-hsa-1288731	Signaling by WNT	0.523566961
piR-hsa-1288731	Defective pyroptosis	0.523566961
piR-hsa-1288731	Deubiquitination	0.523566961
piR-hsa-1288731	Nonhomologous End-Joining (NHEJ)	0.523566961
piR-hsa-1288731	RNA Polymerase I Promoter Clearance	0.523566961
piR-hsa-1288731	Deposition of new CENPA-containing nucleosomes at the centromere	0.523566961
piR-hsa-1288731	Recognition and association of DNA glycosylase with site containing an affected pyrimidine	0.523566961
piR-hsa-1288731	HATs acetylate histones	0.523566961
piR-hsa-1288731	Telomere Maintenance	0.523566961
piR-hsa-1288731	Replacement of protamines by nucleosomes in the male pronucleus	0.523566961
piR-hsa-1288731	Transcriptional regulation of granulopoiesis	0.523566961
piR-hsa-1288731	RHO GTPases activate PKNs	0.523566961
piR-hsa-1288731	Cell Cycle Checkpoints	0.523566961
piR-hsa-1288731	Cleavage of the damaged pyrimidine	0.523566961
piR-hsa-1288731	Ub-specific processing proteases	0.523566961
piR-hsa-1288731	ESR-mediated signaling	0.523566961
piR-hsa-1288731	Activation of HOX genes during differentiation	0.523566961
piR-hsa-1288731	DNA methylation	0.523566961
piR-hsa-1288731	Positive epigenetic regulation of rRNA expression	0.523566961
piR-hsa-1288731	Oxidative Stress Induced Senescence	0.523566961
piR-hsa-1288731	Recruitment and ATM-mediated phosphorylation of repair and signaling proteins at DNA double strand breaks	0.523566961
piR-hsa-1288731	Inhibition of DNA recombination at telomere	0.523566961
piR-hsa-1288731	RNA Polymerase I Promoter Opening	0.523566961
piR-hsa-1288731	HCMV Late Events	0.523566961
piR-hsa-1288731	Estrogen-dependent gene expression	0.523566961
piR-hsa-1288731	Packaging Of Telomere Ends	0.523566961
piR-hsa-1288731	DNA Double Strand Break Response	0.523566961
piR-hsa-1288731	Pre-NOTCH Expression and Processing	0.523566961
piR-hsa-1288731	Nucleosome assembly	0.523566961

piR-hsa-1288731	HCMV Infection	0.523566961
piR-hsa-1288731	HCMV Early Events	0.523566961
piR-hsa-1288731	Epigenetic regulation of gene expression	0.523566961
piR-hsa-1288731	Chromatin modifying enzymes	0.523566961
piR-hsa-1288731	Amyloid fiber formation	0.523566961
piR-hsa-1288731	Cellular Senescence	0.523566961
piR-hsa-1288731	ERCC6 (CSB) and EHMT2 (G9a) positively regulate rRNA expression	0.523566961
piR-hsa-1288731	Assembly of the ORC complex at the origin of replication	0.523566961
piR-hsa-1288731	Gene Silencing by RNA	0.523566961
piR-hsa-1288731	HDACs deacetylate histones	0.523566961
piR-hsa-1288731	Recognition and association of DNA glycosylase with site containing an affected purine	0.523566961
piR-hsa-1288731	Chromatin modifications during the maternal to zygotic transition (MZT)	0.523566961
piR-hsa-1288731	Chromatin organization	0.523566961
piR-hsa-1288731	Signaling by Rho GTPases	0.523566961
piR-hsa-1288731	Reproduction	0.523566961
piR-hsa-1288731	Meiotic synapsis	0.523566961
piR-hsa-1288731	E3 ubiquitin ligases ubiquitinate target proteins	0.523566961
piR-hsa-1288731	DNA Replication Pre-Initiation	0.523566961
piR-hsa-1288731	TCF dependent signaling in response to WNT	0.523566961
piR-hsa-1288731	Signaling by Rho GTPases, Miro GTPases and RHOBTB3	0.523566961
piR-hsa-1288731	RUNX1 regulates genes involved in megakaryocyte differentiation and platelet function	0.523566961
piR-hsa-1288731	Senescence-Associated Secretory Phenotype (SASP)	0.523566961
piR-hsa-1288731	Activation of anterior HOX genes in hindbrain development during early embryogenesis	0.523566961
piR-hsa-1288731	Meiosis	0.523566961
piR-hsa-1288731	RHO GTPase Effectors	0.523566961
piR-hsa-1288731	RUNX1 regulates transcription of genes involved in differentiation of HSCs	0.523566961
piR-hsa-1288731	RNA Polymerase I Transcription	0.523566961
piR-hsa-1288731	DNA Replication	0.523566961
piR-hsa-1288731	Negative epigenetic regulation of rRNA expression	0.523566961
piR-hsa-1288731	Depurination	0.523566961
piR-hsa-1288731	G2/M DNA damage checkpoint	0.523566961
piR-hsa-1288731	Activated PKN1 stimulates transcription of AR (androgen receptor) regulated genes KLK2 and KLK3	0.523566961
piR-hsa-1288731	Assembly of the pre-replicative complex	0.523566961
piR-hsa-1288731	Cleavage of the damaged purine	0.523566961
piR-hsa-1288731	Depyrimidination	0.523566961
piR-hsa-1288731	RNA Polymerase I Promoter Escape	0.523566961
piR-hsa-1288731	Condensation of Prophase Chromosomes	0.523566961

piR-hsa-1288731	G2/M Checkpoints	0.523566961
piR-hsa-1288731	Transcriptional regulation by small RNAs	0.523566961
piR-hsa-1288731	Formation of the beta-catenin:TCF transactivating complex	0.523566961
piR-hsa-1288731	Signaling by Nuclear Receptors	0.523566961
piR-hsa-1288731	SIRT1 negatively regulates rRNA expression	0.523566961
piR-hsa-1288731	Processing of DNA double-strand break ends	0.523566961
piR-hsa-1288731	Diseases of programmed cell death	0.523566961
piR-hsa-1288731	Mitotic Prophase	0.523566961
piR-hsa-1288731	Pre-NOTCH Transcription and Translation	0.523566961
piR-hsa-1288731	Base Excision Repair	0.523566961
piR-hsa-1288731	Maternal to zygotic transition (MZT)	0.523566961
piR-hsa-1288731	Meiotic recombination	0.523566961
piR-hsa-1288731	PRC2 methylates histones and DNA	0.523566961
piR-hsa-1288731	NoRC negatively regulates rRNA expression	0.523566961
piR-hsa-1288731	Chromosome Maintenance	0.523566961
piR-hsa-1288731	Protein ubiquitination	0.523566961
piR-hsa-1288731	B-WICH complex positively regulates rRNA expression	0.523566961
piR-hsa-1288731	Signaling by NOTCH	0.523566961
piR-hsa-1288731	Base-Excision Repair, AP Site Formation	0.523566961
piR-hsa-1288731	Cleavage of the damaged pyrimidine	0.467341604
piR-hsa-1288731	Signaling by WNT	0.467341604
piR-hsa-1288731	Activation of HOX genes during differentiation	0.467341604
piR-hsa-1288731	DNA Double Strand Break Response	0.467341604
piR-hsa-1288731	Activation of anterior HOX genes in hindbrain development during early embryogenesis	0.467341604
piR-hsa-1288731	DNA Replication	0.467341604
piR-hsa-1288731	RNA Polymerase I Transcription	0.467341604
piR-hsa-1288731	Ub-specific processing proteases	0.467341604
piR-hsa-1288731	RNA Polymerase I Promoter Clearance	0.467341604
piR-hsa-1288731	Amyloid fiber formation	0.467341604
piR-hsa-1288731	Positive epigenetic regulation of rRNA expression	0.467341604
piR-hsa-1288731	Chromatin modifying enzymes	0.467341604
piR-hsa-1288731	Cell Cycle Checkpoints	0.467341604
piR-hsa-1288731	Reproduction	0.467341604
piR-hsa-1288731	Telomere Maintenance	0.467341604
piR-hsa-1288731	ESR-mediated signaling	0.467341604
piR-hsa-1288731	Replacement of protamines by nucleosomes in the male pronucleus	0.467341604
piR-hsa-1288731	Recognition and association of DNA glycosylase with site containing an affected pyrimidine	0.467341604

piR-hsa-1288731	Gene Silencing by RNA	0.467341604
piR-hsa-1288731	HDACs deacetylate histones	0.467341604
piR-hsa-1288731	RHO GTPases activate PKNs	0.467341604
piR-hsa-1288731	Pre-NOTCH Transcription and Translation	0.467341604
piR-hsa-1288731	Deposition of new CENPA-containing nucleosomes at the centromere	0.467341604
piR-hsa-1288731	Inhibition of DNA recombination at telomere	0.467341604
piR-hsa-1288731	Pre-NOTCH Expression and Processing	0.467341604
piR-hsa-1288731	Cellular Senescence	0.467341604
piR-hsa-1288731	RNA Polymerase I Promoter Escape	0.467341604
piR-hsa-1288731	Nucleosome assembly	0.467341604
piR-hsa-1288731	DNA methylation	0.467341604
piR-hsa-1288731	Oxidative Stress Induced Senescence	0.467341604
piR-hsa-1288731	Deubiquitination	0.467341604
piR-hsa-1288731	Maternal to zygotic transition (MZT)	0.467341604
piR-hsa-1288731	RHO GTPase Effectors	0.467341604
piR-hsa-1288731	Epigenetic regulation of gene expression	0.467341604
piR-hsa-1288731	Chromosome Maintenance	0.467341604
piR-hsa-1288731	Transcriptional regulation of granulopoiesis	0.467341604
piR-hsa-1288731	Nonhomologous End-Joining (NHEJ)	0.467341604
piR-hsa-1288731	PRC2 methylates histones and DNA	0.467341604
piR-hsa-1288731	G2/M Checkpoints	0.467341604
piR-hsa-1288731	Defective pyroptosis	0.467341604
piR-hsa-1288731	Senescence-Associated Secretory Phenotype (SASP)	0.467341604
piR-hsa-1288731	RUNX1 regulates transcription of genes involved in differentiation of HSCs	0.467341604
piR-hsa-1288731	DNA Replication Pre-Initiation	0.467341604
piR-hsa-1288731	Formation of the beta-catenin:TCF transactivating complex	0.467341604
piR-hsa-1288731	HCMV Infection	0.467341604
piR-hsa-1288731	Recruitment and ATM-mediated phosphorylation of repair and signaling proteins at DNA double strand breaks	0.467341604
piR-hsa-1288731	Protein ubiquitination	0.467341604
piR-hsa-1288731	Assembly of the pre-replicative complex	0.467341604
piR-hsa-1288731	Signaling by Rho GTPases, Miro GTPases and RHOBTB3	0.467341604
piR-hsa-1288731	E3 ubiquitin ligases ubiquitinate target proteins	0.467341604
piR-hsa-1288731	Cleavage of the damaged purine	0.467341604
piR-hsa-1288731	Chromatin organization	0.467341604
piR-hsa-1288731	HATs acetylate histones	0.467341604
piR-hsa-1288731	HCMV Late Events	0.467341604
piR-hsa-1288731	RNA Polymerase I Promoter Opening	0.467341604

piR-hsa-1288731	Processing of DNA double-strand break ends	0.467341604
piR-hsa-1288731	TCF dependent signaling in response to WNT	0.467341604
piR-hsa-1288731	Activated PKN1 stimulates transcription of AR (androgen receptor) regulated genes KLK2 and KLK3	0.467341604
piR-hsa-1288731	HCMV Early Events	0.467341604
piR-hsa-1288731	Estrogen-dependent gene expression	0.467341604
piR-hsa-1288731	Chromatin modifications during the maternal to zygotic transition (MZT)	0.467341604
piR-hsa-1288731	Meiosis	0.467341604
piR-hsa-1288731	Diseases of programmed cell death	0.467341604
piR-hsa-1288731	Signaling by Nuclear Receptors	0.467341604
piR-hsa-1288731	SIRT1 negatively regulates rRNA expression	0.467341604
piR-hsa-1288731	Signaling by Rho GTPases	0.467341604
piR-hsa-1288731	Depyrimidination	0.467341604
piR-hsa-1288731	RUNX1 regulates genes involved in megakaryocyte differentiation and platelet function	0.467341604
piR-hsa-1288731	G2/M DNA damage checkpoint	0.467341604
piR-hsa-1288731	B-WICH complex positively regulates rRNA expression	0.467341604
piR-hsa-1288731	NoRC negatively regulates rRNA expression	0.467341604
piR-hsa-1288731	Packaging Of Telomere Ends	0.467341604
piR-hsa-1288731	Meiotic synapsis	0.467341604
piR-hsa-1288731	Signaling by NOTCH	0.467341604
piR-hsa-1288731	Transcriptional regulation by small RNAs	0.467341604
piR-hsa-1288731	Base Excision Repair	0.467341604
piR-hsa-1288731	Assembly of the ORC complex at the origin of replication	0.467341604
piR-hsa-1288731	Depurination	0.467341604
piR-hsa-1288731	ERCC6 (CSB) and EHMT2 (G9a) positively regulate rRNA expression	0.467341604
piR-hsa-1288731	Negative epigenetic regulation of rRNA expression	0.467341604
piR-hsa-1288731	Meiotic recombination	0.467341604
piR-hsa-1288731	Mitotic Prophase	0.467341604
piR-hsa-1288731	Base-Excision Repair, AP Site Formation	0.467341604
piR-hsa-1288731	Recognition and association of DNA glycosylase with site containing an affected purine	0.467341604
piR-hsa-1288731	DNA Damage/Telomere Stress Induced Senescence	0.467341604
piR-hsa-1288731	Condensation of Prophase Chromosomes	0.467341604
piR-hsa-1288731	RNA Polymerase I Promoter Clearance	0.479811224
piR-hsa-1288731	Transcriptional regulation by small RNAs	0.479811224
piR-hsa-1288731	Recognition and association of DNA glycosylase with site containing an affected purine	0.479811224
piR-hsa-1288731	DNA Damage/Telomere Stress Induced Senescence	0.479811224
piR-hsa-1288731	Cleavage of the damaged pyrimidine	0.479811224
piR-hsa-1288731	Telomere Maintenance	0.479811224

piR-hsa-1288731	Positive epigenetic regulation of rRNA expression	0.479811224
piR-hsa-1288731	Defective pyroptosis	0.479811224
piR-hsa-1288731	DNA Replication Pre-Initiation	0.479811224
piR-hsa-1288731	Inhibition of DNA recombination at telomere	0.479811224
piR-hsa-1288731	DNA Double Strand Break Response	0.479811224
piR-hsa-1288731	Deubiquitination	0.479811224
piR-hsa-1288731	Signaling by WNT	0.479811224
piR-hsa-1288731	SIRT1 negatively regulates rRNA expression	0.479811224
piR-hsa-1288731	HCMV Late Events	0.479811224
piR-hsa-1288731	Packaging Of Telomere Ends	0.479811224
piR-hsa-1288731	Signaling by NOTCH	0.479811224
piR-hsa-1288731	HATs acetylate histones	0.479811224
piR-hsa-1288731	Meiosis	0.479811224
piR-hsa-1288731	Recognition and association of DNA glycosylase with site containing an affected pyrimidine	0.479811224
piR-hsa-1288731	RUNX1 regulates transcription of genes involved in differentiation of HSCs	0.479811224
piR-hsa-1288731	Replacement of protamines by nucleosomes in the male pronucleus	0.479811224
piR-hsa-1288731	Cell Cycle Checkpoints	0.479811224
piR-hsa-1288731	G2/M DNA damage checkpoint	0.479811224
piR-hsa-1288731	Epigenetic regulation of gene expression	0.479811224
piR-hsa-1288731	Signaling by Nuclear Receptors	0.479811224
piR-hsa-1288731	Estrogen-dependent gene expression	0.479811224
piR-hsa-1288731	Transcriptional regulation of granulopoiesis	0.479811224
piR-hsa-1288731	Recruitment and ATM-mediated phosphorylation of repair and signaling proteins at DNA double strand breaks	0.479811224
piR-hsa-1288731	Chromatin modifying enzymes	0.479811224
piR-hsa-1288731	Pre-NOTCH Expression and Processing	0.479811224
piR-hsa-1288731	Activation of HOX genes during differentiation	0.479811224
piR-hsa-1288731	Negative epigenetic regulation of rRNA expression	0.479811224
piR-hsa-1288731	Base-Excision Repair, AP Site Formation	0.479811224
piR-hsa-1288731	Base Excision Repair	0.479811224
piR-hsa-1288731	RNA Polymerase I Transcription	0.479811224
piR-hsa-1288731	Activated PKN1 stimulates transcription of AR (androgen receptor) regulated genes KLK2 and KLK3	0.479811224
piR-hsa-1288731	Oxidative Stress Induced Senescence	0.479811224
piR-hsa-1288731	Nonhomologous End-Joining (NHEJ)	0.479811224
piR-hsa-1288731	Cellular Senescence	0.479811224
piR-hsa-1288731	Activation of anterior HOX genes in hindbrain development during early embryogenesis	0.479811224
piR-hsa-1288731	Ub-specific processing proteases	0.479811224
piR-hsa-1288731	Amyloid fiber formation	0.479811224

piR-hsa-1288731	Processing of DNA double-strand break ends	0.479811224
piR-hsa-1288731	ESR-mediated signaling	0.479811224
piR-hsa-1288731	G2/M Checkpoints	0.479811224
piR-hsa-1288731	ERCC6 (CSB) and EHMT2 (G9a) positively regulate rRNA expression	0.479811224
piR-hsa-1288731	HDACs deacetylate histones	0.479811224
piR-hsa-1288731	Mitotic Prophase	0.479811224
piR-hsa-1288731	E3 ubiquitin ligases ubiquitinate target proteins	0.479811224
piR-hsa-1288731	Deposition of new CENPA-containing nucleosomes at the centromere	0.479811224
piR-hsa-1288731	Condensation of Prophase Chromosomes	0.479811224
piR-hsa-1288731	Maternal to zygotic transition (MZT)	0.479811224
piR-hsa-1288731	RNA Polymerase I Promoter Escape	0.479811224
piR-hsa-1288731	HCMV Infection	0.479811224
piR-hsa-1288731	RHO GTPase Effectors	0.479811224
piR-hsa-1288731	Chromatin organization	0.479811224
piR-hsa-1288731	Formation of the beta-catenin:TCF transactivating complex	0.479811224
piR-hsa-1288731	B-WICH complex positively regulates rRNA expression	0.479811224
piR-hsa-1288731	Depyrimidination	0.479811224
piR-hsa-1288731	Gene Silencing by RNA	0.479811224
piR-hsa-1288731	RNA Polymerase I Promoter Opening	0.479811224
piR-hsa-1288731	Nucleosome assembly	0.479811224
piR-hsa-1288731	RUNX1 regulates genes involved in megakaryocyte differentiation and platelet function	0.479811224
piR-hsa-1288731	Signaling by Rho GTPases	0.479811224
piR-hsa-1288731	Chromosome Maintenance	0.479811224
piR-hsa-1288731	DNA Replication	0.479811224
piR-hsa-1288731	RHO GTPases activate PKNs	0.479811224
piR-hsa-1288731	Senescence-Associated Secretory Phenotype (SASP)	0.479811224
piR-hsa-1288731	Signaling by Rho GTPases, Miro GTPases and RHOBTB3	0.479811224
piR-hsa-1288731	Cleavage of the damaged purine	0.479811224
piR-hsa-1288731	Meiotic synapsis	0.479811224
piR-hsa-1288731	DNA methylation	0.479811224
piR-hsa-1288731	Diseases of programmed cell death	0.479811224
piR-hsa-1288731	Assembly of the ORC complex at the origin of replication	0.479811224
piR-hsa-1288731	Depurination	0.479811224
piR-hsa-1288731	HCMV Early Events	0.479811224
piR-hsa-1288731	Reproduction	0.479811224
piR-hsa-1288731	Chromatin modifications during the maternal to zygotic transition (MZT)	0.479811224
piR-hsa-1288731	NoRC negatively regulates rRNA expression	0.479811224

piR-hsa-1288731	Assembly of the pre-replicative complex	0.479811224
piR-hsa-1288731	Pre-NOTCH Transcription and Translation	0.479811224
piR-hsa-1288731	TCF dependent signaling in response to WNT	0.479811224
piR-hsa-1288731	Meiotic recombination	0.479811224
piR-hsa-1288731	Protein ubiquitination	0.479811224
piR-hsa-1288731	PRC2 methylates histones and DNA	0.479811224
piR-hsa-1288731	Chromatin modifying enzymes	0.488292678
piR-hsa-1288731	Maternal to zygotic transition (MZT)	0.488292678
piR-hsa-1288731	HCMV Infection	0.488292678
piR-hsa-1288731	Amyloid fiber formation	0.488292678
piR-hsa-1288731	DNA Replication	0.488292678
piR-hsa-1288731	NoRC negatively regulates rRNA expression	0.488292678
piR-hsa-1288731	Chromatin organization	0.488292678
piR-hsa-1288731	Formation of the beta-catenin:TCF transactivating complex	0.488292678
piR-hsa-1288731	RNA Polymerase I Transcription	0.488292678
piR-hsa-1288731	Transcriptional regulation of granulopoiesis	0.488292678
piR-hsa-1288731	RUNX1 regulates genes involved in megakaryocyte differentiation and platelet function	0.488292678
piR-hsa-1288731	Defective pyroptosis	0.488292678
piR-hsa-1288731	Signaling by Rho GTPases	0.488292678
piR-hsa-1288731	Estrogen-dependent gene expression	0.488292678
piR-hsa-1288731	Epigenetic regulation of gene expression	0.488292678
piR-hsa-1288731	ESR-mediated signaling	0.488292678
piR-hsa-1288731	Cellular Senescence	0.488292678
piR-hsa-1288731	Signaling by NOTCH	0.488292678
piR-hsa-1288731	Signaling by WNT	0.488292678
piR-hsa-1288731	Signaling by Rho GTPases, Miro GTPases and RHOBTB3	0.488292678
piR-hsa-1288731	RUNX1 regulates transcription of genes involved in differentiation of HSCs	0.488292678
piR-hsa-1288731	ERCC6 (CSB) and EHMT2 (G9a) positively regulate rRNA expression	0.488292678
piR-hsa-1288731	Activated PKN1 stimulates transcription of AR (androgen receptor) regulated genes KLK2 and KLK3	0.488292678
piR-hsa-1288731	Pre-NOTCH Transcription and Translation	0.488292678
piR-hsa-1288731	RMTs methylate histone arginines	0.488292678
piR-hsa-1288731	Negative epigenetic regulation of rRNA expression	0.488292678
piR-hsa-1288731	Gene Silencing by RNA	0.488292678
piR-hsa-1288731	Mitotic Prophase	0.488292678
piR-hsa-1288731	Condensation of Prophase Chromosomes	0.488292678
piR-hsa-1288731	RHO GTPases activate PKNs	0.488292678
piR-hsa-1288731	Reproduction	0.488292678

piR-hsa-1288731	Activation of anterior HOX genes in hindbrain development during early embryogenesis	0.488292678
piR-hsa-1288731	RHO GTPase Effectors	0.488292678
piR-hsa-1288731	HDACs deacetylate histones	0.488292678
piR-hsa-1288731	Positive epigenetic regulation of rRNA expression	0.488292678
piR-hsa-1288731	DNA Replication Pre-Initiation	0.488292678
piR-hsa-1288731	Signaling by Nuclear Receptors	0.488292678
piR-hsa-1288731	RNA Polymerase I Promoter Clearance	0.488292678
piR-hsa-1288731	Meiotic recombination	0.488292678
piR-hsa-1288731	PRC2 methylates histones and DNA	0.488292678
piR-hsa-1288731	Meiosis	0.488292678
piR-hsa-1288731	HCMV Early Events	0.488292678
piR-hsa-1288731	RNA Polymerase I Promoter Opening	0.488292678
piR-hsa-1288731	B-WICH complex positively regulates rRNA expression	0.488292678
piR-hsa-1288731	Pre-NOTCH Expression and Processing	0.488292678
piR-hsa-1288731	Transcriptional regulation by small RNAs	0.488292678
piR-hsa-1288731	Diseases of programmed cell death	0.488292678
piR-hsa-1288731	HCMV Late Events	0.488292678
piR-hsa-1288731	Assembly of the ORC complex at the origin of replication	0.488292678
piR-hsa-1288731	Activation of HOX genes during differentiation	0.488292678
piR-hsa-1288731	RNA Polymerase I Promoter Escape	0.488292678
piR-hsa-1288731	SIRT1 negatively regulates rRNA expression	0.488292678
piR-hsa-1288731	Chromatin modifications during the maternal to zygotic transition (MZT)	0.488292678
piR-hsa-1288731	Senescence-Associated Secretory Phenotype (SASP)	0.488292678
piR-hsa-1288731	DNA methylation	0.488292678
piR-hsa-1288731	TCF dependent signaling in response to WNT	0.488292678
piR-hsa-1288731	Assembly of the pre-replicative complex	0.488292678
piR-hsa-1288731	HATs acetylate histones	0.488292678
piR-hsa-1288731	Oxidative Stress Induced Senescence	0.488292678
piR-hsa-1288731	RNA Polymerase I Promoter Clearance	0.513683883
piR-hsa-1288731	Epigenetic regulation of gene expression	0.513683883
piR-hsa-1288731	RMTs methylate histone arginines	0.513683883
piR-hsa-1288731	Chromatin modifying enzymes	0.513683883
piR-hsa-1288731	Chromatin organization	0.513683883
piR-hsa-1288731	Estrogen-dependent gene expression	0.513683883
piR-hsa-1288731	ESR-mediated signaling	0.513683883
piR-hsa-1288731	Condensation of Prophase Chromosomes	0.513683883
piR-hsa-1288731	RUNX1 regulates genes involved in megakaryocyte differentiation and platelet function	0.513683883

piR-hsa-1288731	DNA Replication	0.513683883
piR-hsa-1288731	DNA Replication Pre-Initiation	0.513683883
piR-hsa-1288731	Assembly of the ORC complex at the origin of replication	0.513683883
piR-hsa-1288731	Diseases of programmed cell death	0.513683883
piR-hsa-1288731	RNA Polymerase I Transcription	0.513683883
piR-hsa-1288731	Oxidative Stress Induced Senescence	0.513683883
piR-hsa-1288731	Signaling by Rho GTPases, Miro GTPases and RHOBTB3	0.513683883
piR-hsa-1288731	Activated PKN1 stimulates transcription of AR (androgen receptor) regulated genes KLK2 and KLK3	0.513683883
piR-hsa-1288731	Negative epigenetic regulation of rRNA expression	0.513683883
piR-hsa-1288731	HCMV Infection	0.513683883
piR-hsa-1288731	DNA methylation	0.513683883
piR-hsa-1288731	Reproduction	0.513683883
piR-hsa-1288731	Defective pyroptosis	0.513683883
piR-hsa-1288731	Formation of the beta-catenin:TCF transactivating complex	0.513683883
piR-hsa-1288731	Cellular Senescence	0.513683883
piR-hsa-1288731	HATs acetylate histones	0.513683883
piR-hsa-1288731	ERCC6 (CSB) and EHMT2 (G9a) positively regulate rRNA expression	0.513683883
piR-hsa-1288731	HCMV Late Events	0.513683883
piR-hsa-1288731	Maternal to zygotic transition (MZT)	0.513683883
piR-hsa-1288731	Activation of anterior HOX genes in hindbrain development during early embryogenesis	0.513683883
piR-hsa-1288731	HDACs deacetylate histones	0.513683883
piR-hsa-1288731	Signaling by Rho GTPases	0.513683883
piR-hsa-1288731	RHO GTPases activate PKNs	0.513683883
piR-hsa-1288731	Transcriptional regulation of granulopoiesis	0.513683883
piR-hsa-1288731	Signaling by WNT	0.513683883
piR-hsa-1288731	RHO GTPase Effectors	0.513683883
piR-hsa-1288731	HCMV Early Events	0.513683883
piR-hsa-1288731	Meiotic recombination	0.513683883
piR-hsa-1288731	B-WICH complex positively regulates rRNA expression	0.513683883
piR-hsa-1288731	RUNX1 regulates transcription of genes involved in differentiation of HSCs	0.513683883
piR-hsa-1288731	Positive epigenetic regulation of rRNA expression	0.513683883
piR-hsa-1288731	TCF dependent signaling in response to WNT	0.513683883
piR-hsa-1288731	Amyloid fiber formation	0.513683883
piR-hsa-1288731	Signaling by NOTCH	0.513683883
piR-hsa-1288731	RNA Polymerase I Promoter Opening	0.513683883
piR-hsa-1288731	Transcriptional regulation by small RNAs	0.513683883
piR-hsa-1288731	Mitotic Prophase	0.513683883

piR-hsa-1288731	Pre-NOTCH Expression and Processing	0.513683883
piR-hsa-1288731	Gene Silencing by RNA	0.513683883
piR-hsa-1288731	RNA Polymerase I Promoter Escape	0.513683883
piR-hsa-1288731	Activation of HOX genes during differentiation	0.513683883
piR-hsa-1288731	Signaling by Nuclear Receptors	0.513683883
piR-hsa-1288731	Pre-NOTCH Transcription and Translation	0.513683883
piR-hsa-1288731	Chromatin modifications during the maternal to zygotic transition (MZT)	0.513683883
piR-hsa-1288731	Meiosis	0.513683883
piR-hsa-1288731	SIRT1 negatively regulates rRNA expression	0.513683883
piR-hsa-1288731	NoRC negatively regulates rRNA expression	0.513683883
piR-hsa-1288731	Assembly of the pre-replicative complex	0.513683883
piR-hsa-1288731	Senescence-Associated Secretory Phenotype (SASP)	0.513683883
piR-hsa-1288731	PRC2 methylates histones and DNA	0.513683883
piR-hsa-1288731	RNA Polymerase I Transcription	0.432913101
piR-hsa-1288731	RNA Polymerase I Promoter Clearance	0.432913101
piR-hsa-1288731	Defective pyroptosis	0.432913101
piR-hsa-1288731	HCMV Infection	0.432913101
piR-hsa-1288731	Chromatin organization	0.432913101
piR-hsa-1288731	RUNX1 regulates genes involved in megakaryocyte differentiation and platelet function	0.432913101
piR-hsa-1288731	HDACs deacetylate histones	0.432913101
piR-hsa-1288731	Epigenetic regulation of gene expression	0.432913101
piR-hsa-1288731	Chromatin modifying enzymes	0.432913101
piR-hsa-1288731	Signaling by WNT	0.432913101
piR-hsa-1288731	ESR-mediated signaling	0.432913101
piR-hsa-1288731	Negative epigenetic regulation of rRNA expression	0.432913101
piR-hsa-1288731	HATs acetylate histones	0.432913101
piR-hsa-1288731	RUNX1 regulates transcription of genes involved in differentiation of HSCs	0.432913101
piR-hsa-1288731	Estrogen-dependent gene expression	0.432913101
piR-hsa-1288731	Reproduction	0.432913101
piR-hsa-1288731	DNA Replication	0.432913101
piR-hsa-1288731	Amyloid fiber formation	0.432913101
piR-hsa-1288731	Signaling by Rho GTPases	0.432913101
piR-hsa-1288731	Diseases of programmed cell death	0.432913101
piR-hsa-1288731	Condensation of Prophase Chromosomes	0.432913101
piR-hsa-1288731	RMTs methylate histone arginines	0.432913101
piR-hsa-1288731	Maternal to zygotic transition (MZT)	0.432913101
piR-hsa-1288731	Cellular Senescence	0.432913101

piR-hsa-1288731	HCMV Early Events	0.432913101
piR-hsa-1288731	PRC2 methylates histones and DNA	0.432913101
piR-hsa-1288731	Mitotic Prophase	0.432913101
piR-hsa-1288731	ERCC6 (CSB) and EHMT2 (G9a) positively regulate rRNA expression	0.432913101
piR-hsa-1288731	Signaling by Rho GTPases, Miro GTPases and RHOBTB3	0.432913101
piR-hsa-1288731	Oxidative Stress Induced Senescence	0.432913101
piR-hsa-1288731	B-WICH complex positively regulates rRNA expression	0.432913101
piR-hsa-1288731	Pre-NOTCH Transcription and Translation	0.432913101
piR-hsa-1288731	Meiosis	0.432913101
piR-hsa-1288731	Meiotic recombination	0.432913101
piR-hsa-1288731	Formation of the beta-catenin:TCF transactivating complex	0.432913101
piR-hsa-1288731	TCF dependent signaling in response to WNT	0.432913101
piR-hsa-1288731	RHO GTPase Effectors	0.432913101
piR-hsa-1288731	DNA Replication Pre-Initiation	0.432913101
piR-hsa-1288731	Activated PKN1 stimulates transcription of AR (androgen receptor) regulated genes KLK2 and KLK3	0.432913101
piR-hsa-1288731	Pre-NOTCH Expression and Processing	0.432913101
piR-hsa-1288731	HCMV Late Events	0.432913101
piR-hsa-1288731	Activation of anterior HOX genes in hindbrain development during early embryogenesis	0.432913101
piR-hsa-1288731	SIRT1 negatively regulates rRNA expression	0.432913101
piR-hsa-1288731	RNA Polymerase I Promoter Opening	0.432913101
piR-hsa-1288731	Signaling by Nuclear Receptors	0.432913101
piR-hsa-1288731	Positive epigenetic regulation of rRNA expression	0.432913101
piR-hsa-1288731	Transcriptional regulation of granulopoiesis	0.432913101
piR-hsa-1288731	Assembly of the ORC complex at the origin of replication	0.432913101
piR-hsa-1288731	RHO GTPases activate PKNs	0.432913101
piR-hsa-1288731	Gene Silencing by RNA	0.432913101
piR-hsa-1288731	Signaling by NOTCH	0.432913101
piR-hsa-1288731	Transcriptional regulation by small RNAs	0.432913101
piR-hsa-1288731	RNA Polymerase I Promoter Escape	0.432913101
piR-hsa-1288731	NoRC negatively regulates rRNA expression	0.432913101
piR-hsa-1288731	DNA methylation	0.432913101
piR-hsa-1288731	Chromatin modifications during the maternal to zygotic transition (MZT)	0.432913101
piR-hsa-1288731	Senescence-Associated Secretory Phenotype (SASP)	0.432913101
piR-hsa-1288731	Activation of HOX genes during differentiation	0.432913101
piR-hsa-1288731	Assembly of the pre-replicative complex	0.432913101
piR-hsa-1288731	Signaling by NOTCH	0.509768763
piR-hsa-1288731	DNA methylation	0.509768763

piR-hsa-1288731	Chromatin modifications during the maternal to zygotic transition (MZT)	0.509768763
piR-hsa-1288731	Signaling by Rho GTPases, Miro GTPases and RHOBTB3	0.509768763
piR-hsa-1288731	Reproduction	0.509768763
piR-hsa-1288731	RNA Polymerase I Promoter Clearance	0.509768763
piR-hsa-1288731	Chromatin modifying enzymes	0.509768763
piR-hsa-1288731	Senescence-Associated Secretory Phenotype (SASP)	0.509768763
piR-hsa-1288731	Assembly of the ORC complex at the origin of replication	0.509768763
piR-hsa-1288731	Positive epigenetic regulation of rRNA expression	0.509768763
piR-hsa-1288731	Pre-NOTCH Expression and Processing	0.509768763
piR-hsa-1288731	RHO GTPase Effectors	0.509768763
piR-hsa-1288731	HCMV Early Events	0.509768763
piR-hsa-1288731	RNA Polymerase I Transcription	0.509768763
piR-hsa-1288731	TCF dependent signaling in response to WNT	0.509768763
piR-hsa-1288731	Signaling by Nuclear Receptors	0.509768763
piR-hsa-1288731	Diseases of programmed cell death	0.509768763
piR-hsa-1288731	Meiosis	0.509768763
piR-hsa-1288731	RNA Polymerase I Promoter Opening	0.509768763
piR-hsa-1288731	Pre-NOTCH Transcription and Translation	0.509768763
piR-hsa-1288731	Maternal to zygotic transition (MZT)	0.509768763
piR-hsa-1288731	Chromatin organization	0.509768763
piR-hsa-1288731	HCMV Infection	0.509768763
piR-hsa-1288731	DNA Replication	0.509768763
piR-hsa-1288731	Epigenetic regulation of gene expression	0.509768763
piR-hsa-1288731	Signaling by WNT	0.509768763
piR-hsa-1288731	Meiotic recombination	0.509768763
piR-hsa-1288731	Defective pyroptosis	0.509768763
piR-hsa-1288731	HATs acetylate histones	0.509768763
piR-hsa-1288731	Amyloid fiber formation	0.509768763
piR-hsa-1288731	Gene Silencing by RNA	0.509768763
piR-hsa-1288731	Transcriptional regulation by small RNAs	0.509768763
piR-hsa-1288731	Activated PKN1 stimulates transcription of AR (androgen receptor) regulated genes KLK2 and KLK3	0.509768763
piR-hsa-1288731	Transcriptional regulation of granulopoiesis	0.509768763
piR-hsa-1288731	PRC2 methylates histones and DNA	0.509768763
piR-hsa-1288731	RNA Polymerase I Promoter Escape	0.509768763
piR-hsa-1288731	RUNX1 regulates genes involved in megakaryocyte differentiation and platelet function	0.509768763
piR-hsa-1288731	DNA Replication Pre-Initiation	0.509768763
piR-hsa-1288731	Activation of HOX genes during differentiation	0.509768763

piR-hsa-1288731	Assembly of the pre-replicative complex	0.509768763
piR-hsa-1288731	Oxidative Stress Induced Senescence	0.509768763
piR-hsa-1288731	HCMV Late Events	0.509768763
piR-hsa-1288731	NoRC negatively regulates rRNA expression	0.509768763
piR-hsa-1288731	SIRT1 negatively regulates rRNA expression	0.509768763
piR-hsa-1288731	Signaling by Rho GTPases	0.509768763
piR-hsa-1288731	Mitotic Prophase	0.509768763
piR-hsa-1288731	HDACs deacetylate histones	0.509768763
piR-hsa-1288731	ERCC6 (CSB) and EHMT2 (G9a) positively regulate rRNA expression	0.509768763
piR-hsa-1288731	Activation of anterior HOX genes in hindbrain development during early embryogenesis	0.509768763
piR-hsa-1288731	RUNX1 regulates transcription of genes involved in differentiation of HSCs	0.509768763
piR-hsa-1288731	Negative epigenetic regulation of rRNA expression	0.509768763
piR-hsa-1288731	RHO GTPases activate PKNs	0.509768763
piR-hsa-1288731	B-WICH complex positively regulates rRNA expression	0.509768763
piR-hsa-1288731	Estrogen-dependent gene expression	0.509768763
piR-hsa-1288731	Cellular Senescence	0.509768763
piR-hsa-1288731	Formation of the beta-catenin:TCF transactivating complex	0.509768763
piR-hsa-1288731	ESR-mediated signaling	0.509768763
piR-hsa-1288731	RMTs methylate histone arginines	0.509768763
piR-hsa-1288731	Condensation of Prophase Chromosomes	0.509768763
piR-hsa-1288731	Amyloid fiber formation	0.520116339
piR-hsa-1288731	Transcriptional regulation of granulopoiesis	0.520116339
piR-hsa-1288731	HCMV Infection	0.520116339
piR-hsa-1288731	Signaling by WNT	0.520116339
piR-hsa-1288731	Signaling by NOTCH	0.520116339
piR-hsa-1288731	DNA Replication	0.520116339
piR-hsa-1288731	RNA Polymerase I Transcription	0.520116339
piR-hsa-1288731	Maternal to zygotic transition (MZT)	0.520116339
piR-hsa-1288731	RNA Polymerase I Promoter Clearance	0.520116339
piR-hsa-1288731	NoRC negatively regulates rRNA expression	0.520116339
piR-hsa-1288731	Assembly of the ORC complex at the origin of replication	0.520116339
piR-hsa-1288731	Activation of HOX genes during differentiation	0.520116339
piR-hsa-1288731	Formation of the beta-catenin:TCF transactivating complex	0.520116339
piR-hsa-1288731	ESR-mediated signaling	0.520116339
piR-hsa-1288731	Chromatin modifying enzymes	0.520116339
piR-hsa-1288731	PRC2 methylates histones and DNA	0.520116339
piR-hsa-1288731	Pre-NOTCH Transcription and Translation	0.520116339

piR-hsa-1288731	Gene Silencing by RNA	0.520116339
piR-hsa-1288731	Estrogen-dependent gene expression	0.520116339
piR-hsa-1288731	Epigenetic regulation of gene expression	0.520116339
piR-hsa-1288731	Chromatin organization	0.520116339
piR-hsa-1288731	Cellular Senescence	0.520116339
piR-hsa-1288731	Meiosis	0.520116339
piR-hsa-1288731	RUNX1 regulates genes involved in megakaryocyte differentiation and platelet function	0.520116339
piR-hsa-1288731	RNA Polymerase I Promoter Opening	0.520116339
piR-hsa-1288731	Positive epigenetic regulation of rRNA expression	0.520116339
piR-hsa-1288731	RMTs methylate histone arginines	0.520116339
piR-hsa-1288731	Negative epigenetic regulation of rRNA expression	0.520116339
piR-hsa-1288731	Mitotic Prophase	0.520116339
piR-hsa-1288731	ERCC6 (CSB) and EHMT2 (G9a) positively regulate rRNA expression	0.520116339
piR-hsa-1288731	SIRT1 negatively regulates rRNA expression	0.520116339
piR-hsa-1288731	Transcriptional regulation by small RNAs	0.520116339
piR-hsa-1288731	Oxidative Stress Induced Senescence	0.520116339
piR-hsa-1288731	HDACs deacetylate histones	0.520116339
piR-hsa-1288731	Activation of anterior HOX genes in hindbrain development during early embryogenesis	0.520116339
piR-hsa-1288731	B-WICH complex positively regulates rRNA expression	0.520116339
piR-hsa-1288731	HCMV Early Events	0.520116339
piR-hsa-1288731	RNA Polymerase I Promoter Escape	0.520116339
piR-hsa-1288731	RUNX1 regulates transcription of genes involved in differentiation of HSCs	0.520116339
piR-hsa-1288731	Meiotic recombination	0.520116339
piR-hsa-1288731	Diseases of programmed cell death	0.520116339
piR-hsa-1288731	DNA Replication Pre-Initiation	0.520116339
piR-hsa-1288731	RHO GTPases activate PKNs	0.520116339
piR-hsa-1288731	RHO GTPase Effectors	0.520116339
piR-hsa-1288731	Senescence-Associated Secretory Phenotype (SASP)	0.520116339
piR-hsa-1288731	Signaling by Rho GTPases, Miro GTPases and RHOBTB3	0.520116339
piR-hsa-1288731	Activated PKN1 stimulates transcription of AR (androgen receptor) regulated genes KLK2 and KLK3	0.520116339
piR-hsa-1288731	Condensation of Prophase Chromosomes	0.520116339
piR-hsa-1288731	DNA methylation	0.520116339
piR-hsa-1288731	Defective pyroptosis	0.520116339
piR-hsa-1288731	TCF dependent signaling in response to WNT	0.520116339
piR-hsa-1288731	Signaling by Rho GTPases	0.520116339
piR-hsa-1288731	Reproduction	0.520116339
piR-hsa-1288731	Signaling by Nuclear Receptors	0.520116339

piR-hsa-1288731	HCMV Late Events	0.520116339
piR-hsa-1288731	Pre-NOTCH Expression and Processing	0.520116339
piR-hsa-1288731	HATs acetylate histones	0.520116339
piR-hsa-1288731	Chromatin modifications during the maternal to zygotic transition (MZT)	0.520116339
piR-hsa-1288731	Assembly of the pre-replicative complex	0.520116339
piR-hsa-1288731	HATs acetylate histones	0.510210549
piR-hsa-1288731	Pre-NOTCH Expression and Processing	0.510210549
piR-hsa-1288731	HCMV Late Events	0.510210549
piR-hsa-1288731	Signaling by NOTCH	0.510210549
piR-hsa-1288731	Senescence-Associated Secretory Phenotype (SASP)	0.510210549
piR-hsa-1288731	Chromatin modifying enzymes	0.510210549
piR-hsa-1288731	SIRT1 negatively regulates rRNA expression	0.510210549
piR-hsa-1288731	Activation of HOX genes during differentiation	0.510210549
piR-hsa-1288731	Positive epigenetic regulation of rRNA expression	0.510210549
piR-hsa-1288731	RNA Polymerase I Transcription	0.510210549
piR-hsa-1288731	RUNX1 regulates transcription of genes involved in differentiation of HSCs	0.510210549
piR-hsa-1288731	DNA methylation	0.510210549
piR-hsa-1288731	Defective pyroptosis	0.510210549
piR-hsa-1288731	RHO GTPases activate PKNs	0.510210549
piR-hsa-1288731	Estrogen-dependent gene expression	0.510210549
piR-hsa-1288731	Chromatin modifications during the maternal to zygotic transition (MZT)	0.510210549
piR-hsa-1288731	HDACs deacetylate histones	0.510210549
piR-hsa-1288731	Oxidative Stress Induced Senescence	0.510210549
piR-hsa-1288731	DNA Replication Pre-Initiation	0.510210549
piR-hsa-1288731	Maternal to zygotic transition (MZT)	0.510210549
piR-hsa-1288731	ERCC6 (CSB) and EHMT2 (G9a) positively regulate rRNA expression	0.510210549
piR-hsa-1288731	Signaling by Rho GTPases	0.510210549
piR-hsa-1288731	ESR-mediated signaling	0.510210549
piR-hsa-1288731	Signaling by Nuclear Receptors	0.510210549
piR-hsa-1288731	Condensation of Prophase Chromosomes	0.510210549
piR-hsa-1288731	RMTs methylate histone arginines	0.510210549
piR-hsa-1288731	TCF dependent signaling in response to WNT	0.510210549
piR-hsa-1288731	RNA Polymerase I Promoter Opening	0.510210549
piR-hsa-1288731	Epigenetic regulation of gene expression	0.510210549
piR-hsa-1288731	Transcriptional regulation by small RNAs	0.510210549
piR-hsa-1288731	Negative epigenetic regulation of rRNA expression	0.510210549
piR-hsa-1288731	RNA Polymerase I Promoter Clearance	0.510210549

piR-hsa-1288731	Amyloid fiber formation	0.510210549
piR-hsa-1288731	Meiosis	0.510210549
piR-hsa-1288731	RUNX1 regulates genes involved in megakaryocyte differentiation and platelet function	0.510210549
piR-hsa-1288731	Mitotic Prophase	0.510210549
piR-hsa-1288731	Activation of anterior HOX genes in hindbrain development during early embryogenesis	0.510210549
piR-hsa-1288731	Gene Silencing by RNA	0.510210549
piR-hsa-1288731	RHO GTPase Effectors	0.510210549
piR-hsa-1288731	Assembly of the pre-replicative complex	0.510210549
piR-hsa-1288731	Transcriptional regulation of granulopoiesis	0.510210549
piR-hsa-1288731	B-WICH complex positively regulates rRNA expression	0.510210549
piR-hsa-1288731	Reproduction	0.510210549
piR-hsa-1288731	RNA Polymerase I Promoter Escape	0.510210549
piR-hsa-1288731	Activated PKN1 stimulates transcription of AR (androgen receptor) regulated genes KLK2 and KLK3	0.510210549
piR-hsa-1288731	Chromatin organization	0.510210549
piR-hsa-1288731	NoRC negatively regulates rRNA expression	0.510210549
piR-hsa-1288731	Signaling by WNT	0.510210549
piR-hsa-1288731	HCMV Early Events	0.510210549
piR-hsa-1288731	HCMV Infection	0.510210549
piR-hsa-1288731	Diseases of programmed cell death	0.510210549
piR-hsa-1288731	Pre-NOTCH Transcription and Translation	0.510210549
piR-hsa-1288731	DNA Replication	0.510210549
piR-hsa-1288731	Cellular Senescence	0.510210549
piR-hsa-1288731	Signaling by Rho GTPases, Miro GTPases and RHOBTB3	0.510210549
piR-hsa-1288731	Formation of the beta-catenin:TCF transactivating complex	0.510210549
piR-hsa-1288731	PRC2 methylates histones and DNA	0.510210549
piR-hsa-1288731	Meiotic recombination	0.510210549
piR-hsa-1288731	Assembly of the ORC complex at the origin of replication	0.510210549
piR-hsa-1288731	Deposition of new CENPA-containing nucleosomes at the centromere	0.506206485
piR-hsa-1288731	Diseases of programmed cell death	0.506206485
piR-hsa-1288731	Transcriptional regulation by small RNAs	0.506206485
piR-hsa-1288731	HCMV Early Events	0.506206485
piR-hsa-1288731	Depurination	0.506206485
piR-hsa-1288731	Signaling by Rho GTPases, Miro GTPases and RHOBTB3	0.506206485
piR-hsa-1288731	Chromosome Maintenance	0.506206485
piR-hsa-1288731	Transcriptional regulation of granulopoiesis	0.506206485
piR-hsa-1288731	TCF dependent signaling in response to WNT	0.506206485
piR-hsa-1288731	Signaling by WNT	0.506206485

piR-hsa-1288731	Cleavage of the damaged purine	0.506206485
piR-hsa-1288731	Recruitment and ATM-mediated phosphorylation of repair and signaling proteins at DNA double strand breaks	0.506206485
piR-hsa-1288731	Packaging Of Telomere Ends	0.506206485
piR-hsa-1288731	Assembly of the pre-replicative complex	0.506206485
piR-hsa-1288731	Chromatin modifying enzymes	0.506206485
piR-hsa-1288731	Chromatin modifications during the maternal to zygotic transition (MZT)	0.506206485
piR-hsa-1288731	Replacement of protamines by nucleosomes in the male pronucleus	0.506206485
piR-hsa-1288731	G2/M DNA damage checkpoint	0.506206485
piR-hsa-1288731	Base Excision Repair	0.506206485
piR-hsa-1288731	Mitotic Prophase	0.506206485
piR-hsa-1288731	RHO GTPase Effectors	0.506206485
piR-hsa-1288731	Activation of anterior HOX genes in hindbrain development during early embryogenesis	0.506206485
piR-hsa-1288731	Cell Cycle Checkpoints	0.506206485
piR-hsa-1288731	Reproduction	0.506206485
piR-hsa-1288731	Depyrimidination	0.506206485
piR-hsa-1288731	Meiotic recombination	0.506206485
piR-hsa-1288731	Positive epigenetic regulation of rRNA expression	0.506206485
piR-hsa-1288731	Oxidative Stress Induced Senescence	0.506206485
piR-hsa-1288731	Pre-NOTCH Transcription and Translation	0.506206485
piR-hsa-1288731	ERCC6 (CSB) and EHMT2 (G9a) positively regulate rRNA expression	0.506206485
piR-hsa-1288731	Telomere Maintenance	0.506206485
piR-hsa-1288731	Nucleosome assembly	0.506206485
piR-hsa-1288731	Maternal to zygotic transition (MZT)	0.506206485
piR-hsa-1288731	RNA Polymerase I Transcription	0.506206485
piR-hsa-1288731	Signaling by NOTCH	0.506206485
piR-hsa-1288731	Signaling by Nuclear Receptors	0.506206485
piR-hsa-1288731	Meiotic synapsis	0.506206485
piR-hsa-1288731	Defective pyroptosis	0.506206485
piR-hsa-1288731	Senescence-Associated Secretory Phenotype (SASP)	0.506206485
piR-hsa-1288731	Inhibition of DNA recombination at telomere	0.506206485
piR-hsa-1288731	SIRT1 negatively regulates rRNA expression	0.506206485
piR-hsa-1288731	ESR-mediated signaling	0.506206485
piR-hsa-1288731	DNA methylation	0.506206485
piR-hsa-1288731	HCMV Infection	0.506206485
piR-hsa-1288731	PRC2 methylates histones and DNA	0.506206485
piR-hsa-1288731	RUNX1 regulates genes involved in megakaryocyte differentiation and platelet function	0.506206485
piR-hsa-1288731	RNA Polymerase I Promoter Clearance	0.506206485

piR-hsa-1288731	Epigenetic regulation of gene expression	0.506206485
piR-hsa-1288731	Processing of DNA double-strand break ends	0.506206485
piR-hsa-1288731	HCMV Late Events	0.506206485
piR-hsa-1288731	Amyloid fiber formation	0.506206485
piR-hsa-1288731	HATs acetylate histones	0.506206485
piR-hsa-1288731	Recognition and association of DNA glycosylase with site containing an affected pyrimidine	0.506206485
piR-hsa-1288731	DNA Double Strand Break Response	0.506206485
piR-hsa-1288731	Base-Excision Repair, AP Site Formation	0.506206485
piR-hsa-1288731	RMTs methylate histone arginines	0.506206485
piR-hsa-1288731	NoRC negatively regulates rRNA expression	0.506206485
piR-hsa-1288731	Cleavage of the damaged pyrimidine	0.506206485
piR-hsa-1288731	DNA Replication	0.506206485
piR-hsa-1288731	RNA Polymerase I Promoter Opening	0.506206485
piR-hsa-1288731	DNA Replication Pre-Initiation	0.506206485
piR-hsa-1288731	RHO GTPases activate PKNs	0.506206485
piR-hsa-1288731	Pre-NOTCH Expression and Processing	0.506206485
piR-hsa-1288731	RUNX1 regulates transcription of genes involved in differentiation of HSCs	0.506206485
piR-hsa-1288731	G2/M Checkpoints	0.506206485
piR-hsa-1288731	Activated PKN1 stimulates transcription of AR (androgen receptor) regulated genes KLK2 and KLK3	0.506206485
piR-hsa-1288731	RNA Polymerase I Promoter Escape	0.506206485
piR-hsa-1288731	DNA Damage/Telomere Stress Induced Senescence	0.506206485
piR-hsa-1288731	Gene Silencing by RNA	0.506206485
piR-hsa-1288731	Chromatin organization	0.506206485
piR-hsa-1288731	Estrogen-dependent gene expression	0.506206485
piR-hsa-1288731	Activation of HOX genes during differentiation	0.506206485
piR-hsa-1288731	Assembly of the ORC complex at the origin of replication	0.506206485
piR-hsa-1288731	Nonhomologous End-Joining (NHEJ)	0.506206485
piR-hsa-1288731	Condensation of Prophase Chromosomes	0.506206485
piR-hsa-1288731	Signaling by Rho GTPases	0.506206485
piR-hsa-1288731	B-WICH complex positively regulates rRNA expression	0.506206485
piR-hsa-1288731	Recognition and association of DNA glycosylase with site containing an affected purine	0.506206485
piR-hsa-1288731	HDACs deacetylate histones	0.506206485
piR-hsa-1288731	Negative epigenetic regulation of rRNA expression	0.506206485
piR-hsa-1288731	Cellular Senescence	0.506206485
piR-hsa-1288731	Formation of the beta-catenin:TCF transactivating complex	0.506206485
piR-hsa-1288731	Meiosis	0.506206485
piR-hsa-1288731	RNA Polymerase I Promoter Clearance	0.473836481

piR-hsa-1288731	Inhibition of DNA recombination at telomere	0.473836481
piR-hsa-1288731	Packaging Of Telomere Ends	0.473836481
piR-hsa-1288731	Depurination	0.473836481
piR-hsa-1288731	Activated PKN1 stimulates transcription of AR (androgen receptor) regulated genes KLK2 and KLK3	0.473836481
piR-hsa-1288731	Formation of the beta-catenin:TCF transactivating complex	0.473836481
piR-hsa-1288731	HCMV Infection	0.473836481
piR-hsa-1288731	ERCC6 (CSB) and EHMT2 (G9a) positively regulate rRNA expression	0.473836481
piR-hsa-1288731	Signaling by Rho GTPases, Miro GTPases and RHOBTB3	0.473836481
piR-hsa-1288731	B-WICH complex positively regulates rRNA expression	0.473836481
piR-hsa-1288731	RHO GTPase Effectors	0.473836481
piR-hsa-1288731	SIRT1 negatively regulates rRNA expression	0.473836481
piR-hsa-1288731	G2/M DNA damage checkpoint	0.473836481
piR-hsa-1288731	Signaling by WNT	0.473836481
piR-hsa-1288731	Deposition of new CENPA-containing nucleosomes at the centromere	0.473836481
piR-hsa-1288731	Cellular Senescence	0.473836481
piR-hsa-1288731	RNA Polymerase I Promoter Opening	0.473836481
piR-hsa-1288731	Mitotic Prophase	0.473836481
piR-hsa-1288731	ESR-mediated signaling	0.473836481
piR-hsa-1288731	Cleavage of the damaged purine	0.473836481
piR-hsa-1288731	Amyloid fiber formation	0.473836481
piR-hsa-1288731	Chromatin organization	0.473836481
piR-hsa-1288731	Reproduction	0.473836481
piR-hsa-1288731	Epigenetic regulation of gene expression	0.473836481
piR-hsa-1288731	Recognition and association of DNA glycosylase with site containing an affected pyrimidine	0.473836481
piR-hsa-1288731	Signaling by NOTCH	0.473836481
piR-hsa-1288731	Signaling by Rho GTPases	0.473836481
piR-hsa-1288731	RMTs methylate histone arginines	0.473836481
piR-hsa-1288731	Negative epigenetic regulation of rRNA expression	0.473836481
piR-hsa-1288731	DNA Damage/Telomere Stress Induced Senescence	0.473836481
piR-hsa-1288731	Condensation of Prophase Chromosomes	0.473836481
piR-hsa-1288731	Assembly of the pre-replicative complex	0.473836481
piR-hsa-1288731	Estrogen-dependent gene expression	0.473836481
piR-hsa-1288731	Activation of anterior HOX genes in hindbrain development during early embryogenesis	0.473836481
piR-hsa-1288731	HCMV Late Events	0.473836481
piR-hsa-1288731	HDACs deacetylate histones	0.473836481
piR-hsa-1288731	Chromosome Maintenance	0.473836481
piR-hsa-1288731	Meiotic synapsis	0.473836481

piR-hsa-1288731	RNA Polymerase I Transcription	0.473836481
piR-hsa-1288731	Diseases of programmed cell death	0.473836481
piR-hsa-1288731	Oxidative Stress Induced Senescence	0.473836481
piR-hsa-1288731	G2/M Checkpoints	0.473836481
piR-hsa-1288731	Senescence-Associated Secretory Phenotype (SASP)	0.473836481
piR-hsa-1288731	Chromatin modifications during the maternal to zygotic transition (MZT)	0.473836481
piR-hsa-1288731	DNA Replication Pre-Initiation	0.473836481
piR-hsa-1288731	Pre-NOTCH Transcription and Translation	0.473836481
piR-hsa-1288731	TCF dependent signaling in response to WNT	0.473836481
piR-hsa-1288731	RUNX1 regulates genes involved in megakaryocyte differentiation and platelet function	0.473836481
piR-hsa-1288731	Nucleosome assembly	0.473836481
piR-hsa-1288731	DNA Replication	0.473836481
piR-hsa-1288731	Maternal to zygotic transition (MZT)	0.473836481
piR-hsa-1288731	Activation of HOX genes during differentiation	0.473836481
piR-hsa-1288731	Defective pyroptosis	0.473836481
piR-hsa-1288731	Gene Silencing by RNA	0.473836481
piR-hsa-1288731	Meiotic recombination	0.473836481
piR-hsa-1288731	Telomere Maintenance	0.473836481
piR-hsa-1288731	PRC2 methylates histones and DNA	0.473836481
piR-hsa-1288731	Recognition and association of DNA glycosylase with site containing an affected purine	0.473836481
piR-hsa-1288731	RUNX1 regulates transcription of genes involved in differentiation of HSCs	0.473836481
piR-hsa-1288731	RHO GTPases activate PKNs	0.473836481
piR-hsa-1288731	HCMV Early Events	0.473836481
piR-hsa-1288731	Signaling by Nuclear Receptors	0.473836481
piR-hsa-1288731	Base Excision Repair	0.473836481
piR-hsa-1288731	Depyrimidination	0.473836481
piR-hsa-1288731	Pre-NOTCH Expression and Processing	0.473836481
piR-hsa-1288731	Transcriptional regulation of granulopoiesis	0.473836481
piR-hsa-1288731	Assembly of the ORC complex at the origin of replication	0.473836481
piR-hsa-1288731	Recruitment and ATM-mediated phosphorylation of repair and signaling proteins at DNA double strand breaks	0.473836481
piR-hsa-1288731	Positive epigenetic regulation of rRNA expression	0.473836481
piR-hsa-1288731	RNA Polymerase I Promoter Escape	0.473836481
piR-hsa-1288731	DNA Double Strand Break Response	0.473836481
piR-hsa-1288731	Meiosis	0.473836481
piR-hsa-1288731	DNA methylation	0.473836481
piR-hsa-1288731	Nonhomologous End-Joining (NHEJ)	0.473836481
piR-hsa-1288731	HATs acetylate histones	0.473836481

piR-hsa-1288731	NoRC negatively regulates rRNA expression	0.473836481
piR-hsa-1288731	Chromatin modifying enzymes	0.473836481
piR-hsa-1288731	Processing of DNA double-strand break ends	0.473836481
piR-hsa-1288731	Replacement of protamines by nucleosomes in the male pronucleus	0.473836481
piR-hsa-1288731	Cleavage of the damaged pyrimidine	0.473836481
piR-hsa-1288731	Cell Cycle Checkpoints	0.473836481
piR-hsa-1288731	Base-Excision Repair, AP Site Formation	0.473836481
piR-hsa-1288731	Transcriptional regulation by small RNAs	0.473836481
piR-hsa-1288731	Pre-NOTCH Expression and Processing	0.522485568
piR-hsa-1288731	RNA Polymerase I Transcription	0.522485568
piR-hsa-1288731	Maternal to zygotic transition (MZT)	0.522485568
piR-hsa-1288731	Replacement of protamines by nucleosomes in the male pronucleus	0.522485568
piR-hsa-1288731	Cleavage of the damaged purine	0.522485568
piR-hsa-1288731	Signaling by NOTCH	0.522485568
piR-hsa-1288731	Chromatin modifying enzymes	0.522485568
piR-hsa-1288731	RNA Polymerase I Promoter Clearance	0.522485568
piR-hsa-1288731	RUNX1 regulates genes involved in megakaryocyte differentiation and platelet function	0.522485568
piR-hsa-1288731	Nucleosome assembly	0.522485568
piR-hsa-1288731	Activation of anterior HOX genes in hindbrain development during early embryogenesis	0.522485568
piR-hsa-1288731	Deposition of new CENPA-containing nucleosomes at the centromere	0.522485568
piR-hsa-1288731	Amyloid fiber formation	0.522485568
piR-hsa-1288731	Activation of HOX genes during differentiation	0.522485568
piR-hsa-1288731	PRC2 methylates histones and DNA	0.522485568
piR-hsa-1288731	RMTs methylate histone arginines	0.522485568
piR-hsa-1288731	Chromatin modifications during the maternal to zygotic transition (MZT)	0.522485568
piR-hsa-1288731	Transcriptional regulation by small RNAs	0.522485568
piR-hsa-1288731	Telomere Maintenance	0.522485568
piR-hsa-1288731	G2/M DNA damage checkpoint	0.522485568
piR-hsa-1288731	Packaging Of Telomere Ends	0.522485568
piR-hsa-1288731	Transcriptional regulation of granulopoiesis	0.522485568
piR-hsa-1288731	NoRC negatively regulates rRNA expression	0.522485568
piR-hsa-1288731	Gene Silencing by RNA	0.522485568
piR-hsa-1288731	DNA Replication	0.522485568
piR-hsa-1288731	Signaling by WNT	0.522485568
piR-hsa-1288731	Recruitment and ATM-mediated phosphorylation of repair and signaling proteins at DNA double strand breaks	0.522485568
piR-hsa-1288731	HCMV Infection	0.522485568
piR-hsa-1288731	DNA Double Strand Break Response	0.522485568

piR-hsa-1288731	Meiotic synapsis	0.522485568
piR-hsa-1288731	Signaling by Rho GTPases, Miro GTPases and RHOBTB3	0.522485568
piR-hsa-1288731	Inhibition of DNA recombination at telomere	0.522485568
piR-hsa-1288731	Meiosis	0.522485568
piR-hsa-1288731	Nonhomologous End-Joining (NHEJ)	0.522485568
piR-hsa-1288731	ERCC6 (CSB) and EHMT2 (G9a) positively regulate rRNA expression	0.522485568
piR-hsa-1288731	Chromatin organization	0.522485568
piR-hsa-1288731	Assembly of the ORC complex at the origin of replication	0.522485568
piR-hsa-1288731	Reproduction	0.522485568
piR-hsa-1288731	DNA Damage/Telomere Stress Induced Senescence	0.522485568
piR-hsa-1288731	Processing of DNA double-strand break ends	0.522485568
piR-hsa-1288731	HCMV Early Events	0.522485568
piR-hsa-1288731	DNA methylation	0.522485568
piR-hsa-1288731	TCF dependent signaling in response to WNT	0.522485568
piR-hsa-1288731	Assembly of the pre-replicative complex	0.522485568
piR-hsa-1288731	Depyrimidination	0.522485568
piR-hsa-1288731	SIRT1 negatively regulates rRNA expression	0.522485568
piR-hsa-1288731	Recognition and association of DNA glycosylase with site containing an affected pyrimidine	0.522485568
piR-hsa-1288731	RNA Polymerase I Promoter Escape	0.522485568
piR-hsa-1288731	Pre-NOTCH Transcription and Translation	0.522485568
piR-hsa-1288731	B-WICH complex positively regulates rRNA expression	0.522485568
piR-hsa-1288731	HDACs deacetylate histones	0.522485568
piR-hsa-1288731	Cleavage of the damaged pyrimidine	0.522485568
piR-hsa-1288731	Epigenetic regulation of gene expression	0.522485568
piR-hsa-1288731	Diseases of programmed cell death	0.522485568
piR-hsa-1288731	Chromosome Maintenance	0.522485568
piR-hsa-1288731	Depurination	0.522485568
piR-hsa-1288731	Signaling by Nuclear Receptors	0.522485568
piR-hsa-1288731	Senescence-Associated Secretory Phenotype (SASP)	0.522485568
piR-hsa-1288731	Base Excision Repair	0.522485568
piR-hsa-1288731	RHO GTPase Effectors	0.522485568
piR-hsa-1288731	RNA Polymerase I Promoter Opening	0.522485568
piR-hsa-1288731	Formation of the beta-catenin:TCF transactivating complex	0.522485568
piR-hsa-1288731	Activated PKN1 stimulates transcription of AR (androgen receptor) regulated genes KLK2 and KLK3	0.522485568
piR-hsa-1288731	Cellular Senescence	0.522485568
piR-hsa-1288731	Cell Cycle Checkpoints	0.522485568
piR-hsa-1288731	RUNX1 regulates transcription of genes involved in differentiation of HSCs	0.522485568

piR-hsa-1288731	Estrogen-dependent gene expression	0.522485568
piR-hsa-1288731	DNA Replication Pre-Initiation	0.522485568
piR-hsa-1288731	Defective pyroptosis	0.522485568
piR-hsa-1288731	Positive epigenetic regulation of rRNA expression	0.522485568
piR-hsa-1288731	Meiotic recombination	0.522485568
piR-hsa-1288731	ESR-mediated signaling	0.522485568
piR-hsa-1288731	Condensation of Prophase Chromosomes	0.522485568
piR-hsa-1288731	Base-Excision Repair, AP Site Formation	0.522485568
piR-hsa-1288731	Negative epigenetic regulation of rRNA expression	0.522485568
piR-hsa-1288731	HCMV Late Events	0.522485568
piR-hsa-1288731	Mitotic Prophase	0.522485568
piR-hsa-1288731	RHO GTPases activate PKNs	0.522485568
piR-hsa-1288731	Recognition and association of DNA glycosylase with site containing an affected purine	0.522485568
piR-hsa-1288731	G2/M Checkpoints	0.522485568
piR-hsa-1288731	HATs acetylate histones	0.522485568
piR-hsa-1288731	Oxidative Stress Induced Senescence	0.522485568
piR-hsa-1288731	Signaling by Rho GTPases	0.522485568
piR-hsa-1288731	Assembly of the pre-replicative complex	0.522593225
piR-hsa-1288731	Chromatin modifications during the maternal to zygotic transition (MZT)	0.522593225
piR-hsa-1288731	Base Excision Repair	0.522593225
piR-hsa-1288731	Positive epigenetic regulation of rRNA expression	0.522593225
piR-hsa-1288731	HCMV Late Events	0.522593225
piR-hsa-1288731	HATs acetylate histones	0.522593225
piR-hsa-1288731	Diseases of programmed cell death	0.522593225
piR-hsa-1288731	Oxidative Stress Induced Senescence	0.522593225
piR-hsa-1288731	RNA Polymerase I Transcription	0.522593225
piR-hsa-1288731	Senescence-Associated Secretory Phenotype (SASP)	0.522593225
piR-hsa-1288731	Amyloid fiber formation	0.522593225
piR-hsa-1288731	Depyrimidination	0.522593225
piR-hsa-1288731	SIRT1 negatively regulates rRNA expression	0.522593225
piR-hsa-1288731	Telomere Maintenance	0.522593225
piR-hsa-1288731	RNA Polymerase I Promoter Opening	0.522593225
piR-hsa-1288731	Signaling by Nuclear Receptors	0.522593225
piR-hsa-1288731	Meiotic synapsis	0.522593225
piR-hsa-1288731	DNA Replication Pre-Initiation	0.522593225
piR-hsa-1288731	Pre-NOTCH Expression and Processing	0.522593225
piR-hsa-1288731	Recognition and association of DNA glycosylase with site containing an affected purine	0.522593225

piR-hsa-1288731	Chromosome Maintenance	0.522593225
piR-hsa-1288731	Transcriptional regulation by small RNAs	0.522593225
piR-hsa-1288731	RHO GTPases activate PKNs	0.522593225
piR-hsa-1288731	Activation of HOX genes during differentiation	0.522593225
piR-hsa-1288731	Chromatin organization	0.522593225
piR-hsa-1288731	Defective pyroptosis	0.522593225
piR-hsa-1288731	Maternal to zygotic transition (MZT)	0.522593225
piR-hsa-1288731	Recognition and association of DNA glycosylase with site containing an affected pyrimidine	0.522593225
piR-hsa-1288731	Cell Cycle Checkpoints	0.522593225
piR-hsa-1288731	Signaling by NOTCH	0.522593225
piR-hsa-1288731	B-WICH complex positively regulates rRNA expression	0.522593225
piR-hsa-1288731	RNA Polymerase I Promoter Clearance	0.522593225
piR-hsa-1288731	RUNX1 regulates transcription of genes involved in differentiation of HSCs	0.522593225
piR-hsa-1288731	Activation of anterior HOX genes in hindbrain development during early embryogenesis	0.522593225
piR-hsa-1288731	ESR-mediated signaling	0.522593225
piR-hsa-1288731	Cleavage of the damaged pyrimidine	0.522593225
piR-hsa-1288731	HCMV Early Events	0.522593225
piR-hsa-1288731	RHO GTPase Effectors	0.522593225
piR-hsa-1288731	Estrogen-dependent gene expression	0.522593225
piR-hsa-1288731	RUNX1 regulates genes involved in megakaryocyte differentiation and platelet function	0.522593225
piR-hsa-1288731	Signaling by WNT	0.522593225
piR-hsa-1288731	RMTs methylate histone arginines	0.522593225
piR-hsa-1288731	Nucleosome assembly	0.522593225
piR-hsa-1288731	Replacement of protamines by nucleosomes in the male pronucleus	0.522593225
piR-hsa-1288731	Gene Silencing by RNA	0.522593225
piR-hsa-1288731	Chromatin modifying enzymes	0.522593225
piR-hsa-1288731	Inhibition of DNA recombination at telomere	0.522593225
piR-hsa-1288731	PRC2 methylates histones and DNA	0.522593225
piR-hsa-1288731	Depurination	0.522593225
piR-hsa-1288731	Deposition of new CENPA-containing nucleosomes at the centromere	0.522593225
piR-hsa-1288731	Reproduction	0.522593225
piR-hsa-1288731	Processing of DNA double-strand break ends	0.522593225
piR-hsa-1288731	Epigenetic regulation of gene expression	0.522593225
piR-hsa-1288731	Pre-NOTCH Transcription and Translation	0.522593225
piR-hsa-1288731	RNA Polymerase I Promoter Escape	0.522593225
piR-hsa-1288731	DNA Double Strand Break Response	0.522593225
piR-hsa-1288731	Packaging Of Telomere Ends	0.522593225

piR-hsa-1288731	DNA Damage/Telomere Stress Induced Senescence	0.522593225
piR-hsa-1288731	Assembly of the ORC complex at the origin of replication	0.522593225
piR-hsa-1288731	Condensation of Prophase Chromosomes	0.522593225
piR-hsa-1288731	Recruitment and ATM-mediated phosphorylation of repair and signaling proteins at DNA double strand breaks	0.522593225
piR-hsa-1288731	Activated PKN1 stimulates transcription of AR (androgen receptor) regulated genes KLK2 and KLK3	0.522593225
piR-hsa-1288731	HCMV Infection	0.522593225
piR-hsa-1288731	Nonhomologous End-Joining (NHEJ)	0.522593225
piR-hsa-1288731	Transcriptional regulation of granulopoiesis	0.522593225
piR-hsa-1288731	HDACs deacetylate histones	0.522593225
piR-hsa-1288731	TCF dependent signaling in response to WNT	0.522593225
piR-hsa-1288731	Meiosis	0.522593225
piR-hsa-1288731	Formation of the beta-catenin:TCF transactivating complex	0.522593225
piR-hsa-1288731	Signaling by Rho GTPases, Miro GTPases and RHOBTB3	0.522593225
piR-hsa-1288731	DNA methylation	0.522593225
piR-hsa-1288731	Cellular Senescence	0.522593225
piR-hsa-1288731	Mitotic Prophase	0.522593225
piR-hsa-1288731	Negative epigenetic regulation of rRNA expression	0.522593225
piR-hsa-1288731	NoRC negatively regulates rRNA expression	0.522593225
piR-hsa-1288731	DNA Replication	0.522593225
piR-hsa-1288731	G2/M DNA damage checkpoint	0.522593225
piR-hsa-1288731	Signaling by Rho GTPases	0.522593225
piR-hsa-1288731	Base-Excision Repair, AP Site Formation	0.522593225
piR-hsa-1288731	G2/M Checkpoints	0.522593225
piR-hsa-1288731	Meiotic recombination	0.522593225
piR-hsa-1288731	ERCC6 (CSB) and EHMT2 (G9a) positively regulate rRNA expression	0.522593225
piR-hsa-1288731	Cleavage of the damaged purine	0.522593225
piR-hsa-1288731	Senescence-Associated Secretory Phenotype (SASP)	0.517425812
piR-hsa-1288731	Depyrimidination	0.517425812
piR-hsa-1288731	TCF dependent signaling in response to WNT	0.517425812
piR-hsa-1288731	Base Excision Repair	0.517425812
piR-hsa-1288731	Transcriptional regulation by small RNAs	0.517425812
piR-hsa-1288731	Oxidative Stress Induced Senescence	0.517425812
piR-hsa-1288731	Assembly of the pre-replicative complex	0.517425812
piR-hsa-1288731	Diseases of programmed cell death	0.517425812
piR-hsa-1288731	Chromatin modifications during the maternal to zygotic transition (MZT)	0.517425812
piR-hsa-1288731	Cleavage of the damaged purine	0.517425812
piR-hsa-1288731	HATs acetylate histones	0.517425812

piR-hsa-1288731	Packaging Of Telomere Ends	0.517425812
piR-hsa-1288731	RNA Polymerase I Promoter Clearance	0.517425812
piR-hsa-1288731	Signaling by WNT	0.517425812
piR-hsa-1288731	RHO GTPase Effectors	0.517425812
piR-hsa-1288731	G2/M DNA damage checkpoint	0.517425812
piR-hsa-1288731	Positive epigenetic regulation of rRNA expression	0.517425812
piR-hsa-1288731	Amyloid fiber formation	0.517425812
piR-hsa-1288731	Meiotic synapsis	0.517425812
piR-hsa-1288731	Activation of anterior HOX genes in hindbrain development during early embryogenesis	0.517425812
piR-hsa-1288731	Signaling by Nuclear Receptors	0.517425812
piR-hsa-1288731	Depurination	0.517425812
piR-hsa-1288731	RNA Polymerase I Transcription	0.517425812
piR-hsa-1288731	Chromatin modifying enzymes	0.517425812
piR-hsa-1288731	Recognition and association of DNA glycosylase with site containing an affected pyrimidine	0.517425812
piR-hsa-1288731	Signaling by NOTCH	0.517425812
piR-hsa-1288731	Nucleosome assembly	0.517425812
piR-hsa-1288731	Cell Cycle Checkpoints	0.517425812
piR-hsa-1288731	Transcriptional regulation of granulopoiesis	0.517425812
piR-hsa-1288731	Chromosome Maintenance	0.517425812
piR-hsa-1288731	Defective pyroptosis	0.517425812
piR-hsa-1288731	Pre-NOTCH Expression and Processing	0.517425812
piR-hsa-1288731	Epigenetic regulation of gene expression	0.517425812
piR-hsa-1288731	RNA Polymerase I Promoter Opening	0.517425812
piR-hsa-1288731	SIRT1 negatively regulates rRNA expression	0.517425812
piR-hsa-1288731	HCMV Early Events	0.517425812
piR-hsa-1288731	Nonhomologous End-Joining (NHEJ)	0.517425812
piR-hsa-1288731	Mitotic Prophase	0.517425812
piR-hsa-1288731	Reproduction	0.517425812
piR-hsa-1288731	DNA Damage/Telomere Stress Induced Senescence	0.517425812
piR-hsa-1288731	RUNX1 regulates transcription of genes involved in differentiation of HSCs	0.517425812
piR-hsa-1288731	Estrogen-dependent gene expression	0.517425812
piR-hsa-1288731	Maternal to zygotic transition (MZT)	0.517425812
piR-hsa-1288731	Processing of DNA double-strand break ends	0.517425812
piR-hsa-1288731	HCMV Late Events	0.517425812
piR-hsa-1288731	Pre-NOTCH Transcription and Translation	0.517425812
piR-hsa-1288731	Replacement of protamines by nucleosomes in the male pronucleus	0.517425812
piR-hsa-1288731	DNA Double Strand Break Response	0.517425812

piR-hsa-1288731	ESR-mediated signaling	0.517425812
piR-hsa-1288731	DNA Replication	0.517425812
piR-hsa-1288731	DNA methylation	0.517425812
piR-hsa-1288731	Telomere Maintenance	0.517425812
piR-hsa-1288731	Recruitment and ATM-mediated phosphorylation of repair and signaling proteins at DNA double strand breaks	0.517425812
piR-hsa-1288731	Activation of HOX genes during differentiation	0.517425812
piR-hsa-1288731	Signaling by Rho GTPases	0.517425812
piR-hsa-1288731	Gene Silencing by RNA	0.517425812
piR-hsa-1288731	DNA Replication Pre-Initiation	0.517425812
piR-hsa-1288731	Inhibition of DNA recombination at telomere	0.517425812
piR-hsa-1288731	HDACs deacetylate histones	0.517425812
piR-hsa-1288731	Negative epigenetic regulation of rRNA expression	0.517425812
piR-hsa-1288731	NoRC negatively regulates rRNA expression	0.517425812
piR-hsa-1288731	HCMV Infection	0.517425812
piR-hsa-1288731	Assembly of the ORC complex at the origin of replication	0.517425812
piR-hsa-1288731	RUNX1 regulates genes involved in megakaryocyte differentiation and platelet function	0.517425812
piR-hsa-1288731	Chromatin organization	0.517425812
piR-hsa-1288731	Base-Excision Repair, AP Site Formation	0.517425812
piR-hsa-1288731	PRC2 methylates histones and DNA	0.517425812
piR-hsa-1288731	RMTs methylate histone arginines	0.517425812
piR-hsa-1288731	Activated PKN1 stimulates transcription of AR (androgen receptor) regulated genes KLK2 and KLK3	0.517425812
piR-hsa-1288731	RHO GTPases activate PKNs	0.517425812
piR-hsa-1288731	Cleavage of the damaged pyrimidine	0.517425812
piR-hsa-1288731	Condensation of Prophase Chromosomes	0.517425812
piR-hsa-1288731	Recognition and association of DNA glycosylase with site containing an affected purine	0.517425812
piR-hsa-1288731	G2/M Checkpoints	0.517425812
piR-hsa-1288731	ERCC6 (CSB) and EHMT2 (G9a) positively regulate rRNA expression	0.517425812
piR-hsa-1288731	B-WICH complex positively regulates rRNA expression	0.517425812
piR-hsa-1288731	Meiosis	0.517425812
piR-hsa-1288731	Signaling by Rho GTPases, Miro GTPases and RHOBTB3	0.517425812
piR-hsa-1288731	RNA Polymerase I Promoter Escape	0.517425812
piR-hsa-1288731	Meiotic recombination	0.517425812
piR-hsa-1288731	Deposition of new CENPA-containing nucleosomes at the centromere	0.517425812
piR-hsa-1288731	Cellular Senescence	0.517425812
piR-hsa-1288731	Formation of the beta-catenin:TCF transactivating complex	0.517425812
piR-hsa-1288731	Depyrimidination	0.432167508
piR-hsa-1288731	Chromosome Maintenance	0.432167508

piR-hsa-1288731	HDACs deacetylate histones	0.432167508
piR-hsa-1288731	HCMV Late Events	0.432167508
piR-hsa-1288731	DNA Replication Pre-Initiation	0.432167508
piR-hsa-1288731	Base Excision Repair	0.432167508
piR-hsa-1288731	Depurination	0.432167508
piR-hsa-1288731	Senescence-Associated Secretory Phenotype (SASP)	0.432167508
piR-hsa-1288731	SIRT1 negatively regulates rRNA expression	0.432167508
piR-hsa-1288731	HATs acetylate histones	0.432167508
piR-hsa-1288731	RUNX1 regulates transcription of genes involved in differentiation of HSCs	0.432167508
piR-hsa-1288731	Signaling by NOTCH	0.432167508
piR-hsa-1288731	Defective pyroptosis	0.432167508
piR-hsa-1288731	Inhibition of DNA recombination at telomere	0.432167508
piR-hsa-1288731	Amyloid fiber formation	0.432167508
piR-hsa-1288731	RNA Polymerase I Transcription	0.432167508
piR-hsa-1288731	Processing of DNA double-strand break ends	0.432167508
piR-hsa-1288731	Oxidative Stress Induced Senescence	0.432167508
piR-hsa-1288731	B-WICH complex positively regulates rRNA expression	0.432167508
piR-hsa-1288731	Packaging Of Telomere Ends	0.432167508
piR-hsa-1288731	Meiosis	0.432167508
piR-hsa-1288731	Pre-NOTCH Expression and Processing	0.432167508
piR-hsa-1288731	Meiotic synapsis	0.432167508
piR-hsa-1288731	Chromatin organization	0.432167508
piR-hsa-1288731	Condensation of Prophase Chromosomes	0.432167508
piR-hsa-1288731	Chromatin modifying enzymes	0.432167508
piR-hsa-1288731	Estrogen-dependent gene expression	0.432167508
piR-hsa-1288731	Epigenetic regulation of gene expression	0.432167508
piR-hsa-1288731	Transcriptional regulation by small RNAs	0.432167508
piR-hsa-1288731	DNA methylation	0.432167508
piR-hsa-1288731	Signaling by Nuclear Receptors	0.432167508
piR-hsa-1288731	RMTs methylate histone arginines	0.432167508
piR-hsa-1288731	Maternal to zygotic transition (MZT)	0.432167508
piR-hsa-1288731	Activation of HOX genes during differentiation	0.432167508
piR-hsa-1288731	Gene Silencing by RNA	0.432167508
piR-hsa-1288731	Negative epigenetic regulation of rRNA expression	0.432167508
piR-hsa-1288731	RNA Polymerase I Promoter Opening	0.432167508
piR-hsa-1288731	Cleavage of the damaged pyrimidine	0.432167508
piR-hsa-1288731	G2/M Checkpoints	0.432167508

piR-hsa-1288731	Signaling by WNT	0.432167508
piR-hsa-1288731	Nonhomologous End-Joining (NHEJ)	0.432167508
piR-hsa-1288731	Recognition and association of DNA glycosylase with site containing an affected pyrimidine	0.432167508
piR-hsa-1288731	Signaling by Rho GTPases	0.432167508
piR-hsa-1288731	Deposition of new CENPA-containing nucleosomes at the centromere	0.432167508
piR-hsa-1288731	Chromatin modifications during the maternal to zygotic transition (MZT)	0.432167508
piR-hsa-1288731	DNA Damage/Telomere Stress Induced Senescence	0.432167508
piR-hsa-1288731	Base-Excision Repair, AP Site Formation	0.432167508
piR-hsa-1288731	RNA Polymerase I Promoter Clearance	0.432167508
piR-hsa-1288731	Positive epigenetic regulation of rRNA expression	0.432167508
piR-hsa-1288731	Activated PKN1 stimulates transcription of AR (androgen receptor) regulated genes KLK2 and KLK3	0.432167508
piR-hsa-1288731	RHO GTPases activate PKNs	0.432167508
piR-hsa-1288731	PRC2 methylates histones and DNA	0.432167508
piR-hsa-1288731	RUNX1 regulates genes involved in megakaryocyte differentiation and platelet function	0.432167508
piR-hsa-1288731	Assembly of the pre-replicative complex	0.432167508
piR-hsa-1288731	TCF dependent signaling in response to WNT	0.432167508
piR-hsa-1288731	Telomere Maintenance	0.432167508
piR-hsa-1288731	Activation of anterior HOX genes in hindbrain development during early embryogenesis	0.432167508
piR-hsa-1288731	ESR-mediated signaling	0.432167508
piR-hsa-1288731	DNA Double Strand Break Response	0.432167508
piR-hsa-1288731	Formation of the beta-catenin:TCF transactivating complex	0.432167508
piR-hsa-1288731	Nucleosome assembly	0.432167508
piR-hsa-1288731	RHO GTPase Effectors	0.432167508
piR-hsa-1288731	Recruitment and ATM-mediated phosphorylation of repair and signaling proteins at DNA double strand breaks	0.432167508
piR-hsa-1288731	Recognition and association of DNA glycosylase with site containing an affected purine	0.432167508
piR-hsa-1288731	Reproduction	0.432167508
piR-hsa-1288731	HCMV Infection	0.432167508
piR-hsa-1288731	Mitotic Prophase	0.432167508
piR-hsa-1288731	Transcriptional regulation of granulopoiesis	0.432167508
piR-hsa-1288731	NoRC negatively regulates rRNA expression	0.432167508
piR-hsa-1288731	DNA Replication	0.432167508
piR-hsa-1288731	HCMV Early Events	0.432167508
piR-hsa-1288731	Cellular Senescence	0.432167508
piR-hsa-1288731	Pre-NOTCH Transcription and Translation	0.432167508
piR-hsa-1288731	Diseases of programmed cell death	0.432167508
piR-hsa-1288731	ERCC6 (CSB) and EHMT2 (G9a) positively regulate rRNA expression	0.432167508
piR-hsa-1288731	Cleavage of the damaged purine	0.432167508

piR-hsa-1288731	Cell Cycle Checkpoints	0.432167508
piR-hsa-1288731	RNA Polymerase I Promoter Escape	0.432167508
piR-hsa-1288731	Assembly of the ORC complex at the origin of replication	0.432167508
piR-hsa-1288731	Signaling by Rho GTPases, Miro GTPases and RHOBTB3	0.432167508
piR-hsa-1288731	G2/M DNA damage checkpoint	0.432167508
piR-hsa-1288731	Meiotic recombination	0.432167508
piR-hsa-1288731	Replacement of protamines by nucleosomes in the male pronucleus	0.432167508
piR-hsa-2499988	Glycolysis	0.407762319
piR-hsa-141155	Glycolysis	0.495598642
piR-hsa-141155	Glycolysis	0.460514101
piR-hsa-141155	Regulation of Homotypic Cell-Cell Adhesion	0.390878308
piR-hsa-2851799	Regulation of Homotypic Cell-Cell Adhesion	0.427828494
piR-hsa-141155	Regulation of Expression and Function of Type II Classical Cadherins	0.390878308
piR-hsa-2851799	Regulation of Expression and Function of Type II Classical Cadherins	0.427828494
piR-hsa-141155	Regulation of CDH11 gene transcription	0.390878308
piR-hsa-2851799	Regulation of CDH11 gene transcription	0.427828494
piR-hsa-141155	Cell junction organization	0.390878308
piR-hsa-2851799	Cell junction organization	0.427828494
piR-hsa-141155	Adherens junctions interactions	0.390878308
piR-hsa-2851799	Adherens junctions interactions	0.427828494
piR-hsa-141155	Regulation of CDH11 Expression and Function	0.390878308
piR-hsa-2851799	Regulation of CDH11 Expression and Function	0.427828494
piR-hsa-141155	Reproduction	0.42952548
piR-hsa-2499988	Reproduction	0.399825576
piR-hsa-141155	Collagen formation	0.411303155
piR-hsa-141155	Assembly of collagen fibrils and other multimeric structures	0.411303155
piR-hsa-141155	Cell junction organization	0.411303155
piR-hsa-141155	Signaling by NOTCH	0.462329802
piR-hsa-141155	Signaling by Rho GTPases	0.462329802
piR-hsa-141155	Signaling by Rho GTPases, Miro GTPases and RHOBTB3	0.462329802
piR-hsa-2851799	RHO GTPase Effectors	0.396986179
piR-hsa-141155	RHO GTPase Effectors	0.424146271
piR-hsa-2851799	Signaling by Rho GTPases	0.396986179
piR-hsa-141155	Signaling by Rho GTPases	0.424146271
piR-hsa-2851799	Signaling by Rho GTPases, Miro GTPases and RHOBTB3	0.396986179
piR-hsa-141155	Signaling by Rho GTPases, Miro GTPases and RHOBTB3	0.424146271
piR-hsa-141155	Signaling by Rho GTPases	0.524155288

piR-hsa-2499988	Signaling by Rho GTPases	0.381120607
piR-hsa-141155	Signaling by Rho GTPases, Miro GTPases and RHOBTB3	0.524155288
piR-hsa-2499988	Signaling by Rho GTPases, Miro GTPases and RHOBTB3	0.381120607
piR-hsa-2851799	Cellular Senescence	0.855120149
piR-hsa-2499988	Cellular Senescence	0.511288946
piR-hsa-141155	Cellular Senescence	0.540339705
piR-hsa-2851799	Oxidative Stress Induced Senescence	0.855120149
piR-hsa-2499988	Oxidative Stress Induced Senescence	0.511288946
piR-hsa-141155	Oxidative Stress Induced Senescence	0.540339705
piR-hsa-2851799	Signaling by Nuclear Receptors	0.855120149
piR-hsa-2499988	Signaling by Nuclear Receptors	0.511288946
piR-hsa-141155	Signaling by Nuclear Receptors	0.540339705
piR-hsa-2851799	RHO GTPase Effectors	0.855120149
piR-hsa-2499988	RHO GTPase Effectors	0.511288946
piR-hsa-141155	RHO GTPase Effectors	0.540339705
piR-hsa-2851799	Mitotic Prophase	0.855120149
piR-hsa-2499988	Mitotic Prophase	0.511288946
piR-hsa-141155	Mitotic Prophase	0.540339705
piR-hsa-2851799	ESR-mediated signaling	0.855120149
piR-hsa-2499988	ESR-mediated signaling	0.511288946
piR-hsa-141155	ESR-mediated signaling	0.540339705
piR-hsa-2851799	Signaling by Rho GTPases, Miro GTPases and RHOBTB3	0.855120149
piR-hsa-2499988	Signaling by Rho GTPases, Miro GTPases and RHOBTB3	0.511288946
piR-hsa-141155	Signaling by Rho GTPases, Miro GTPases and RHOBTB3	0.540339705
piR-hsa-2851799	Signaling by Rho GTPases	0.855120149
piR-hsa-2499988	Signaling by Rho GTPases	0.511288946
piR-hsa-141155	Signaling by Rho GTPases	0.540339705
piR-hsa-2851799	Senescence-Associated Secretory Phenotype (SASP)	0.855120149
piR-hsa-2499988	Senescence-Associated Secretory Phenotype (SASP)	0.511288946
piR-hsa-141155	Senescence-Associated Secretory Phenotype (SASP)	0.540339705
piR-hsa-141155	Cellular Senescence	0.387322093
piR-hsa-141155	Oxidative Stress Induced Senescence	0.387322093
piR-hsa-141155	Signaling by Rho GTPases	0.387322093
piR-hsa-141155	Signaling by Rho GTPases, Miro GTPases and RHOBTB3	0.387322093
piR-hsa-141155	RHO GTPase Effectors	0.387322093
piR-hsa-141155	Collagen degradation	0.473616582
piR-hsa-2499988	Collagen degradation	0.408739422

piR-hsa-2851799	Collagen degradation	0.794158198
piR-hsa-141155	Collagen degradation	0.4868278
piR-hsa-2499988	Collagen formation	0.408739422
piR-hsa-2851799	Collagen formation	0.794158198
piR-hsa-141155	Collagen formation	0.4868278
piR-hsa-2499988	ESR-mediated signaling	0.408739422
piR-hsa-2851799	ESR-mediated signaling	0.794158198
piR-hsa-141155	ESR-mediated signaling	0.4868278
piR-hsa-2499988	Assembly of collagen fibrils and other multimeric structures	0.408739422
piR-hsa-2851799	Assembly of collagen fibrils and other multimeric structures	0.794158198
piR-hsa-141155	Assembly of collagen fibrils and other multimeric structures	0.4868278
piR-hsa-2499988	Signaling by Nuclear Receptors	0.408739422
piR-hsa-2851799	Signaling by Nuclear Receptors	0.794158198
piR-hsa-141155	Signaling by Nuclear Receptors	0.4868278
piR-hsa-141155	Chromatin modifying enzymes	0.39448111
piR-hsa-141155	Chromatin organization	0.39448111
piR-hsa-141155	HATs acetylate histones	0.39448111
piR-hsa-2851799	Positive epigenetic regulation of rRNA expression	0.448513376
piR-hsa-141155	Positive epigenetic regulation of rRNA expression	0.416600941
piR-hsa-2851799	B-WICH complex positively regulates rRNA expression	0.448513376
piR-hsa-141155	B-WICH complex positively regulates rRNA expression	0.416600941
piR-hsa-2851799	Epigenetic regulation of gene expression	0.448513376
piR-hsa-141155	Epigenetic regulation of gene expression	0.416600941
piR-hsa-141155	Signaling by Nuclear Receptors	0.408932133
piR-hsa-141155	Signaling by WNT	0.408932133
piR-hsa-141155	Signaling by NOTCH	0.408932133
piR-hsa-141155	Deubiquitination	0.408932133
piR-hsa-141155	Ub-specific processing proteases	0.408932133
piR-hsa-141155	Estrogen-dependent gene expression	0.408932133
piR-hsa-141155	TCF dependent signaling in response to WNT	0.408932133
piR-hsa-141155	ESR-mediated signaling	0.408932133
piR-hsa-141155	Formation of the beta-catenin:TCF transactivating complex	0.408932133
piR-hsa-141155	Transcriptional regulation of granulopoiesis	0.408932133
piR-hsa-141155	Signaling by Rho GTPases	0.422969988
piR-hsa-141155	RHO GTPase Effectors	0.422969988
piR-hsa-141155	Signaling by Rho GTPases, Miro GTPases and RHOBTB3	0.422969988
piR-hsa-141155	RHO GTPases activate PKNs	0.422969988

piR-hsa-2499988	ESR-mediated signaling	0.394915561
piR-hsa-2499988	Estrogen-dependent gene expression	0.394915561
piR-hsa-2499988	Activation of anterior HOX genes in hindbrain development during early embryogenesis	0.394915561
piR-hsa-2499988	Signaling by Nuclear Receptors	0.394915561
piR-hsa-2499988	Activation of HOX genes during differentiation	0.394915561
piR-hsa-141155	RHO GTPase Effectors	0.429563293
piR-hsa-141155	Signaling by Rho GTPases	0.429563293
piR-hsa-141155	Signaling by Rho GTPases, Miro GTPases and RHOBTB3	0.429563293
piR-hsa-141155	Association of TriC/CCT with target proteins during biosynthesis	0.45888182
piR-hsa-141155	Protein folding	0.45888182
piR-hsa-141155	Chaperonin-mediated protein folding	0.45888182
piR-hsa-141155	Pre-NOTCH Transcription and Translation	0.386475964
piR-hsa-2499988	Pre-NOTCH Transcription and Translation	0.396499925
piR-hsa-141155	Signaling by NOTCH	0.386475964
piR-hsa-2499988	Signaling by NOTCH	0.396499925
piR-hsa-141155	Pre-NOTCH Expression and Processing	0.386475964
piR-hsa-2499988	Pre-NOTCH Expression and Processing	0.396499925
piR-hsa-2851799	Signaling by Rho GTPases	0.538326679
piR-hsa-2851799	RHO GTPase Effectors	0.538326679
piR-hsa-2851799	Signaling by WNT	0.538326679
piR-hsa-2851799	Signaling by Rho GTPases, Miro GTPases and RHOBTB3	0.538326679
piR-hsa-141155	Glycolysis	0.468612135
piR-hsa-2499988	Glycolysis	0.424467965
piR-hsa-2499988	Glycolysis	0.437225274
piR-hsa-141155	Glycolysis	0.510742657
piR-hsa-141155	G2/M Checkpoints	0.404524903
piR-hsa-141155	Cell Cycle Checkpoints	0.404524903
piR-hsa-141155	Collagen formation	0.420488691
piR-hsa-141155	Cell junction organization	0.420488691
piR-hsa-141155	Assembly of collagen fibrils and other multimeric structures	0.420488691
piR-hsa-141155	Signaling by Rho GTPases, Miro GTPases and RHOBTB3	0.378353328
piR-hsa-141155	Signaling by Rho GTPases	0.378353328
piR-hsa-141155	Signaling by Nuclear Receptors	0.528708224
piR-hsa-2851799	Signaling by Nuclear Receptors	0.409950114
piR-hsa-2499988	Signaling by Nuclear Receptors	0.42581849
piR-hsa-141155	Signaling by NOTCH	0.406465201
piR-hsa-141155	G2/M Checkpoints	0.406465201

piR-hsa-141155	DNA Replication	0.406465201
piR-hsa-141155	Cell Cycle Checkpoints	0.406465201
piR-hsa-141155	Signaling by WNT	0.406465201
piR-hsa-141155	TCF dependent signaling in response to WNT	0.406465201
piR-hsa-141155	Ub-specific processing proteases	0.406465201
piR-hsa-141155	UCH proteinases	0.406465201
piR-hsa-141155	RUNX1 regulates transcription of genes involved in differentiation of HSCs	0.406465201
piR-hsa-141155	Deubiquitination	0.406465201
piR-hsa-141155	Assembly of the pre-replicative complex	0.406465201
piR-hsa-141155	DNA Replication Pre-Initiation	0.406465201
piR-hsa-2499988	Signaling by Rho GTPases	0.389937507
piR-hsa-141155	Signaling by Rho GTPases	0.461723748
piR-hsa-2499988	Signaling by Rho GTPases, Miro GTPases and RHOBTB3	0.389937507
piR-hsa-141155	Signaling by Rho GTPases, Miro GTPases and RHOBTB3	0.461723748
piR-hsa-2499988	RHO GTPase Effectors	0.389937507
piR-hsa-141155	RHO GTPase Effectors	0.461723748
piR-hsa-2499988	Cell Cycle Checkpoints	0.389937507
piR-hsa-141155	Cell Cycle Checkpoints	0.461723748
piR-hsa-2851799	Signaling by Nuclear Receptors	0.796718358
piR-hsa-2499988	Signaling by Nuclear Receptors	0.410290441
piR-hsa-141155	Signaling by Nuclear Receptors	0.464113517
piR-hsa-2851799	Amyloid fiber formation	0.527961897
piR-hsa-2499988	G2/M DNA damage checkpoint	0.394612597
piR-hsa-2499988	G2/M Checkpoints	0.394612597
piR-hsa-2499988	RHO GTPases activate PKNs	0.394612597
piR-hsa-2499988	Cell Cycle Checkpoints	0.394612597
piR-hsa-2499988	Signaling by Rho GTPases	0.394612597
piR-hsa-2499988	Signaling by Rho GTPases, Miro GTPases and RHOBTB3	0.394612597
piR-hsa-2499988	RHO GTPase Effectors	0.394612597
piR-hsa-141155	Signaling by Rho GTPases	0.378420573
piR-hsa-141155	Signaling by Rho GTPases, Miro GTPases and RHOBTB3	0.378420573
piR-hsa-2499988	Signaling by WNT	0.414726723
piR-hsa-141155	Signaling by WNT	0.405146675
piR-hsa-2499988	TCF dependent signaling in response to WNT	0.414726723
piR-hsa-141155	TCF dependent signaling in response to WNT	0.405146675
piR-hsa-141155	Signaling by Nuclear Receptors	0.554414684
piR-hsa-2851799	Signaling by Nuclear Receptors	0.441404854

piR-hsa-2499988	Signaling by Nuclear Receptors	0.457187269
piR-hsa-141155	Chaperonin-mediated protein folding	0.554414684
piR-hsa-2851799	Chaperonin-mediated protein folding	0.441404854
piR-hsa-2499988	Chaperonin-mediated protein folding	0.457187269
piR-hsa-141155	Protein folding	0.554414684
piR-hsa-2851799	Protein folding	0.441404854
piR-hsa-2499988	Protein folding	0.457187269
piR-hsa-141155	Association of TriC/CCT with target proteins during biosynthesis	0.554414684
piR-hsa-2851799	Association of TriC/CCT with target proteins during biosynthesis	0.441404854
piR-hsa-2499988	Association of TriC/CCT with target proteins during biosynthesis	0.457187269
piR-hsa-141155	ESR-mediated signaling	0.554414684
piR-hsa-2851799	ESR-mediated signaling	0.441404854
piR-hsa-2499988	ESR-mediated signaling	0.457187269
piR-hsa-141155	Deubiquitination	0.409864281
piR-hsa-141155	UCH proteinases	0.409864281
piR-hsa-2499988	Signaling by Rho GTPases, Miro GTPases and RHOBTB3	0.40779697
piR-hsa-2499988	Protein folding	0.40779697
piR-hsa-2499988	Formation of tubulin folding intermediates by CCT/TriC	0.40779697
piR-hsa-2499988	Signaling by Rho GTPases	0.40779697
piR-hsa-2499988	Cooperation of Prefoldin and TriC/CCT in actin and tubulin folding	0.40779697
piR-hsa-2499988	Chaperonin-mediated protein folding	0.40779697
piR-hsa-2499988	Prefoldin mediated transfer of substrate to CCT/TriC	0.40779697
piR-hsa-2499988	HCMV Infection	0.40779697
piR-hsa-2499988	HCMV Early Events	0.40779697
piR-hsa-2499988	RHO GTPase Effectors	0.40779697
piR-hsa-141155	Deubiquitination	0.398922539
piR-hsa-141155	Cell Cycle Checkpoints	0.398922539
piR-hsa-141155	Signaling by WNT	0.398922539
piR-hsa-141155	Oxidative Stress Induced Senescence	0.398922539
piR-hsa-141155	Cellular Senescence	0.398922539
piR-hsa-141155	Senescence-Associated Secretory Phenotype (SASP)	0.398922539
piR-hsa-141155	Ub-specific processing proteases	0.398922539
piR-hsa-141155	Recruitment and ATM-mediated phosphorylation of repair and signaling proteins at DNA double strand breaks	0.398922539
piR-hsa-141155	RUNX1 regulates transcription of genes involved in differentiation of HSCs	0.398922539
piR-hsa-141155	DNA Double Strand Break Response	0.398922539
piR-hsa-141155	UCH proteinases	0.398922539
piR-hsa-141155	Amyloid fiber formation	0.398922539

piR-hsa-141155	Signaling by NOTCH	0.398922539
piR-hsa-141155	DNA Replication Pre-Initiation	0.398922539
piR-hsa-141155	G2/M Checkpoints	0.398922539
piR-hsa-141155	Metalloprotease DUBs	0.398922539
piR-hsa-141155	Protein ubiquitination	0.398922539
piR-hsa-141155	Processing of DNA double-strand break ends	0.398922539
piR-hsa-141155	Assembly of the pre-replicative complex	0.398922539
piR-hsa-141155	E3 ubiquitin ligases ubiquitinate target proteins	0.398922539
piR-hsa-141155	DNA Replication	0.398922539
piR-hsa-141155	TCF dependent signaling in response to WNT	0.398922539
piR-hsa-141155	E3 ubiquitin ligases ubiquitinate target proteins	0.514156289
piR-hsa-2499988	E3 ubiquitin ligases ubiquitinate target proteins	0.493383408
piR-hsa-2851799	E3 ubiquitin ligases ubiquitinate target proteins	0.901834679
piR-hsa-141155	Protein ubiquitination	0.514156289
piR-hsa-2499988	Protein ubiquitination	0.493383408
piR-hsa-2851799	Protein ubiquitination	0.901834679
piR-hsa-141155	TCF dependent signaling in response to WNT	0.493722677
piR-hsa-141155	Signaling by WNT	0.493722677
piR-hsa-141155	RHO GTPase Effectors	0.493722677
piR-hsa-141155	Cell Cycle Checkpoints	0.493722677
piR-hsa-141155	RHO GTPases activate PKNs	0.493722677
piR-hsa-141155	G2/M DNA damage checkpoint	0.493722677
piR-hsa-141155	Signaling by Rho GTPases, Miro GTPases and RHOBTB3	0.493722677
piR-hsa-141155	Signaling by NOTCH	0.493722677
piR-hsa-141155	Signaling by Rho GTPases	0.493722677
piR-hsa-141155	G2/M Checkpoints	0.493722677
piR-hsa-2499988	Cell Cycle Checkpoints	0.45577376
piR-hsa-141155	Cell Cycle Checkpoints	0.433277526
piR-hsa-141155	Cell junction organization	0.377764806
